# Associating somatic mutations to clinical outcomes: a pan-cancer study of survival time

**DOI:** 10.1186/s13073-019-0643-9

**Published:** 2019-05-28

**Authors:** Paul Little, Dan-Yu Lin, Wei Sun

**Affiliations:** 1 Public Health Sciences Division, Fred Hutchinson Cancer Research Center, 1100 Fairview Ave N, Seattle, 98109 WA USA; 2 Department of Biostatistics, University of North Carolina Chapel Hill, Dauer Drive, Chapel Hill, 27599 NC USA; 3 Department of Biostatistics, University of Washington, NE Pacific St, Seattle, 98195 WA USA

**Keywords:** Copy number alteration, Intra-tumor heterogeneity, Somatic mutations, Subclone, Tumor mutation burden

## Abstract

We developed subclone multiplicity allocation and somatic heterogeneity (SMASH), a new statistical method for intra-tumor heterogeneity (ITH) inference. SMASH is tailored to the purpose of large-scale association studies with one tumor sample per patient. In a pan-cancer study of 14 cancer types, we studied the associations between survival time and ITH quantified by SMASH, together with other features of somatic mutations. Our results show that ITH is associated with survival time in several cancer types and its effect can be modified by other covariates, such as mutation burden. SMASH is available at https://github.com/Sun-lab/SMASH.

**Electronic supplementary material:**

The online version of this article (10.1186/s13073-019-0643-9) contains supplementary material, which is available to authorized users.

## Background

Somatic mutations, including somatic point mutations (SPMs; e.g., single nucleotide variants or indels) and somatic copy number alterations (SCNAs), are the underlying driving force for tumor growth. In this sense, cancer is a genetic disease. Therefore, association studies between somatic mutations and clinical outcomes may provide insights into tumor biology or personalized treatment selection. However, few efforts have been reported toward this end, partly because most somatic mutations or even gene-level mutations are too rare to conduct meaningful association studies. An alternative to a mutation-by-mutation or gene-by-gene association study is to summarize mutation information by certain features and then associate such features with clinical outcomes. In this paper, we consider three such features: tumor mutation burden (TMB, i.e., the total number of SPMs), SCNA burden, and the degree of (genetic) intra-tumor heterogeneity (ITH), which refers to the fact that tumor cells can be grouped in subclones such that the cells within one subclone share similar sets of somatic mutations. ITH is a fundamental characteristic of somatic mutations and has been associated with clinical outcomes such as survival time or immunotherapy treatment response [1, 2].

We estimate TMB by counting the number of non-synonymous point mutations [3, 4] and estimate the burden of SCNAs using allele-specific copy number estimates derived from ASCAT [5]. While measuring TMB and SCNA burden is relatively straightforward, quantifying ITH is much more challenging. Computational methods have been developed to characterize ITH, e.g., to identify the phylogenetic tree of subclones and the mutations belonging to each subclone [6–11]. However, consensus on the optimal approach for ITH inference and the appropriate approach for quantifying ITH in association studies does not exist. The estimation uncertainty of ITH is often unavoidable because the observed data may be compatible with more than one subclone configuration. Therefore, such uncertainty should be incorporated in association studies.

Counting the number of subclones is a straightforward approach to quantify ITH. Andor et al. [1] assessed the association between the number of subclones and survival time in 12 cancer types using data derived from The Cancer Genome Atlas (TCGA). These investigators did not find any significant associations, except for gliomas. Morris et al. [12] assessed the association between ITH and survival time in nine cancer types and found significant associations for several cancer types. They treated ITH as a binary variable based on whether or not the number of subclones was larger than a threshold. An apparent drawback of the aforementioned two approaches is that the subclone proportion information is lost. For example, if a tumor sample has two subclones with cellular proportions being 99% and 1%. Intuitively, this tumor sample is fairly homogenous and may be better classified as one subclone instead of two subclones. A second drawback of the thresholding approach of Morris et al. [12] was that only a small number of patients (3 to 11 patients across nine cancer types, median of six patients) were classified as having both high ITH and non-censored survival time. As a result, the association results can be highly unstable with respect to ITH inference.

**Fig. 1:**
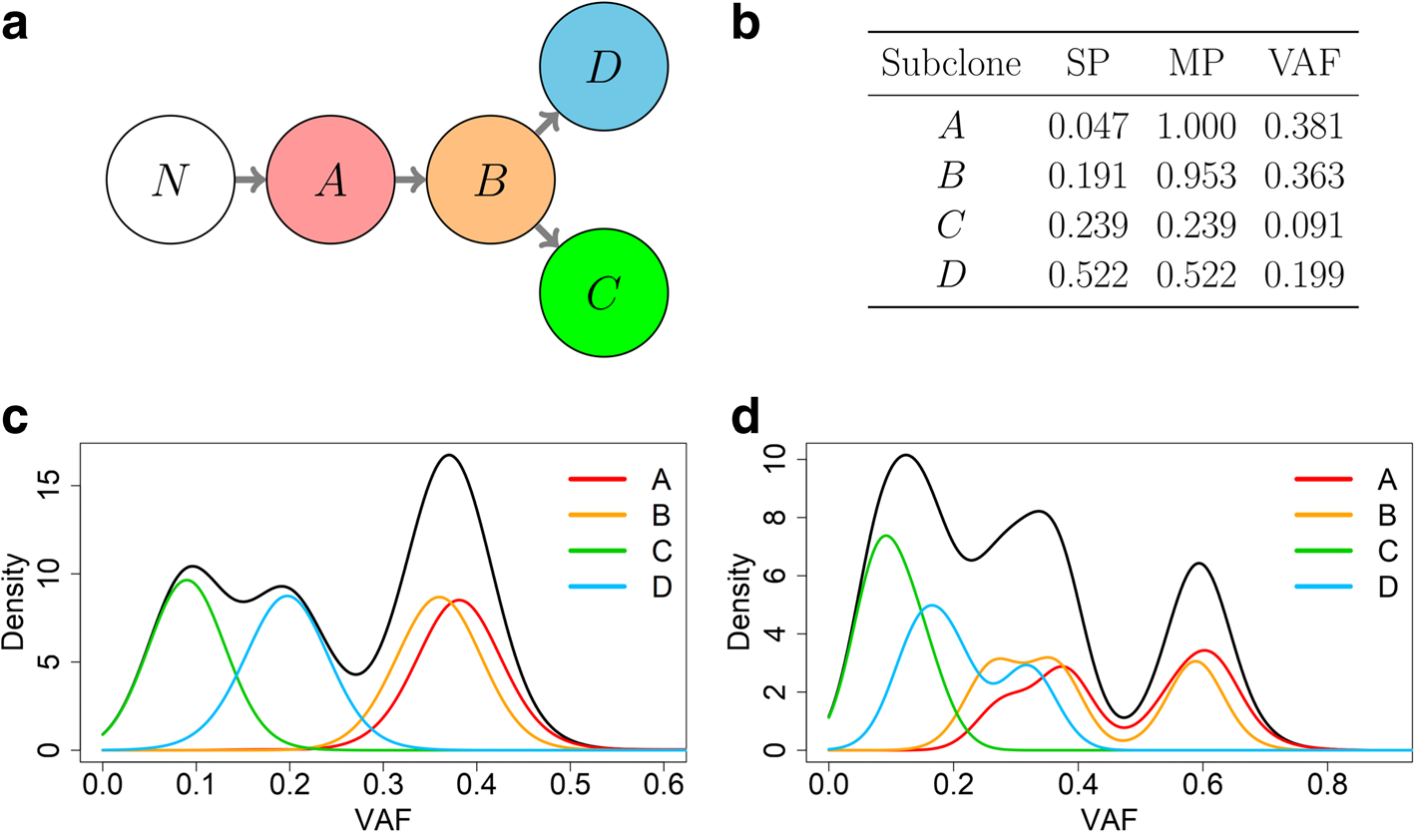
ITH example with and without CNAs. **a** Visualization of a tree, where each node represents a subclone within a tumor sample. *N* denotes the normal cells, and *A*, *B*, *C*, and *D* denote the descending subclones. To simplify notation, we also use *A*, *B*, *C*, and *D* to denote the mutations that arise from the corresponding four subclones. We simulated a tumor purity of 0.762 with 1000 variants under the following scenarios: (1) no somatic copy number alterations (SCNAs) and (2) SCNAs in which mutations are equally distributed across clonal copy number states (0,1), (1,1), and (1,2). A copy number state denotes the number of copies of the two alleles. For example, copy number state (0,1) denotes deletion in one allele. **b** The second column corresponds to the cellular proportions of each subclone after accounting for tumor purity. The third and fourth columns correspond to the cellular prevalence and mean VAF (without SCNAs), respectively, of the mutations arising from each subclone. In (**c**) and (**d**), the black curve is the overall VAF density, and the colored curves are the subclone-specific VAF densities. Multiple subclone-specific VAF peaks with SCNA are due to combinations of multiplicity and subclone allocation

An alternative metric to quantify ITH is mutant-allele tumor heterogeneity (MATH) [13], which is defined as 100×MAD/median, where median is the median of the variant allele frequencies (VAFs) of all somatic point mutations within a sample, and MAD is the median absolute deviation of the VAFs. MATH pertains to the ratio between the center and spread of the VAF distribution. This approach ignores the fact that VAF can be affected by SCNAs (see Fig. 1 for an illustration).

Although many methods have been developed for ITH inference, none of them are ideal for large-scale association studies. In most solid tumors, a significant proportion of the genome is affected by SCNA, and so, those methods that cannot account for SCNA [8, 14–17] are not appropriate for our purpose. Several methods either explicitly or implicitly require multiple samples per patient [6, 8, 10, 14, 15, 17] and thus cannot be used for our association analysis of TCGA data, where each patient only has one sample.

PyClone [11] is arguably the most popular method for ITH study and has been used in two pan-cancer studies [1, 12]. However, PyClone is designed for targeted sequencing studies, where a small number of loci are sequenced with ultra-high coverage (e.g., >1,000 × coverage). Its Bayesian Markov Chain Monte Carlo (MCMC) implementation requires an extended runtime. In addition, PyClone performs clustering of somatic mutations, but does not infer phylogeny.

Many other existing methods for ITH study [7, 9–11] also use Bayesian MCMC implementation and their computational burden makes them undesirable for large-scale association studies. Another class of methods use combinatorial approaches [6, 16, 17]. Several approaches do not account for SCNA [16, 17]. SPRUCE [6], a more recent algorithm, jointly models SPMs and SCNAs by multi-state perfect phylogeny mixtures. It is designed for multi-sample study with a small number of mutations or mutation clusters. For example, as shown in their simulations, even with only 5 mutations or mutation clusters, at 500 × coverage, the median number of solutions is between 1000 and 10,000 when there are two samples, and around 100 when there are 5 samples. Other ITH quantification methods either do not provide an easy-to-use uncertainty measurement [18] or require additional (hard-to-get) information such as phasing between sparsely distributed somatic mutations [19].

Given these considerations, we developed a new method for ITH study, called subclone multiplicity allocation and somatic heterogeneity or SMASH. To overcome the limitations of the aforementioned approaches for quantifying ITH, we quantify ITH, as previous studies have done [12, 20], using entropy $-\sum _{s=1}^{S} \vartheta _{s} \log {(\vartheta _{s})}$, where *𝜗*_*s*_ is the proportion of tumor cells that belong to the *s*th subclone and *S* is the total number of subclones. We assessed the performance of SMASH and a few other methods in large-scale simulated association analysis. Then we used these methods to study the association between survival time and TMB, SCNA burden, frequently mutated genes, and ITH using data on 5898 TCGA tumor samples from 14 cancer types [21].

The major contributions of our work are threefold. First, we propose a new computational method that is designed for large-scale studies of ITH with higher computational efficiency. Second, we evaluated the benefit to incorporate uncertainty of ITH estimates in association studies and conclude that there is positive but relatively minor benefit. Third, in the large-scale real data analysis, we found several interesting patterns such as the interaction between mutation burden and ITH.

## Methods

### Assumptions

SMASH is a frequentist approach to identify tumor subclones through clustering somatic mutation read counts, while accounting for copy number alterations. We enumerate all possible phylogenetic trees that are compatible with the observed data and quantify the probability of each phylogenetic tree. We make the following assumptions when enumerating phylogenic trees.

1. Primary tumors arise from a founder clone or have unicellular origin.
2. Loci harboring SPMs associated with ITH have homozygous reference alleles in normal cells and a mixture of reference and alternate alleles in tumor.
3. Each SPM event occurs only once on a single allele and a locus will not undergo more than one point mutation or revert back to its original base.
4. At most two descendant subclones can evolve from an ancestral subclone.
5. SCNAs are clonal events.

Assumption (1) follows from the clonal evolution theory of tumor growth [22]. Assumption (2) is automatically satisfied because genetic loci with germline mutations are filtered out during somatic mutation calling. Assumption (3) is referred to as the infinite site assumption [23, 24], which is reasonable because the number of mutated loci is very small relative to the size of the genome. This assumption implies that tumor evolution is consistent with a “perfect and persistent phylogeny” [9, 11] such that each subclone has only one parental subclone and all mutations of the parental subclone. Assumption (4) is reasonable when we consider tumor evolution in a refined time scale, and it is helpful to reduce the number of enumerated phylogenies. Assumption (5) is the only restrictive one, and it is a crucial assumption made by ASCAT [5], which is the method we use to infer copy numbers. Assumption (5) is also adopted by PyClone [11] and EXPANDS [18], the two methods that have been used in previous pan-cancer studies [1, 12]. To the best of our knowledge, Canopy [10] is the only method that can infer both subclonal SCNA and subclonal point mutations. However, Canopy carries a high computational cost and emphasizes multiple sample design, which makes it unsuitable for our study. By assuming clonal SCNA, all subclonal SPMs occur after the SCNA event and thus have a multiplicity of one. On the other hand, clonal SPMs can occur before or after SCNA and thus can have varying multiplicities, depending on the copy number state. We obtain SCNA-related information, including tumor purity, ploidy, and allele-specific copy numbers per SPM through ASCAT [5].

### Notation and framework

Let *T* and $\tilde {T}$ denote the failure time and the corresponding censoring time, respectively. Define $X = \min (T,\tilde {T})$ and $\Delta = I(T \leq \tilde {T})$. Let ***Z***=(*Z*_1_,…,*Z*_*p*_)^T^ represent a *p*-vector of baseline covariates. Let *l*=1,…,*L* index each locus harboring a SPM after mutation calling and filtering. The *l*th SPM is characterized by a pair of alternate and reference read counts derived from the tumor sample denoted by *A*_*l*_ and *R*_*l*_, respectively. The summation *T*_*l*_=*A*_*l*_+*R*_*l*_ is referred to as the total read depth. The corresponding clonal copy number state is denoted by (*C*_*l*1_,*C*_*l*2_), where *C*_*l*1_≤*C*_*l*2_. For a given subject, the observed clinical data consist of (*X*,*Δ*,***Z***), and genomic data are represented by (*A*_*l*_,*R*_*l*_,*C*_*l*1_,*C*_*l*2_) for *l*=1,…,*L*.

**Fig. 2:**
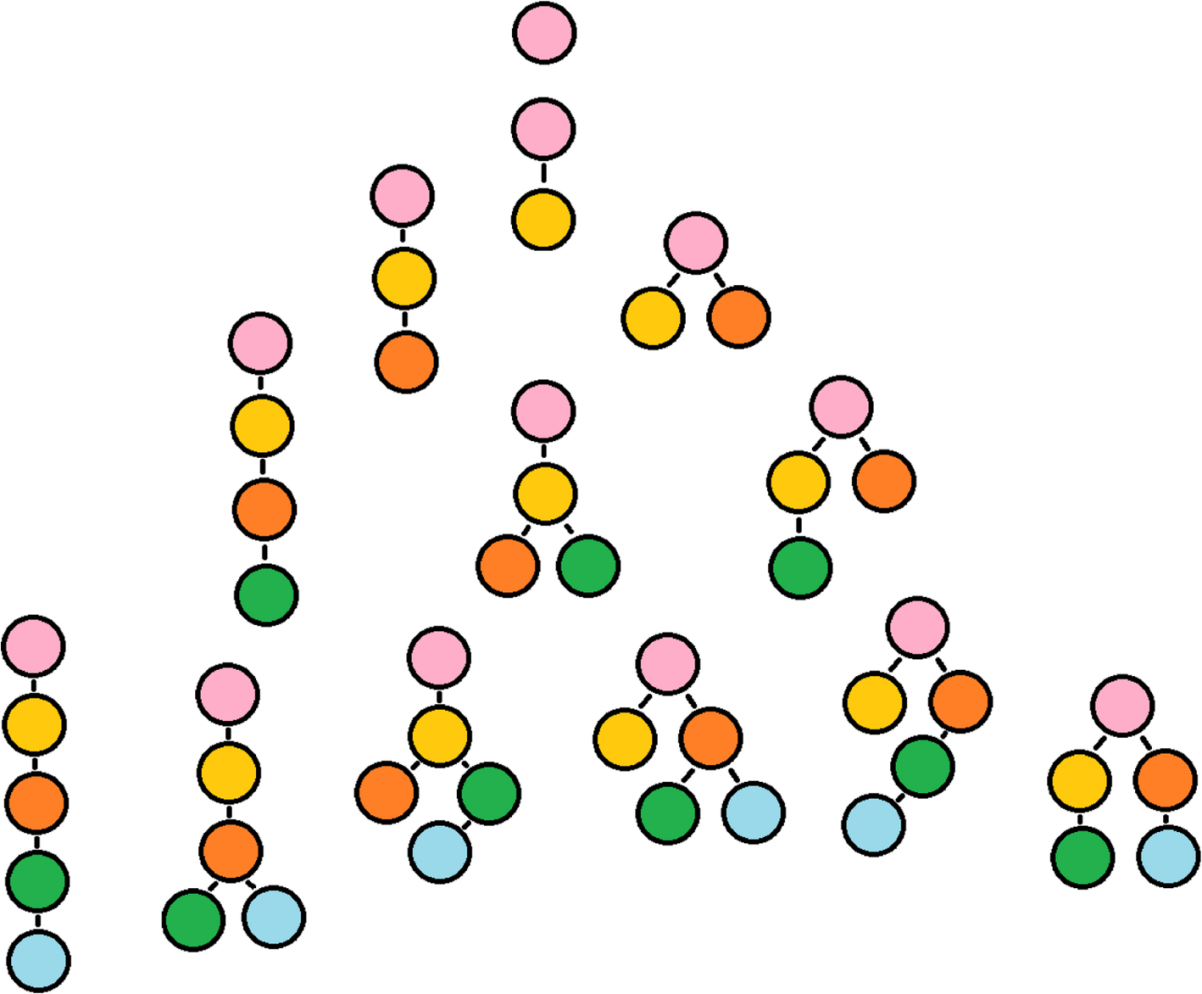
Subclone configurations. Examples of subclone configurations with subclone numbers ranging from 1 to 5. Nodes represent subclones, and vertices link the parental and descendant subclones

Assume that the tumor sample of interest has *S* subclones. These *S* subclones relate to each other through a phylogenic tree describing the order in which subclones emerged. In Fig. 2, we enumerated all phylogenic trees for one to five subclones that capture the possible linear and branching evolutions between subclones. A possible allocation of somatic mutations across the *S* subclones can be described by a vector of length *S*: $\boldsymbol {q}_{u}^{T} = (q_{u1}, \ldots,q_{uS})$ such that *q*_*us*_ is an indicator of whether this mutation occurs in the *s*th subclone. Each phylogenic tree that we enumerate in this paper is compatible with a set of allocations. Let *k* index each enumerated phylogenic tree, and let ***Q***_*k*_ denote a set of allocations of the *k*th phylogenic tree. For both simulation and real data analysis, we enumerated all phylogenic trees with one to five subclones. In simulation, given a phylogenic tree, each SPM was randomly assigned an allocation with equal probability.

To illustrate, a clonal sample (*S*=1) would have ***Q***_1_=(*q*_11_), where *q*_11_=1 for all SPMs because each SPM is present in all cancer cells. For a sample with two subclones (*S*=2), only one possible tree *A*→*B* exists with a founding subclone *A* and a new subclone *B*. Then, the set of allocations are ***Q***_2_=(***q***_21_,***q***_22_), where $\boldsymbol {q}_{21}^{\mathrm {T}} = (1,1)$ and $\boldsymbol {q}_{22}^{\mathrm {T}} = (0,1)$. The SPMs with allocation ***q***_21_ arise in the founding subclone *A*, and the SPMs with allocation ***q***_22_ arise in the new subclone *B*. For *S*=3, we need to distinguish between linear and branching trees. Let $\boldsymbol {q}_{31}^{\mathrm {T}} = (1,1,1)$, $\boldsymbol {q}_{32}^{\mathrm {T}} = (0,1,1)$, $\boldsymbol {q}_{33}^{\mathrm {T}} = (0,0,1)$, and $\boldsymbol {q}_{34}^{\mathrm {T}} = (0,1,0)$. The linear tree is characterized by ***Q***_3_=(***q***_31_,***q***_32_,***q***_33_), whereas a branching tree is characterized by ***Q***_4_=(***q***_31_,***q***_33_,***q***_34_). (See Additional file 1: Section C.2 for all enumerated configurations based on the list of subclonal assumptions.)

For a clonal SPM located in a region of SCNA, we need to infer its multiplicity, or the number of mutant alleles. If the SPM occurs before the SCNA, its multiplicity is one of the two allele-specific copy numbers of the SCNA; otherwise, its multiplicity is 1. In contrast, based on our assumption that SCNAs are clonal, the multiplicity of a subclonal SPM is always 1. Let *M*_*l*_ be the set of possible multiplicities given the copy number states. Then, *M*_*l*_={*m*|*m*>0 and *m*∈unique(1,*C*_*l*1_,*C*_*l*2_)}, where unique (*Z*) denotes the unique elements of *Z*.

With *S* subclones, let *η*_*s*_ denote the proportion of cells in a tumor sample that belong to subclone *s*, and let ***η***^T^=(*η*_1_,…,*η*_*S*_). Tumor samples derived from bulk tissues are practically never 100% pure, and hence, a proportion of normal cells will contaminate the sample. Let $\phi = \sum _{s=1}^{S} \eta _{s}$ denote a tumor sample’s purity. In addition, write *𝜗*_*s*_=*η*_*s*_/*ϕ* and ***𝜗***^T^=(*𝜗*_1_,…,*𝜗*_*S*_). The vector ***𝜗*** can be interpreted as the set of subclone proportions in the cancer cell population. To characterize ITH within a tumor sample, we utilize the notion of “entropy” or Shannon Index characterized by the expression

$$E = -\sum_{s=1}^{S} \vartheta_{s} \log({\vartheta_{s}}), $$

which corrects for the normal contamination (*ϕ*) because normal cells in the tumor do not contribute to subclonal heterogeneity. This characterization states that more subclones generally lead to a greater degree of ITH and allows for two samples composed of an equal number of subclones to have different degrees of ITH. In addition, the largest possible entropy given *S* subclones is bounded above by log(*S*), corresponding to equal proportions of each subclone (*𝜗*_*s*_=1/*S*).

### Example: allocation, multiplicity, and cellular prevalence

Here, we give a concrete example to explain the notation: allocation, multiplicity, and cellular prevalence. Suppose that a tumor sample is composed of three subclones forming a branching tree: *B*←*A*→*C*. The respective subclone proportions are denoted by *η*_*A*_, *η*_*B*_, and *η*_*C*_. Thus, the sample purity is *ϕ*≡*η*_*A*_+*η*_*B*_+*η*_*C*_, and possible cellular prevalences are (*η*_*A*_+*η*_*B*_+*η*_*C*_)/*ϕ*=1, *η*_*B*_/*ϕ*, and *η*_*C*_/*ϕ*. ***Q***_4_=(***q***_31_,***q***_33_,***q***_34_) characterizes three allocations to consider: ***q***_31_ for clonal mutations; and ***q***_33_ and ***q***_34_ for subclonal mutations that only occur in subclones *B* and *C*, respectively. Suppose that each SPM has one of three copy number states with allele-specific copy numbers being (0,2), (1,1), or (1,3). For SPMs with copy number state (0,2), clonal mutations have multiplicity of 2 if they occur before SCNA and multiplicity of 1 if they occur after SCNA. For SPMs with state (1,1), all mutations (clonal or subclonal) have multiplicity of 1. For SPMs with state (1,3), clonal mutations have multiplicity of 1 or 3 if they occur before the SCNA and multiplicity of 1 if they occur after the SCNA. All combinations of allocation and multiplicity are listed in Table 1.

**Table 1:** Enumerating combinations of allocation and multiplicity for each copy number state

SPM’s CN state	Allocation	Multiplicity	Cellular prevalence
(0,2)	***q***_31_	1	1
	***q***_31_	2	1
	***q***_32_	1	*η*_*B*_/*ϕ*
	***q***_33_	1	*η*_*C*_/*ϕ*
(1,1)	***q***_31_	1	1
	***q***_32_	1	*η*_*B*_/*ϕ*
	***q***_33_	1	*η*_*C*_/*ϕ*
(1,3)	***q***_31_	1	1
	***q***_31_	3	1
	***q***_32_	1	*η*_*B*_/*ϕ*
	***q***_33_	1	*η*_*C*_/*ϕ*

### Modeling SPM read counts

Recall that *A*_*l*_ and *T*_*l*_ denote the alternative read depth and total read depth of the *l*th SPM. For a pre-specified tree structure and copy number estimates, we model *A*_*l*_ given *T*_*l*_ by a mixture of binomial distributions across possible allocations and multiplicities. Next, we provide details to specify such mixture distributions.

We assume that copy number states and tumor purity were estimated by another algorithm, e.g., ASCAT. For the *l*th SPM, denote its copy number state (i.e., allele-specific copy numbers) by *C*_*l*_=(*C*_*l*1_,*C*_*l*2_). Suppose that there are altogether *W* unique copy number states: *c*_1_,..., *c*_*W*_. Given the *w*th copy number state, assume that there are *D*_*w*_ possible combinations of allocation and multiplicity, and denote the *d*th combination by ***e***_*wd*_=(***q***_*d*_,*m*_*wd*_), where ***q***_*d*_ denotes the allocation that depends on the tree structure but not copy number states, and *m*_*wd*_ denote the multiplicity that depends on copy number states. We also allow the estimation of proportion of variants unexplained by combinations of ***U***_*l*_ and *M*_*l*_ following a discrete uniform distribution with proportion parameter denoted *ε*. The mixture proportions of the *D*_*w*_ combinations is denoted by $\phantom {\dot {i}\!}\boldsymbol {\pi }_{w} = (\pi _{w1},\ldots,\pi _{wD_{w}})^{\mathrm {T}}$. Let ***Θ***=(*ε*,***𝜗***,{***π***_*w*_}).

Let ***U***_*l*_ and *M*_*l*_ be the random variables for the latent allocation and multiplicity for the *l*th SPM, respectively, and let ***E***_*l*_=(***U***_*l*_,*M*_*l*_). Write ***G***_*l*_=(*T*_*l*_,*C*_*l*_,*ϕ*,***Θ***). For a single SPM,

$$\begin{array}{@{}rcl@{}} && P \left(A_{l}|\boldsymbol{G}_{l}, C_{l} = c_{w}\right) \\ &=& \epsilon \frac{1}{T_{l}} + (1-\epsilon)\sum_{d=1}^{D_{w}} P \left(\boldsymbol{E}_{l}=\boldsymbol{e}_{wd},A_{l}|\boldsymbol{G}_{l}, C_{l} = c_{w}\right) \\ &=& \epsilon \frac{1}{T_{l}} + (1-\epsilon)\sum_{d=1}^{D_{w}} P \left(\boldsymbol{E}_{l}=\boldsymbol{e}_{wd}|\boldsymbol{G}_{l}, C_{l} = c_{w}\right) \\ && \quad \quad P (A_{l}|\boldsymbol{E}_{l}=\boldsymbol{e}_{wd},\boldsymbol{G}_{l}, C_{l} = c_{w})\\ &=& \epsilon \frac{1}{T_{l}} + (1-\epsilon)\sum_{d=1}^{D_{w}} \pi_{wd} P(A_{l}|\boldsymbol{E}_{l}=\boldsymbol{e}_{wd},\boldsymbol{G}_{l}, C_{l} = c_{w}), \end{array} $$

where

$$A_{l}|\boldsymbol{E}_{l}=\boldsymbol{e}_{wd},\boldsymbol{G}_{l} \sim \text{Binomial}(T_{l},p_{wd}), $$

and $p_{wd} = \frac {m_{wd} \phi \boldsymbol {\vartheta }^{\mathrm {T}} \boldsymbol {q}_{d}}{(C_{l1}+C_{l2}) \phi + 2(1-\phi)}$. In the notation above, $\boldsymbol {\vartheta }^{\mathrm {T}} \boldsymbol {q}_{d} = \sum _{s=1}^{S} \vartheta _{s} q_{ds}$ is the cellular prevalence of a SPM among the tumor’s cancer cells.

Given tumor purity and copy number states, in addition to a particular phylogenetic tree, the likelihood for *L* SPMs is proportional to

$$\prod_{w=1}^{W} \prod_{l: C_{l} = c_{w}} P(A_{l}|\boldsymbol{G}_{l}, C_{l} = c_{w}). $$

Maximization of this likelihood is accomplished by introducing the pair of latent variables (***U***_*l*_,*M*_*l*_), writing the complete-data likelihood, and using an expectation-maximization algorithm, where each iteration of the M-step for ***π***_*w*_ has closed form updating equations, while ***𝜗*** is updated with the quasi-Newton Raphson method Broyden-Fletcher-Goldfarb-Shanno on the expected complete-data log-likelihood conditional on the observed data. In the presence of local optima for this observed mixture likelihood, multiple random initializations of ***𝜗*** are used, while we initialize ***π***_*w*_ by uniform distribution and *ε*=10^−3^.

Inferring the optimal configuration is accomplished using the optimal BIC. Suppose that after running SMASH on *L* SPMs with every enumerated phylogenetic configuration and applying multiple runs of parameter initialization, we arrive at *B* models. For model *b*=1,…,*B*, let *L*_*b*_, *m*_*b*_, *BIC*_*b*_, *S*_*b*_, and *E*_*b*_ denote the log likelihood, model size, BIC, number of subclones, and estimated entropy, respectively, evaluated at the maximum likelihood estimate $\boldsymbol {\widehat {\Theta }}_{b} = (\widehat {{\epsilon }}_{b},\boldsymbol {{\widehat {\vartheta }}}_{b},\boldsymbol {{\widehat {\pi }}}_{b})$. Define *BIC*_*b*_=2*L*_*b*_−*m*_*b*_ log(*L*); models with larger BIC are preferable to models with smaller BIC. We define the posterior probability of model *b* by

$$p_{b} = \frac{\exp\left(0.5 \, BIC_{b}\right)}{\sum_{b^{'}=1}^{B} \exp\left(0.5 \, BIC_{b^{'}}\right)} $$

because BIC provides a large-sample approximation to the log posterior probability associated with the approximating model [25, 26]. Let *p*^∗^= max*b*=1,…,*B*(*p*_*b*_).

It is possible for two or more configurations to have the same BIC. Therefore, we explore two possible definitions of entropy. The first one is a simple average of entropies across all “optimal BIC-decided” models, referred to as “optimally inferred” entropy. The second one is a weighted average of entropies across all models, referred to as “weighted” entropy. These two entropy estimates are

$$E_{o} = \sum_{b=1}^{B} \frac{I \, \, ({p_{b} = p^{*}}) p_{b}}{\sum_{b^{'}=1}^{B} I\,\,({p_{b^{'}}=p^{*}}) p_{b^{'}}} E_{b} $$

and

$$E_{w} = \sum_{b=1}^{B} \frac{p_{b}}{\sum_{b^{'}=1}^{B} p_{b^{'}}} E_{b}. $$

The summation incorporated into *E*_*o*_ accounts for the situation when various configurations or subclone proportions equally fit the observed data.

SMASH is available as an R package integrating Rcpp [27] and RcppArmadillo [28]. The software and source code can be downloaded at https://github.com/Sun-lab/SMASH.

## Results

### Brief overview of SMASH, PyClone, and PhyloWGS

We compared the performance of SMASH versus two popular and representative methods: PyClone [11] and PhyloWGS [9]. PyClone clusters somatic mutations based on their VAFs. From PyClone output (see Additional file 1: Table S1 for an example), one can estimate the number of subclones by the number of mutation clusters. However, to estimate subclone proportions from VAF clusters, we need to know the phylogenetic tree structure (see Additional file 1: Section C.1 for more details). Since PyClone does not estimate a phylogenetic tree, we cannot use PyClone to estimate subclone proportions and thus cannot estimate entropy that is a function of subclone proportions. Unlike PyClone, PhyloWGS was designed to estimate the underlying phylogenetic tree.

SMASH is a frequentist method to infer ITH using a likelihood-based framework. SMASH and PyClone assume each subclone shares the same SCNA profile and that SCNAs and tumor purity have been estimated from an existing algorithm, e.g., ASCAT [5] or ABSOLUTE [29]. Unlike PyClone and PhyloWGS, SMASH explicitly enumerates all possible phylogenetic trees (up to *k* subclones, with default value of *k*=5) and quantifies the likelihood of each tree configuration (refer to the Additional file 1: Section C.2). For each tree configuration, the model parameters are estimated by an EM algorithm that accounts for unobserved somatic mutation allocation across subclones and multiplicity (i.e., copy number of the mutated allele). We can select the optimal phylogenic tree configurations based on the Bayesian information criterion (BIC) and then calculate entropy based on the optimal configuration. Alternatively, to account for the uncertainty of ITH estimation, we can take a weighted summation of ITH entropies, where the weights are the probabilities of different configurations.

### Simulation

To directly compare PyClone and SMASH, we constructed an indicator of high ITH, as done in Morris et al. [12], denoted by *H*, such that *H* = 1 when the number of subclones is greater than *κ*, a predefined integer threshold, and *H* = 0 otherwise. For SMASH, the number of subclones is estimated using the tree configuration with the best BIC. Because PhyloWGS provides estimates of subclone proportions, we can compare the performance of SMASH and PhyloWGS using both entropy and *H*.

#### Setup

To simulate ITH variables, first enumerate the list of tree configurations from one to five subclones, sample the number of subclones denoted *S*. Then, sample among trees with *S* subclones with equal probability.

1. Generate subclone proportions for *S* subclones, denoted as ***η***=(*η*_1_,…,*η*_*S*_)^T^. Simulate ***U***=(*U*_1_,…,*U*_*S*_)^T^, where *U*_*s*_ is simulated from a uniform distribution defined on interval (−3,1). Then calculate $\eta _{s} = \exp ({U_{s}})/[1 + \sum _{s'=1}^{S} \exp ({U_{s'}})]$. Tumor purity is $\phi = \sum _{s=1}^{S} \eta _{s}$, and the subclone proportion for the *s*th subclone is *𝜗*_*s*_=*η*_*s*_/*ϕ*.
2. Calculate entropy $E = -\sum _{s=1}^{S} \vartheta _{s} \log (\vartheta _{s})$, as well as *H*=*I*(*S*>*κ*), where *I* is an indicator function and *κ*=3.

These steps are repeated until the minimum underlying subclone proportion is greater than 0.05, and the minimum difference between the cellular prevalences of two subclones is greater than 0.05 to ensure clusters are separable.

To simulate sequence read counts for the *l*th SPM given a phylogenic tree configuration, we simulated read depth *T*_*l*_ from a negative binomial distribution, sampled copy number state, and then sampled SPM multiplicity and allocation with equal probability. Finally, we generated the number of alternative reads from a binomial distribution. We randomly simulated 5 covariates ***Z***=(*Z*_1_,…,*Z*_5_)^T^ to resemble sex, age, and tumor stage indicators. see Additional file 1: Section A.1 for details.

We simulated the first set of survival times conditional on linear terms ***Z*** and *E* (entropy) and the second set of survival times conditional on linear terms ***Z*** and *H*, both from the Cox proportional hazards model with a constant baseline hazard:

$$\begin{array}{rcl} \lambda(t|E,\boldsymbol{Z}) =& \lambda_{0}(t) \exp\left(\beta_{E} E + \boldsymbol{\gamma}_{Z}^{\mathrm{T}} \boldsymbol{Z}\right), & \text{or} \\ \lambda(t|H,\boldsymbol{Z}) =& \lambda_{0}(t) \exp\left(\beta_{H} H + \boldsymbol{\gamma}_{Z}^{\mathrm{T}} \boldsymbol{Z}\right) &\end{array} $$

where *λ*_0_(*t*)=*λ*_0_= exp(−7.0), *β*_*H*_=*β*_*E*_=0.5, and $\boldsymbol {\gamma }_{Z}^{\mathrm {T}} = (0.55, 0.15, 0.8, 1.7, 2.7)$. Censoring times were simulated from the continuous uniform distribution $\tilde {T} \sim U(0,\tau)$, and the value of *τ* was tuned to generate the desired proportion of censored subjects.

We considered 18 simulation setups, with three censoring rates (20%, 50%, and 70%), three sequencing depths from the negative binomial (parameter values *μ* = 100, 500, and 1000 and *δ* = 2), and two samples sizes (*N*= 400 and 800).

#### Benchmarking

For each ITH method, we applied an extra filtering criterion that each subclone includes at least two mutations that are not part of its parental subclone. PyClone output contains the cellular prevalence for all SPMs, and the SPMs assigned to the same cluster have the same cellular prevalence. Following Morris et al. [12], we removed clusters with only one SPM. Additional file 1: Table S1 provides an example of pre-filtered PyClone output with multiple clusters composed of one SPM. Output of SMASH includes the ITH estimates for each tree configuration (i.e., number of subclones, subclone proportions, and mutations belonging to each subclone) (refer to Additional file 1: Table S2 for a pre-filtered example). We removed configurations where at least one subclone has only one SPM. Similarly, in PhyloWGS, sampled trees with at least one subclone with only one SPM were excluded.

**Fig. 3:**
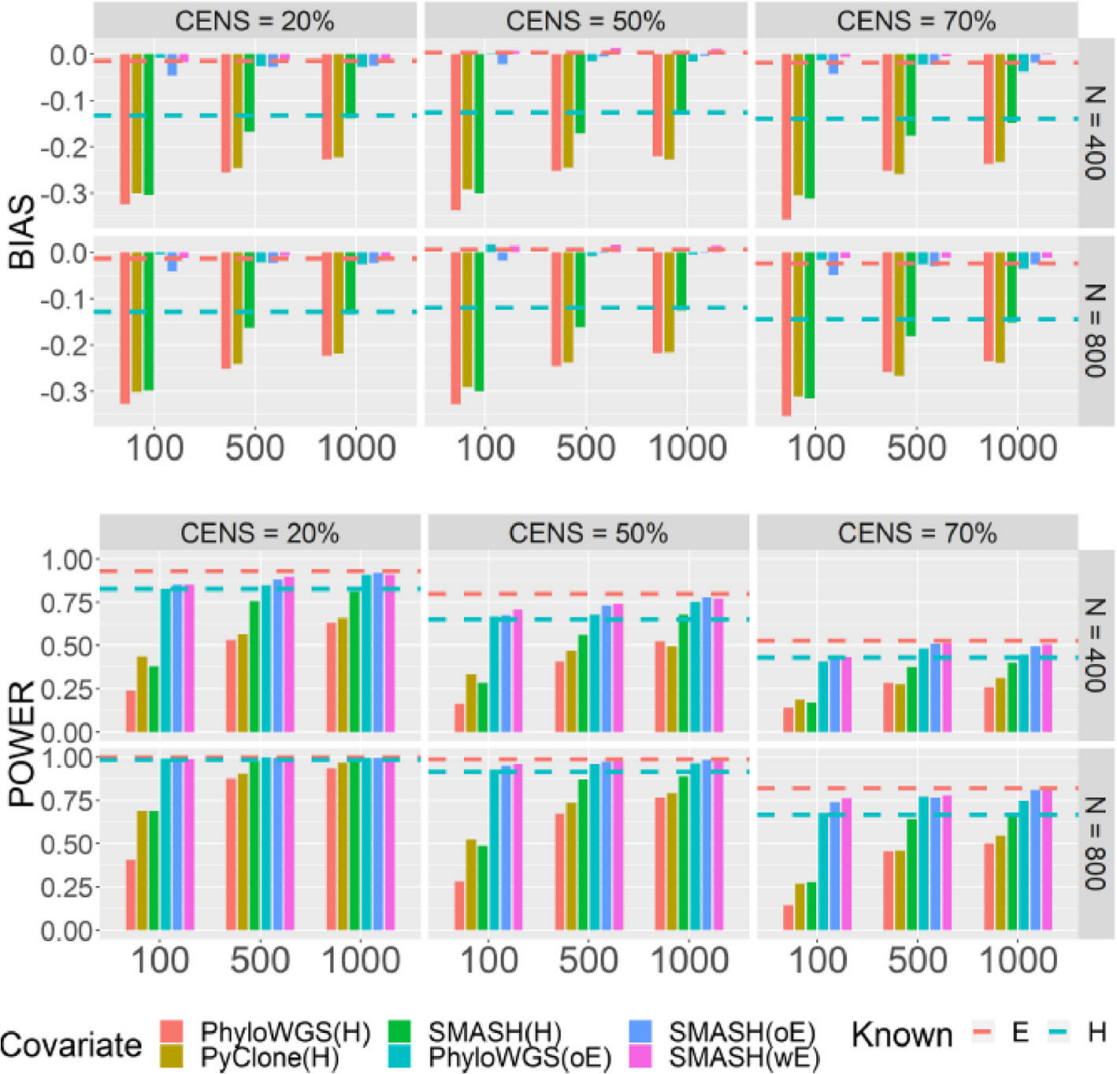
ITH simulation results when the true model contains *E*. The *x*-axis denotes the mean sequencing depth. The *y*-axis denotes the bias of parameter estimates of regression coefficients (*β*_*E*_ or *β*_*H*_) and power at *α*=0.05. Dotted lines denote the bias/power when ITH is known and serve as a benchmark against the estimated ITH metric. *H* is estimated by PhyloWGS (PhyloWGS(H)), PyClone (PyClone(H)), and SMASH (SMASH(H)). *E* is estimated by PhyloWGS’s optimal tree (PhyloWGS(oE)), SMASH’s optimal entropy (SMASH(oE)), and SMASH’s weighted entropy (SMASH(wE))

We used the simulated data to compare the results from five methods: PyClone, PhyloWGS using the optimal tree configuration, SMASH using the configuration with best BIC or weighted summation of entropy/number of subclones, and the ideal situation where true values of entropy or number of subclones are given. Each of the methods was run in two model setups, with the ITH variable being entropy *E* or indicator of high number of subclones *H*. In other words, when the true model contains *E*, we compared the models using *E* or *H*, as shown in Fig. 3. Results for when the true model contains *H* as well as the results for the standard errors of the parameter estimates and coverage probabilities under both models are presented in Additional file 1: Section A.3.

Regardless of the ITH variable used, the bias of parameter estimates remains similar for sample sizes of 400 or 800, and as expected, power increases with sample size (Fig. 3). Given the sample size, bias decreases and power increases as sequence depth increases or censoring rate decreases. Comparing the two ITH metrics, *E* or *H*, the entropy metric has lower bias and higher power. The difference in performance between these two ITH metrics decreases as sequencing depth increases.

**Fig. 4:**
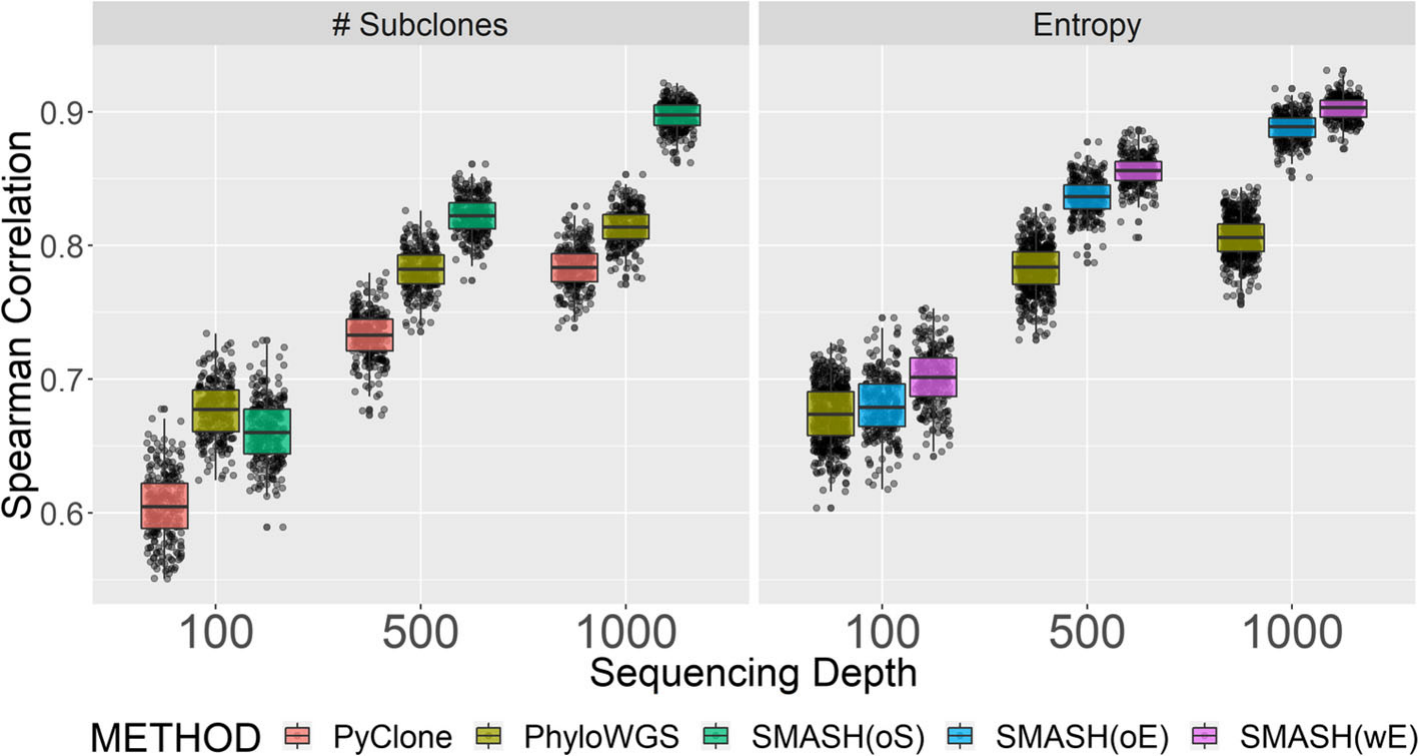
ITH simulation, inferring the optimal number of subclones and entropy. The left plot pertains to Spearman correlations between the true and inferred number of subclones across simulated replicates as a function of sequencing depth and ITH method. The number of subclones are estimated by PyClone (PyClone), PhyloWGS (PhyloWGS), and SMASH using optimal BIC (SMASH(oS)). The right plot pertains to Spearman correlations between the true and estimated entropy using the optimal tree from PhyloWGS (PhyloWGS), optimally inferred entropy from SMASH (SMASH(oE)), and weighted entropy from SMASH (SMASH(wE))

As mentioned, PyClone’s result does not allow us to calculate entropy. Therefore, we compared the performance of PyClone, PhyloWGS, and SMASH using the indicator metric *H*. At an average sequencing depth of 100 ×, SMASH has similar or slightly better performance than PyClone or PhyloWGS, in terms of bias and power. At average depths of 500 × or 1000 ×, SMASH shows much better performance than both PyClone and PhyloWGS (Fig. 3). SMASH demonstrates better performance than PyClone or PhyloWGS when inferring the number of subclones (Fig. 4 and Additional file 1: Figure S3). We calculated the Spearman correlation between the estimated number of subclones and the true number of subclones across 800 samples for each of 250 replicates. The median Spearman correlations from SMASH are consistently higher than those from PyClone and PhyloWGS, except for the comparison with PhyloWGS at read depth 100, in which case PhyloWGS performs slightly better. As read depth increases, the advantage of SMASH against other methods becomes more apparent, which is consistent with their relative performance in association studies (Fig. 3). Comparing PhyloWGS and PyClone, PhyloWGS performs better in terms of capturing the relative order of subclone number, reflected by the Spearman correlation comparison (Fig. 4), but PyClone performs better in terms of estimating the number of subclones (Additional file 1: Figure S3).

When we simulated data using entropy as the ITH metric, as expected, models fit using entropy had higher power and lower bias (Fig. 3). However, even when we simulated the data using *H*, the results using entropy were still better when read depth is low. When read depth is high (e.g., 500 × or 1000 ×), using the estimate of *H* as the ITH variable gives better results, although the difference between using entropy and *H* is often not large (Additional file 1: Figure S2).

Another important comparison is whether weighted entropy, which incorporates uncertainty across all fitted configurations, has better performance than entropy from optimal configurations. Weighted entropy does provide more accurate estimation of true entropy than the optimal entropy (Fig. 4). However, in terms of association estimation, the two approaches have similar performance (Fig. 3). Optimal entropy tends to underestimate the association, while weighted entropy tends to overestimate the association, although the biases are small. In terms of power, both entropies appear to perform equally well. Both weighted and optimal entropies from SMASH are more accurate estimates of the true entropy than the estimate from PhyloWGS’s optimal tree.

In our simulation studies, the vast majority of computational time was spent on ITH inference. On average with 100 mutations, SMASH ran in less than 5 min for ITH inference. In contrast, PyClone and PhyloWGS had run-times ranging from just under 10 min to over 90 min. Additional file 1: Figure S4 presents a summary of computational run-time. Among the three methods with default settings, the order of computational time is SMASH < PyClone < PhyloWGS.

#### Subclonal SCNA simulation

The previous simulation setup assumed SCNAs are clonal. In Additional file 1: Section A.6, we describe the simulation details to allow for subclonal SCNAs. In this analysis, we treated SCNAs as clonal and calculated the copy number by rounding the weighted average of copy numbers across subclones to the nearest integer. As described in Additional file 1: Section A.5, we simulated copy number scenarios 1 and 2 to mimic two patterns of SCNA abundance in real data.

When the true model contains *E*, we compared the results of 6 methods, dichotomized indicator *H* estimated from Pyclone, PhyloWGS, and SMASH, and entropy estimated from PhyloWGS, SMASH with optimal configuration or weighted average (Additional file 1: Figure S8). All three methods using entropy *E* have similar performances and perform much better than the three methods using the dichotomized indicator *H*. Coverage probability was maintained at 95% for *E* estimates but not for *H* estimates. There were no clear differences in performance between both copy number scenarios. When the true model contains *H*, magnitudes of association bias using *E* estimates are generally less than those of *H* estimates (Additional file 1: Figure S9). Therefore, the overall results were consistent with the earlier simulation setup without subclonal SCNAs: using entropy is preferred even if the true model is based on *H*, and entropy from SMASH and PhyloWGS have similar performance at 100 × read depth.

### Application

#### Preprocessing pipeline

We downloaded SPM calls by MuTect2 from NCI’s Genomic Database Commons (GDC) [21, 30]. To derive SCNA data, we processed controlled-access SNP Array 6.0 CEL files corresponding to primary tumors, along with their paired blood-derived normal or solid tissue normal. Specifically, we applied a pipeline involving Birdseed, PennCNV [31], and ASCAT v2.4 [5] to obtain estimates of tumor purity, ploidy, and inferred copy number states. The complete data workflow is shown in Additional file 1: Figure S10. We downloaded SPM and SCNA data on 5898 tumor samples from 14 TCGA cancer types (Additional file 1: Table S3).

Before running PyClone, PhyloWGS, and SMASH, we applied a set of filters to the SPM data by retaining the base substitution SPMs that are located along autosomes and have at least seven reads supporting the alternative allele. Also, those SPMS with inferred total copy number of zero were excluded. Then, we passed the formatted SPM and SCNA data to PyClone, PhyloWGS, and SMASH for ITH inference. After running all three ITH methods, we applied the “at least two mutations per subclone/cluster” criterion that was used in the simulation.

#### Somatic mutation landscape varies across cancer types

**Fig. 5:**
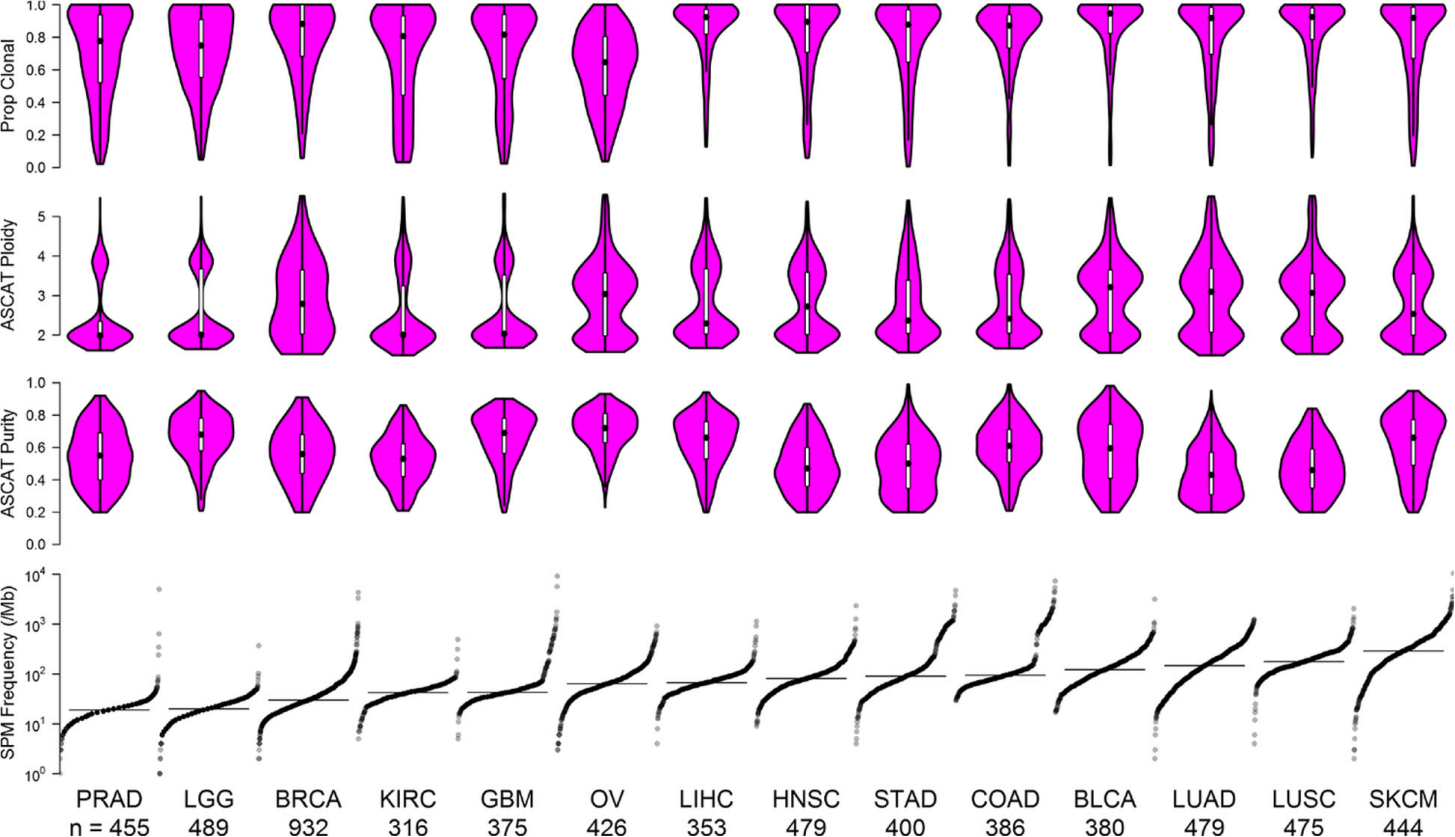
Mutation rate, purity, ploidy, and proportion of clonal mutation summary: mutation load per megabase within the whole exome and ASCAT-derived purity and ploidy across 14 cancer types ordered by a median mutation rate. Violin plots in magenta contain nested boxplots with the median represented by the black box. The top panel shows the distribution of the proportion of inferred clonal mutations across all samples for each cancer type. The cancer types are bladder urothelial carcinoma (BLCA), breast invasive carcinoma (BRCA), colon adenocarcinoma (COAD), glioblastoma multiforme (GBM), head/neck squamous cell carcinoma (HNSC), kidney renal clear cell carcinoma (KIRC), lower-grade glioma (LGG), liver hepatocellular carcinoma(LIHC), lung adenocarcinoma (LUAD), lung squamous cell carcinoma (LUSC), ovarian serous cystadenocarcinoma (OV), prostate adenocarcinoma (PRAD), skin cutaneous melanoma (SKCM), and stomach adenocarcinoma (STAD)

We first summarized tumor purity, ploidy, and somatic mutation rate for each tumor type (Fig. 5). The relative ordering of tumor types by mutation rate is consistent with the results reported in an earlier study [32]. Those cancer types with lower mutation rate (e.g., PRAD, LGG, BRCA, KIRC, GBM, and OV) tend to have more subclonal mutations (top panel of Fig. 5). In all cancer types except OV, more than 50% of somatic mutations are clonal (with cellular prevalence larger than 99%) (Additional file 1: Figure S19). Ovarian cancer appeared to be an outlier with the larger number of subclones. This may be partly due to batch effects. The ovarian cancer samples used whole genome amplification (WGA) before DNA sequencing that may have reduced the quality of DNA samples [33, 34]. On the other hand, some previous work did show a high level of ITH in ovarian cancers [35–37]. Blagden [36] mentioned that the phylogenetic tree of ovarian cancer “has a short trunk and many branches, representing early clonal expansion and high genomic instability.” This was consistent with our finding that ovarian cancer has higher levels of ITH. The ploidy values of most cancer types tended to cluster around 2 and 4 (genome-wide duplication). This clustering pattern was less clear for BRCA, suggesting a greater degree of SCNA in BRCA.

We examined the cellular prevalence of 49 genes that are among the top 10 mutated genes for at least one of the 14 cancer types (Additional file 1: Figure S21). Similar to our approach to calculate weighted entropy (refer to the “Methods” section), each mutation’s cellular prevalence was calculated as the weighted average across the sample’s ITH configurations. A gene’s cellular prevalence was calculated as the average cellular prevalence of all mutations on that gene across all samples. TP53 mutations have average cellular prevalences near 1.0 for all cancer types except KIRC, which was the same observation made by Morris et al. [12]. IDH1 mutations were subclonal in GBM and clonal in LGG and SKCM. VHL was uniquely called in KIRC, with a cellular prevalence of 1.0. Except for TP53, the remaining 48 genes have relatively low cellular frequency in OV. This was consistent with the results of an earlier study of 31 ovarian tumor samples from six patients, and they found TP53 was the only gene mutated in all samples, and other known tumor driver genes may be mutated in some but not all samples of a patient [38]. Hierarchical clustering was performed on the 49 genes and 14 cancer types. At least two clusters of cancer types and at least two clusters of genes were apparent. LGG, KIRC, and PRAD form one cluster of cancer types without many mutations on these 49 genes.

The number of subclones by tumor type and ITH method are summarized in Additional file 1: Figure S14. Across all cancers, SMASH consistently identified more subclones than PyClone. Between SMASH and PhyloWGS, the resulting number of subclones was very similar for all tumor types except for OV. PyClone was run on two independent Markov chains on each tumor sample using its default setup with 20,000 MCMC samples drawn, 1000 burnin and retaining every tenth sample with all default prior hyperparameters. PhyloWGS also was run twice but with default arguments. There were slight inconsistencies from the results of the two runs (Additional file 1: Tables S4 and S5). In the next section on association analysis, we used the first run of results from PyClone and PhyloWGS.

#### Baseline covariates and variable selection

The common set of baseline covariates included age at diagnosis, gender, pathological tumor stage, tumor mutation burden (total number of point mutations, TMB), and genome-wide SCNA burden. Specifically, we define genome-wide SCNA burden as

$$\sum_{k} \frac{L_{k}}{\sum_{k^{'}} L_{k^{'}}} \left[\left|C^{A}_{k} - 1\right| + \left|C^{B}_{k} - 1\right|\right], $$

where *k* indexes genome segments, *L*_*k*_ is the length of the *k*th segment, and $(C^{A}_{k},C^{B}_{k})$ are the segmental clonal copy numbers of the minor and major alleles, respectively. The SCNA burden can be interpreted as the distance between the normal and cancer genomes, in terms of copy number. Both TMB and SCNA burden were binned into three equal groups using the 33rd and 66th quantiles as cutoffs.

We investigated possible non-linear forms of entropy (e.g., dichotomized entropy, polynomial transformation, or log transformation) and the validity of the proportional hazard assumption using R functions fcov() and prop() from R package goftte [39, 40]. Our analysis suggested that the simple linear form of entropy is appropriate. Since our simulation studies showed that the weighted entropy provides better estimates of the true entropy than the optimal entropy (Fig. 4), we chose to conduct the following analysis using weighted entropy.

In addition to baseline covariates, additional covariates to include in each tumor type’s full model were carefully selected. The top four frequently mutated genes were included. Other tumor type-specific covariates were histological subtype for BLCA (papillary vs. non-papillary), PAM50 subtype for BRCA [41] (Basal, Her2, LumA, or LumB, and the normal-like subtype was removed due to its small sample size), tumor grade for KIRC, IDH/CNA status for LGG (IDH wild-type, IDH mutant without chr1p and 19q co-deletion, IDH mutant with chr1p and 19q co-deletion), and Gleason score and PSA level for PRAD.

We also considered the pairwise interactions of all baseline covariates with weighted entropy. The final model for each tumor type was selected based on step-wise model fitting and assessed with Akaike information criterion (AIC). When the final model contained pairwise interactions involving entropy, then the interactions were retained if their minimum *p* value was less than or equal to 0.02. Otherwise, the interaction was removed, and our variable selection was re-run without the interaction term. When the final model excluded entropy, it was added back in the final step.

#### TMB and ITH are associated with survival time in multiple cancer types

In the PRAD cohort, because very few deaths were observed, we only analyzed progression-free survival (PFS). For all other cancer types, we studied both overall survival (OS) and PFS. We used a *p* value cutoff of 0.05 to define statistical significance.

**Fig. 6:**
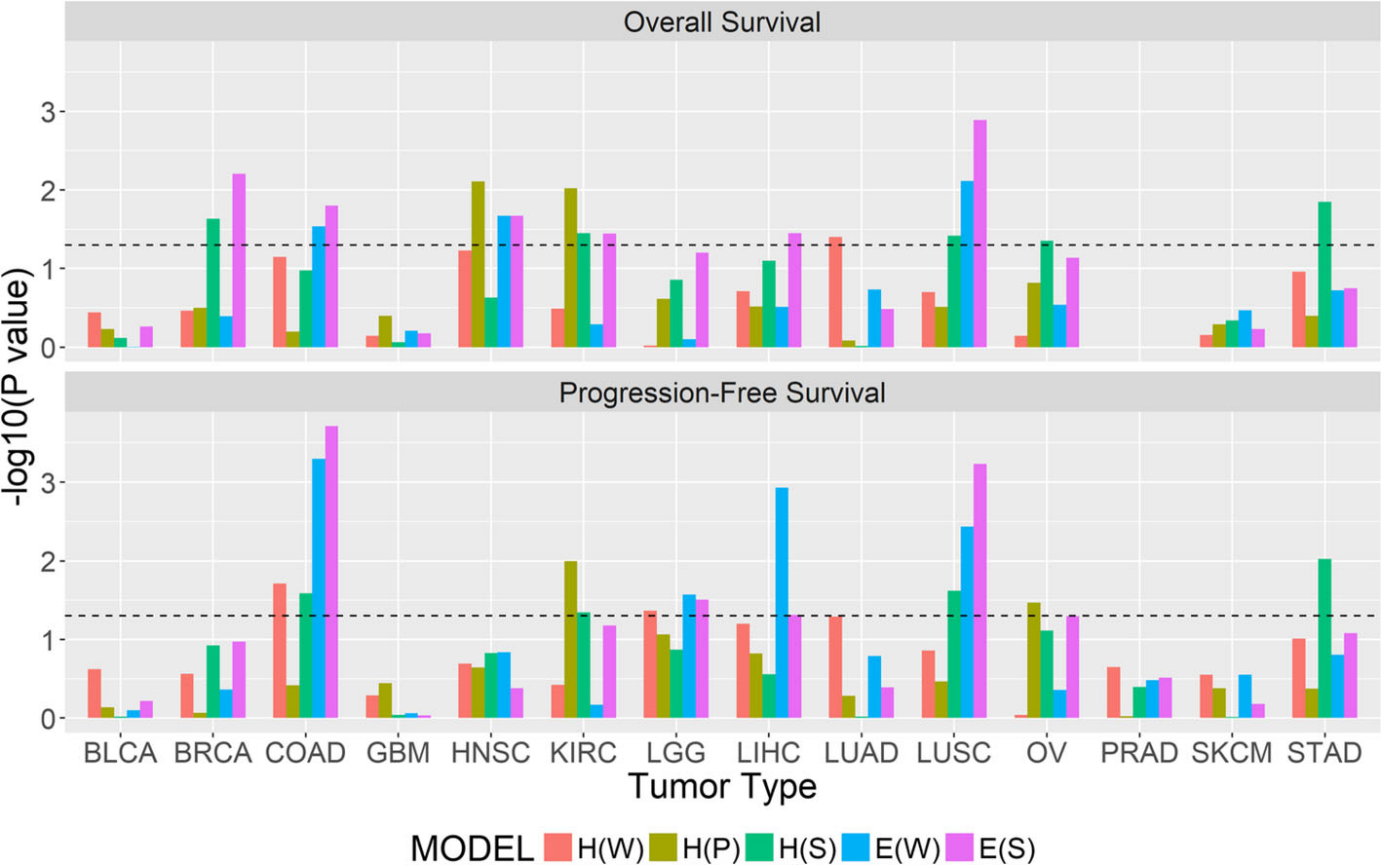
Comparing *p* values of all the ITH-related variables across tumor types. For each cancer type, we assessed the association between ITH and survival time by comparing the final model to the reduced model obtained by excluding all ITH-related variables. The horizontal line indicates the *p* value cutoff 0.05. H(W), H(P), H(S) denote the indicator for three or more subclones from PhyloWGS, PyClone, and SMASH, respectively. E(W) and E(S) denote entropy from PhyloWGS and SMASH, respectively

For OS, entropy or its interaction with other variables were statistically significant in the final model for 6 of 14 cancer types: BRCA, COAD, HNSC, KIRC, LIHC, LUSC (Fig. 6). Total mutation burden (TMB) was statistically significant for 7 cancer types: BLCA, COAD, GBM, LGG, LUAD, OV, and STAD (Additional file 1: Figure S17). SCNA burden (SCNAB) was statistically significant for LGG and SKCM (Additional file 1: Table S6–S19). Significant associations between gene-level mutation status and OS include TP53 for BLCA, GBM, HNSC, LIHC, LUSC and STAD, TTN for GBM and LUSC, and MUC16 for SKCM (Additional file 1: Table S6–S19).

In addition to these somatic mutation-based predictors, age at diagnosis was statistically significant for all tumor types except LIHC and LUAD. Sex was statistically significant for GBM, HNSC, and LIHC. All GBM tumors are stage IV. Among all other cancer types, tumor stage was associated with overall survival except for LGG and OV. Other tumor type-specific covariates associated with OS include PAM50 for BRCA, tumor grade for KIRC, and IDH/CNV status for LGG (Additional file 1: Table S6–S19).

The model fits for PFS were similar to the ones for OS for most cancer types. For GBM, KIRC, LUSC, OV, SKCM, and STAD, the final model for PFS was the same as the final model for OS survival. Covariates present in one model but not in the other model were highlighted in Additional file 1: Table S6–S19.

We also reported the results when replacing SMASH’s weighted entropy (E(S)) with PhyloWGS’s entropy (E(W)), the dichotomized number of subclones from SMASH (H(S)), PyClone (H(P)), and PhyloWGS (H(W)) (Fig. 6 and Additional file 1: Table S6–S19). H(S), H(P), and H(W) were constructed as indicators of 3 or more subclones. This cutoff was chosen so that there were enough samples with non-censored survival time in the high ITH group. Overall, the associations we detected by H(S), H(P), or H(W) were consistent with the results by E(S) and E(W), and the *p* values by E(S) tended to be smaller. An exception was in STAD, where H(S) identified significant associations for both OS and PFS that were missed by H(P), H(W), E(W), and E(S).

Our results bring new insights that have not been reported by previous studies [1, 12]. Andor et al. [1] studied 1165 samples of 12 cancer types. They found significant association between ITH (the number of subclones) and survival time in only one cancer type: gliomas (combining two types of cancer from LGG and GBM). Morris et al. [12] studied 3300 tumor samples in 9 cancer types. They used dichotomized number of subclones as ITH measurement (# of subclone >4 for most cancer types), which is very unstable because few samples had more than 4 subclones. They found significant associations between ITH and survival time in 5 out of 9 cancer types: BRCA, HNSC, KIRC, LGG, and PRAD. They also added mutation burden into the Cox model for these five cancer types and found mutation burden was not significant in all five cancer types. We have 5898 TCGA tumor samples from 14 cancer types. We considered both dichotomized number of subclones and entropy as measurements of ITH. While Morris et al. did not find mutation burden to be informative for prognosis, we found it is significantly associated with survival time (or marginally significant) in 7 of the 14 cancer types. What is truly new in our findings is that we consider both ITH measurement and its interaction with other covariates, such as mutation burden, tumor stage, and mutation status of a particular gene. We found a considerable amount of heterogeneity for the results across cancer types.

## Discussion

### Quantification of ITH

We considered two ITH metrics: entropy and indicator for high number of subclones. When we simulated survival time given entropy, as expected, using entropy instead of the indicator as the ITH metric led to better performance in association analysis (Fig. 3). Interestingly, when we simulated survival time given the indicator, the model with entropy has either higher power (when read depth is 100) or comparable power (when read depth is 500 or 1000) (Additional file 1: Figure S2). In real data analysis, using entropy as the ITH metric also led to more discoveries. Therefore, we recommend using entropy as an ITH metric in association studies. One reason for entropy delivering better results is that, as a continuous variable, entropy is more robust to noise in ITH inference. Specifically, the addition or deletion of a subclone with small cellular proportion may change entropy slightly but may change the indicator variable from 0 to 1. In addition, some information about the degree of ITH is lost when dichotomizing the number of subclones. Of course, an intermediate choice is to use the number of subclones. As shown in Additional file 1: Figure S13, entropy was highly associated with the number of subclones and provides a more refined quantification for samples with the same number of subclones.

Another question that we sought to answer was whether it was beneficial to incorporate the uncertainty of ITH inference in association analysis. Towards this end, we studied two versions of entropy from SMASH, the optimal entropy derived from the mean entropy of the tree configurations with optimal BIC versus the weighted entropy across all estimated tree configurations. The weighted entropy has slightly higher correlation with the true entropy than with the optimal entropy, although these two quantities have similar power to detect associations.

### Study design for future ITH studies

Our simulation results suggested that when using entropy as the ITH metric, more power was gained by increasing the sample size from 400 to 800 than by increasing the read depth from 100 to 500 or even 1000 (Fig. 3, Additional file 1: Figure S2). In contrast, when using the indicator *H* as an ITH metric, increasing read depth can also bring some relatively large power gains (Fig. 3, Additional file 1: Figure S2). One issue that warrants future study is the benefit of having multiple tumor samples per patient.

ITH measurement may be affected by somatic mutation calling accuracy. A previous study [42] showed that the sensitivity of somatic mutation calling is around 0.8–0.9, and the number of false positive mutation calls is around 30 mutations for the whole exome using mutation callers such as Strelka or Mutect. We can further reduce the number of false positives by taking the intersection of mutation calls from multiple callers, with the trade off to reduce sensitivity of mutation calls. Our method is robust to low sensitivity of mutation calls because we use cellular frequency of subclones to estimate entropy, and if, for example, 6 of 10 mutations of a subclone are called, we can still use these 6 mutations to estimate subclone cellular frequency. Therefore, if one suspects a high proportion of false positive mutation calls, one strategy is to restrict the analysis to the mutations called by more than one caller.

### Association between survival time and ITH or TMB

In most cancer types, when TMB is included in the final model, it is negatively associated with hazard, and thus higher mutation burden leads to longer survival time (Additional file 1: Figure S18). This may be explained by the observation that tumors with higher TMB are more likely recognized and attacked by the immune system [43]. However, higher TMB is associated with worse survival time in LGG.

TMB is positively associated with entropy measurement of ITH, although the correlation is not strong enough to create any concerns with co-linearity when using both variables in a model (Additional file 1: Figure S16). We also observed interactions between TMB and ITH for both OS and PFS in COAD and LUSC. In both cases, association between survival time and entropy is not significant when TMB is low. However, higher entropy is associated with worse survival time when TMB is high. In LUSC, we also observed interaction between entropy and TP53 mutation. When TP53 is mutated, higher entropy is associated with longer survival time for both OS and PFS (Additional file 1: Figure S20). These results suggest that the effect of ITH on survival time may depend on other factors.

### Limitations

Our analyses have some limitations. One limitation is the assumption of clonal SCNA. Employing this assumption allows us to use copy number calls from mature and widely used methods such as ASCAT or ABSOLUTE and to maintain high computational efficiency. However, this assumption also risks classifying SPMs in subclonal SCNA regions as SPMs from a new subclone. This risk may not bias the entropy estimate because a new subclone with subclonal SCNA is captured by SPMs. As shown in two simulation settings with subclonal copy number, SMASH has similar performance as PhyloWGS’s when there are high levels of subclonal SCNAs.

Another limitation, shared by all methods for inferring ITH from SPMs, is that we cannot distinguish two subclones whose somatic mutations have very similar cellular prevalence. For example, in Fig. 1, the mutations from subclones A and B have very similar cellular prevalence and hence cannot be distinguished. However, this is a limitation of the input data rather than the methodology. This limitation can be overcome if multiple samples per patient are available.

The infinite site assumption may be considered too strong an assumption. One study demonstrated possible evidence of recurrent mutations in their single-cell sequencing data [44]. Conceptually, if mutations were recurrent, somatic mutations from bulk sequencing could not be utilized for modeling multiplicity and somatic inheritance among subclones. Therefore, ITH inference and association analyses could only be conducted with single-cell sequencing to better infer cellular multiplicities. Though, if only a handful of mutations were recurrent and at a small fraction of cells, their inferred cellular prevalence may slightly decrease relative to an identical non-recurrent mutation, leading to a biased subclone proportion estimate. This entropy estimate could be treated as being an extra “noisy” estimate. But as long as this biased estimate correlates with the underlying entropy, there may still be power to detect the association between entropy and clinical outcomes.

## Conclusions

We have conducted a pan-cancer analysis to study the associations between somatic mutations and survival time in 14 cancer types. Several types of somatic mutation features are included in our analysis, including mutation burden, copy number alteration burden, mutation status of a few frequently mutated genes, and intra-tumor heterogeneity (ITH) inferred by our method SMASH. We conclude that using entropy instead of high ITH indicator as the ITH metric leads to higher power in association analysis. The effect of ITH may depend on other somatic mutation features such as mutation burden. Accounting for the uncertainty of ITH inference has some but limited benefit. To improve the power for association analysis, it is much more effective to increase the sample size than generating more reads per sample.

## Additional file

Additional file 1 Supplementary results and methods, including Additional file 1: TableS1-S19 and Additional file 1: FigS1-S21. (PDF 3184 KB)

## Abbreviations

ASCAT: Allele-specific copy number analysis of tumors
ITH: Intra-tumor heterogeneity
MATH: Mutant-allele tumor heterogeneity
SCNA: Somatic copy number alterations
SMASH: Subclone multiplicity allocation and somatic heterogeneity
SPMs: Somatic point mutations
TCGA: The Cancer Genome Atlas
TMB: Tumor mutation burden
VAF: Variant allele frequencies
