# Supplementary material for: Associating somatic mutations to clinical outcomes: a pan-cancer study of survival time
Source: Genome Med. 2019 May 28;11:37. doi: 10.1186/s13073-019-0643-9 (PMC6540540; doi:10.1186/s13073-019-0643-9)
Supplement: Supplementary file 1 — Supplementary results and methods, including Additional file 1: TableS1-S19 and Additional file 1: FigS1-S21. (PDF 3184 KB) [file 13073_2019_643_MOESM1_ESM.pdf]

Supplementary Materials for "Associating Somatic Mutations to Clinical Outcomes: a Pan-cancer Study of Survival Time"

Table of Content

A Supplementary Results for Simulation Studies **5**
A.1 Additional details for simulation setup 5
A.2 Supplementary simulation example of PyClone and SMASH output 6
A.3 Additional simulation results 7
A.4 Computational Runtime 10
A.5 Proportions of genome with SCNAs and subclonal SCNAs 11
A.6 Simulation setup with subclonal copy number altered somatic mutations 13
A.7 Subclonal SCNA results 14

B Supplementary Results of Pan-cancer Analysis **17**
B.1 Workflow 17
B.2 Sample size summary across cancer types 17
B.3 Additional results of ITH across cancer types. 18
B.4 Cancer type-specific analysis results 29

C Supplementary Methods **33**
C.1 An example for the ambiguity to estimate subclone proportions based on mutation VAFs 33
C.2 A list of subclone configurations 33

List of Tables

S1 **Example of PyClone cellular prevalence cluster results.** "cluster_id", "size", "mean", and "std" denote cluster number, number of SPMs belonging to the cluster, cluster mean cellular prevalence, and cluster mean's standard deviation, respectively. 6

S2 **SMASH Cluster Results.** The table provides a subclone configuration overview of all feasible configurations given the inputted SPMs, SCNAs, and tumor purity. "cc" denotes the number of subclones. Given "cc", "kk" denotes the configuration index. "ms" denotes the model size, i.e., the number of parameters estimated. "LL" denotes the log likelihood evaluated at maximum likelihood estimates. "q" denotes the subclone proportions among cancer cells, $\boldsymbol{\vartheta}$, described in the main text. "alloc" denotes the inferred number of mutations that emerge in each subclone. 6

S3 Summary of different TCGA tumors studied in our preliminary analysis. 17

S4 **Summary of PyClone reproducibility.** Assessing reproducibility of PyClone by running two fixed seeds with the same tumor sample input data run with 20,000 draws, 1,000 burn in and retaining every tenth sample drawn. Cellular prevalence clusters with one SPM were excluded before inferring the number of subclones. 27

S5 **Summary of PhyloWGS reproducibility.** Assessing reproducibility of PhyloWGS by running the algorithm twice for each sample with default arguments but different seeds. . 28

S6 Bladder urothelial carcinoma 29

S7 Breast invasive carcinoma 29

S8 Colon adenocarcinoma 30

S9 Glioblastoma multiforme 30

S10 Head/neck squamous cell carcinoma 30

S11 Kidney renal clear cell carcinoma 30

S12 Lower grade glioma 31

S13 Liver hepatocellular carcinoma 31

S14 Lung adenocarcinoma 31

S15 Lung squamous cell carcinoma 31

S16 Ovarian serous cystadenocarcinoma 32

S17 Prostate adenocarcinoma 32

S18 Skin cutaneous melanoma 32

S19 Stomach adenocarcinoma 32

List of Figures

S1 **ITH simulation results when the true model contains** E. The x-axis denotes the mean sequencing depth. The y-axis denotes the standard error of the parameter estimator (SE) or the coverage probability (CP) of the 95% confidence interval. Dotted lines denote the bias/power when the ITH variable is known and serve as a benchmark against the estimated ITH variable. H is estimated by PhyloWGS (PhyloWGS(H)), PyClone (PyClone(H)), and SMASH (SMASH(H)). E is estimated by PhyloWGS's optimal tree (PhyloWGS(oE)), SMASH's optimal entropy (SMASH(oE)), and SMASH's weighted entropy (SMASH(wE)) 7

S2 **ITH simulation results when the true model contains** H**.** The x-axis denotes the mean sequencing depth. The y-axis denotes the bias of parameter estimate of regression coefficients (β_E or β_H), power, standard error of the parameter estimator (SE), and coverage probability (CP) of the 95% confidence interval. Dotted lines denote the bias/power when the ITH variable is known and serve as a benchmark against the estimated ITH variable. 8

S3 **Comparing the estimated and true ITH measures**. Each point represents the calculated proportion of samples from a total of 800, where the estimated number of subclones or dichotomized number of subclones matches the truth. 9

S4 Computational runtime is plotted against mutation burden and ITH method. 10

S5 **Comparing the distributions of samples' genome proportions of copy number alterations by tumor type.** 11

S6 **Comparing the distributions of samples' genome proportions of subclonal copy number alterations (SubCNA) by tumor type.** 12

S7 Simulated distributions of proportion of the genome with CNA (p_c) and proportion of the genome with subclonal CNA (p_s) by the two proposed scenarios. The blue line corresponds to the default estimated density. 14

S8 **ITH simulation when the true model contains** E 15

S9 **ITH simulation when the true model contains** H 16

S10 Real Data Pipeline/Workflow containing the SCNA workflow in blue nodes and the SPM workflow in red nodes. The combined SPM/SCNA dataset is represented by the green node. The SPM/SCNA dataset is then passed on to ITH methods PyClone, PhyloWGS, and SMASH. 17

S11 Histograms of tumor VAF of all clustered somatic mutations by tumor type. 18

S12 Histograms of log10 transformed total read depth of all clustered somatic mutations by tumor type. 19

S13 **SMASH entropy vs. number of subclones by tumor type:** imputed mean entropy on the y-axis, and number of subclones on the x-axis. Percentages represent the distribution of the number of subclones within each cohort. Horizontal jitter was added to avoid excessive over-plotting. We estimated the number of subclones using the number of subclones associated with the optimal configuration(s). 20

S14 **Comparing the distribution of estimated number of subclones across ITH methods PhyloWGS, PyClone, and SMASH.** . . . 21
S15 **Comparing the distribution of entropy between PhyloWGS and SMASH by tumor type.** Violin plots with nested boxplots are plotted. . . . 21
S16 **Correlation between entropy and TMB.** Spearman correlation was calculated between the log10(TMB+1) and weighted entropy and displayed within each plot. . . . 22
S17 **Comparing the association between TMB and survival outcome across tumor types.** The -$\log_{10}$(p-value) was plotted for cancer types in which TMB was retained in the final model. The p-value is calculated by a likelihood ratio test comparing the final model and the reduced model without any TMB-related terms. . . . 22
S18 **Comparing the association between TMB and survival outcome across tumor types using the log hazard ratio.** The y-axis denotes estimated differences in log hazards ratio between TMB bin 3 and TMB bin 1 with all other variables set to their reference group or zero. These differences are shown for cancer types in which TMB was retained in the final model. . . . 23
S19 **Proportion of binned mutational cellular prevalences by tumor type as inferred by SMASH.** . . . 24
S20 **Studying the predicted survival probability from LUSC tumors.** The Cox model for overall survival contains wE, TP53 mutation status, TMB, and interactions between wE and TP53 and wE and TMB. wE denotes weighted entropy. TMB=1 and TMB=3 denote total mutation burdens of 4-139 SPMs and 228-2044 SPMs, respectively. TP53=WT and TP53=MT denote wild-type and mutated TP53 gene, respectively. Comparing the predicted survival for an individual with wE=0 versus wE=1 (approximately clonal versus two-subclone sample), TMB=1 versus TMB=3, and TP53 wild-type (WT) versus mutated (MT) gene status. . . . 25
S21 **Cellular prevalence heatmap.** Each cell of this heatmap matrix represents the mean cellular prevalence of SPMs within a gene and tumor type. A gene is considered if it is mutated in at least 10 subjects of one tumor type. The gene list was generated from the top ten frequently mutated genes across samples for each tumor type. Mutations with cellular prevalence between 0.08 and 0.5 are excluded from the heatmap. Genes and tumors were hierarchically clustered with default arguments for R's `hclust()` function. Gray matrix cells indicate a lack of point mutations, after our filtering criteria were applied. . . . 26

A Supplementary Results for Simulation Studies

A.1 Additional details for simulation setup

To simulate sequence read counts for the lth SPM given a phylogenic tree configuration:

1. Simulate total depth $T_l \sim NB(\mu, \delta) + 30$, where $NB(\mu, \delta)$ denotes a negative binomial distribution with mean μ and over-dispersion parameter δ. The additional constant 30 is added as an lower bound of the total depth.

2. Sample copy number state (C_{l1}, C_{l2}) from a discrete bivariate distribution such that $C_{l1} \leq C_{l2}$ and $C_{l1} + C_{l2} \leq 5$.

3. Sample SPM multiplicity and allocation with equal probability. Then, generate the number of alternative reads from a binomial distribution (see Supplementary Materials Section A.1 for details).

We considered copy number states harboring SPMs with total copy number ranging from one to five and thus defined the set of copy number states considered by

$$\mathcal{C} = \{(c_1, c_2) : c_1 \leq c_2, 0 < c_1 + c_2 \leq 5\},$$

where c_1 and c_2 are integers. For a given subject, let B denote the number of copy number states considered. First, we drew B from the set of the numbers 1 through 5 with equal probability. Given B, we let the probability of sampling state (c_1, c_2) equal

$$P((c_1, c_2)|B) = \begin{cases} \dfrac{p^{|c_1-1|+|c_2-1|}}{\sum_{(c_1^*, c_2^*) \in \mathcal{C}: c_1^* = c_2^*} p^{|c_1^*-1|+|c_2^*-1|}} & \text{if } B = 1 \\ \dfrac{p^{|c_1-1|+|c_2-1|}}{\sum_{(c_1^*, c_2^*) \in \mathcal{C}} p^{|c_1^*-1|+|c_2^*-1|}} & \text{if } B > 1 \end{cases}.$$

Because $p^{|c_1-1|+|c_2-1|}$ achieves its largest value when $c_1 = 1$ and $c_2 = 1$, this simulation setup encourages the diploid state with copy number $(1, 1)$. In the simulations, we set $p = 0.55$.

Next, we sampled multiplicity m and allocation $\boldsymbol{q}_{u\cdot}$ with equal probability. Here, $\boldsymbol{q}_{u\cdot}$ is a vector of length S, and its sth element is 1 if the mutation is observed in the sth subclone and 0 otherwise. Then, we generated the number of alternative reads $A_l \sim Bin(T_l, p_l)$, where $Bin(T_l, p_l)$ is a binomial distribution with success probability $p_l = \dfrac{m\boldsymbol{\eta}^{\mathrm{T}}\boldsymbol{q}_{u\cdot}}{(C_{l1} + C_{l2})\phi + 2(1-\phi)}$.

We also randomly simulated 5 covariates $\boldsymbol{Z} = (Z_1, \ldots, Z_5)^{\mathrm{T}}$ to resemble sex (Z_1), age (Z_2), and tumor stage indicators for stage 2, 3, and 4 (Z_3, Z_4, and Z_5), respectively, where Z_1 is Bernoulli with success probability 0.5, $\tilde{Z}_2$ is a simple random resampling of integers between 50 and 90 and then centered and scaled to have unit variance, and $\tilde{Z}_3$ is simple random resampling of integers between 1 and 4, and $Z_j = I\left(\tilde{Z}_3 = j - 1\right)$ for $j = 3, 4, 5$.

A.2 Supplementary simulation example of PyClone and SMASH output

Table S1 **Example of PyClone cellular prevalence cluster results.** "cluster_id", "size", "mean", and "std" denote cluster number, number of SPMs belonging to the cluster, cluster mean cellular prevalence, and cluster mean's standard deviation, respectively.

cluster_id	size	mean	std
1	25	0.988486668245	0.0128057112343
2	1	0.721257882342	0.157567289382
3	1	0.722335130682	0.130356295273
4	1	0.736048387235	0.105942318136
5	59	0.426215614324	0.0129755499243
6	1	0.67081552269	0.176334569783
7	1	0.67081552269	0.176334569783
8	1	0.300085071822	0.14397170448
9	32	0.103118729188	0.0088194636314
10	1	0.246008370273	0.104567924163
11	1	0.247258883914	0.086684170178

Table S2 **SMASH Cluster Results.** The table provides a subclone configuration overview of all feasible configurations given the inputted SPMs, SCNAs, and tumor purity. "cc" denotes the number of subclones. Given "cc", "kk" denotes the configuration index. "ms" denotes the model size, i.e., the number of parameters estimated. "LL" denotes the log likelihood evaluated at maximum likelihood estimates. "q" denotes the subclone proportions among cancer cells, $\boldsymbol{\vartheta}$, described in the main text. "alloc" denotes the inferred number of mutations that emerge in each subclone.

cc	kk	ms	entropy	LL	AIC	BIC	q	alloc
1	1	0	0.00	−30832.96	−61665.92	−61665.92	1	1|126
2	1	3	0.62	−23860.06	−47726.12	−47734.63	0.68,0.32	1|27;2|99
3	1	6	0.92	−22985.64	−45983.28	−46000.3	0.56,0.34,0.1	1|25;2|67;3|34
3	2	6	0.94	−22985.64	−45983.28	−46000.3	0.47,0.1,0.44	1|25;2|34;3|67
4	1	8	1.16	−22972.51	−45961.02	−45983.71	0.5,0.1,0.31,0.1	1|25;2|23;3|44;4|34
4	1	9	1.18	−22958.23	−45934.46	−45959.99	0.5,0.12,0.28,0.1	1|25;2|36;3|31;4|34
4	2	9	1.03	−22958.23	−45934.46	−45959.99	0.5,0.03,0.38,0.1	1|25;2|36;3|31;4|34
4	3	9	1.21	−22958.23	−45934.46	−45959.99	0.41,0.12,0.38,0.1	1|25;2|36;3|31;4|34
4	3	9	1.22	−22958.23	−45934.46	−45959.99	0.13,0.4,0.1,0.38	1|25;2|36;3|34;4|31
5	1	10	1.25	−22957.42	−45934.84	−45963.2	0.5,0.11,0.02,0.27,0.1	1|25;2|35;3|12;4|20;5|34
5	2	10	1.36	−22958.15	−45936.3	−45964.66	0.5,0.12,0.19,0.09,0.1	1|25;2|36;3|31;4|21;5|13
5	3	10	1.09	−22957.42	−45934.84	−45963.2	0.5,0.01,0.1,0.02,0.37	1|25;2|35;3|34;4|12;5|20
5	4	10	1.29	−22957.42	−45934.84	−45963.2	0.41,0.1,0.11,0.02,0.37	1|25;2|34;3|35;4|12;5|20
5	4	10	1.26	−22957.42	−45934.84	−45963.2	0.11,0.5,0.02,0.27,0.1	1|25;2|35;3|12;4|20;5|34
5	4	10	1.26	−22958.14	−45936.28	−45964.64	0.12,0.38,0.01,0.39,0.1	1|25;2|31;3|28;4|8;5|34
5	5	10	1.33	−22957.42	−45934.84	−45963.2	0.11,0.39,0.04,0.1,0.37	1|25;2|12;3|35;4|34;5|20
5	5	10	1.28	−22958.15	−45936.3	−45964.66	0.41,0.1,0.03,0.09,0.38	1|25;2|13;3|36;4|21;5|31
5	6	10	1.47	−22957.42	−45934.84	−45963.2	0.11,0.13,0.29,0.1,0.37	1|25;2|35;3|12;4|34;5|20
5	6	10	1.29	−22957.42	−45934.84	−45963.2	0.11,0.02,0.4,0.1,0.37	1|25;2|12;3|35;4|34;5|20

In Table S2, cc = 3, kk = 1 corresponds to the linear tree, while cc = 3, kk = 2 corresponds to the branching tree. For example, given two subclones (cc = 2, kk = 1), the string 1|27;2|99 indicates that 27 SPMs emerged in the first subclone and 99 SPMs emerged in the second subclone, and q = (0.68,0.32) indicates that 68% and 32% of cancer cells comprise the first and second subclones, respectively.

A.3 Additional simulation results

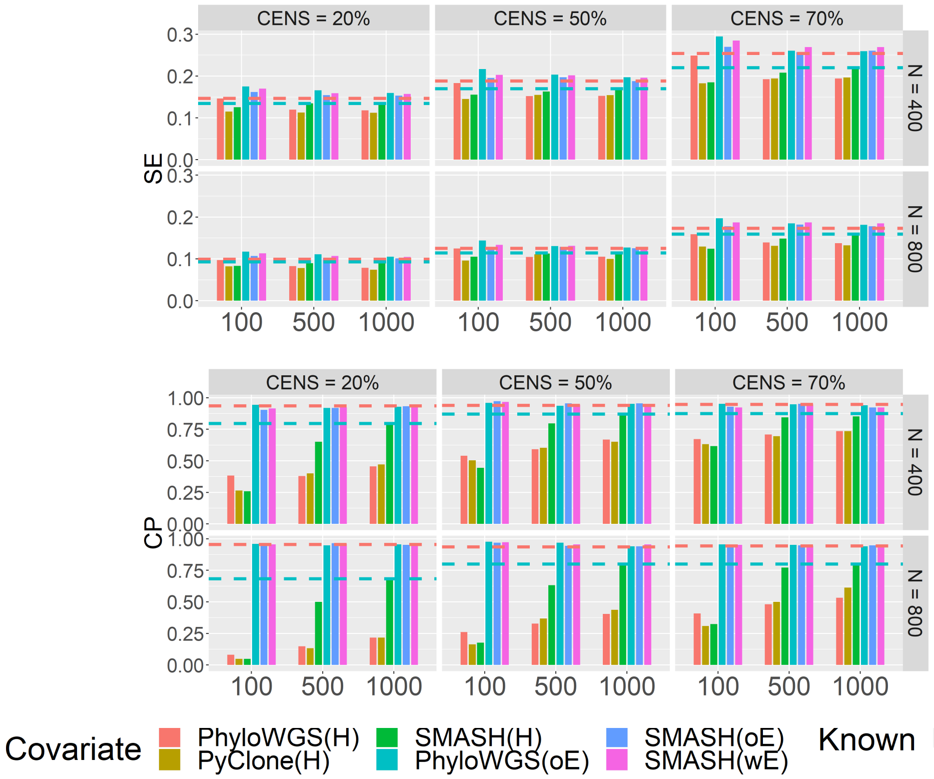

Figure S1 **ITH simulation results when the true model contains** E. The x-axis denotes the mean sequencing depth. The y-axis denotes the standard error of the parameter estimator (SE) or the coverage probability (CP) of the 95% confidence interval. Dotted lines denote the bias/power when the ITH variable is known and serve as a benchmark against the estimated ITH variable. H is estimated by PhyloWGS (PhyloWGS(H)), PyClone (PyClone(H)), and SMASH (SMASH(H)). E is estimated by PhyloWGS's optimal tree (PhyloWGS(oE)), SMASH's optimal entropy (SMASH(oE)), and SMASH's weighted entropy (SMASH(wE))

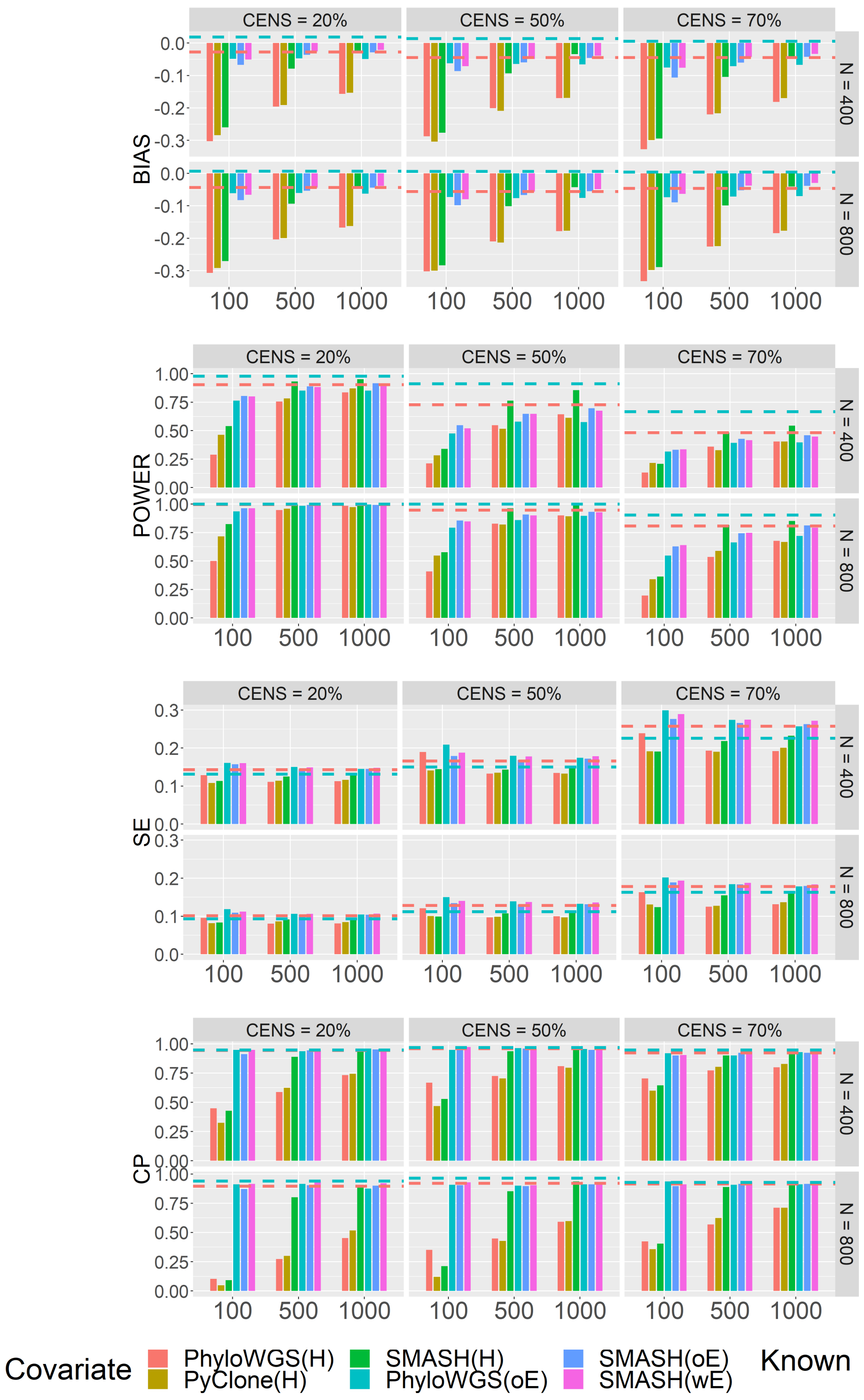

Figure S2 **ITH simulation results when the true model contains H.** The x-axis denotes the mean sequencing depth. The y-axis denotes the bias of parameter estimate of regression coefficients (β_E or β_H), power, standard error of the parameter estimator (SE), and coverage probability (CP) of the 95% confidence interval. Dotted lines denote the bias/power when the ITH variable is known and serve as a benchmark against the estimated ITH variable.

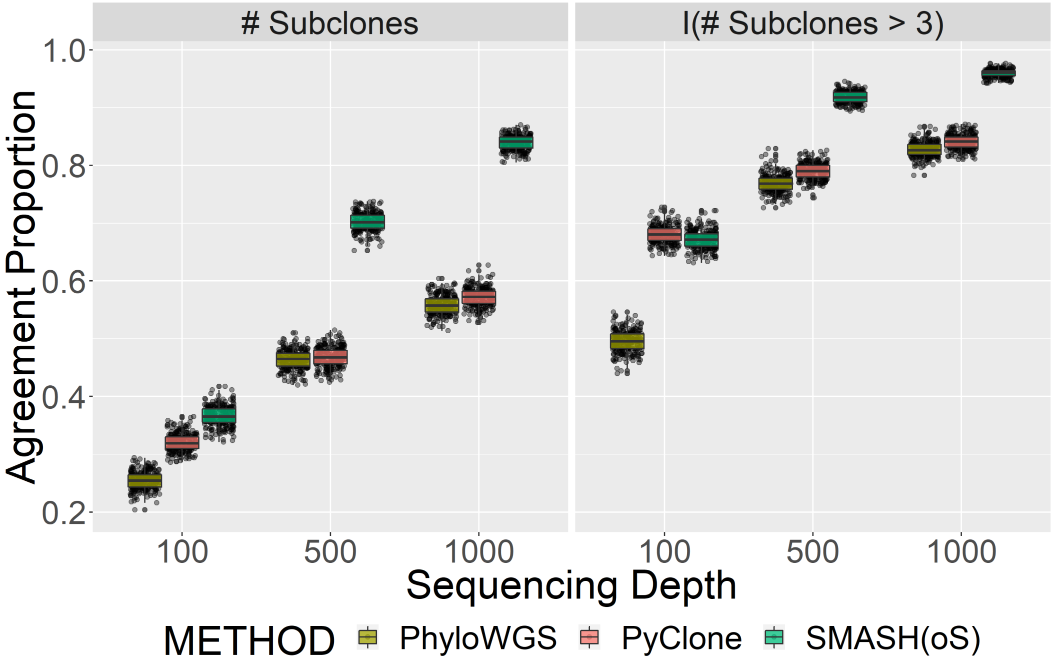

Figure S3 **Comparing the estimated and true ITH measures**. Each point represents the calculated proportion of samples from a total of 800, where the estimated number of subclones or dichotomized number of subclones matches the truth.

A.4 Computational Runtime

We conducted a separate simulation to explore the scalability/computational runtime of SMASH, PyClone, and PhyloWGS as a function of mutation burden with default arguments in terms of sampling, convergence criterion, and prior hyperparameters. For 100 subjects, we simulated 1000 mutations for each sample. The distribution of number of subclones (between 1 and 5) across the samples was approximately uniform. Then we clustered the first 100, 500, and 1000 mutations while monitoring the run-time.

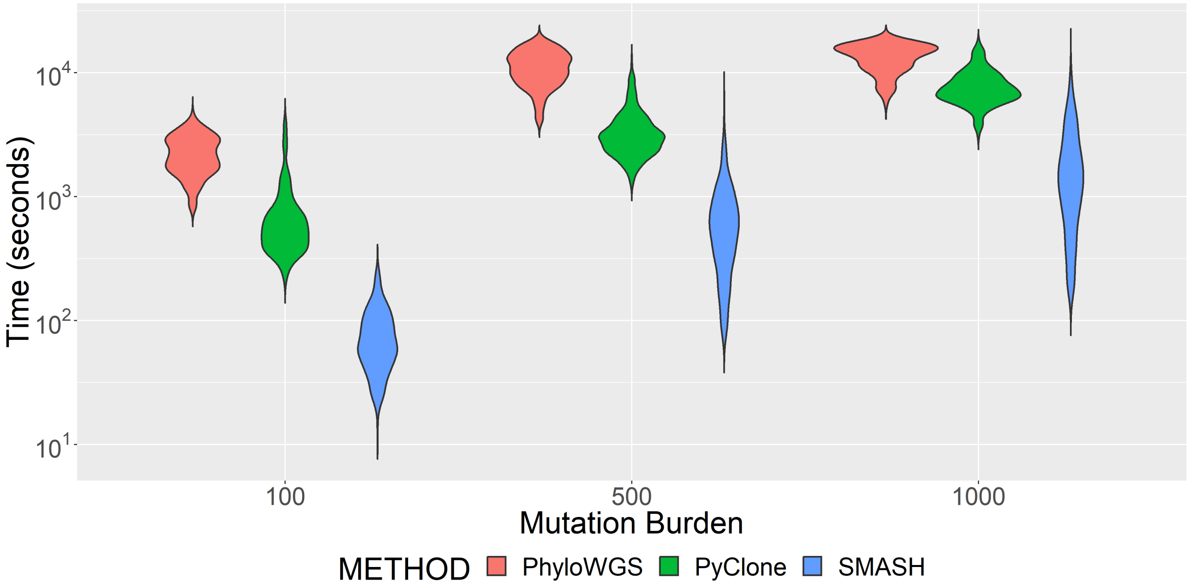

Figure S4 Computational runtime is plotted against mutation burden and ITH method.

A.5 Proportions of genome with SCNAs and subclonal SCNAs

For each cancer sample's copy number output from ASCAT, we attempted to quantify the proportions of genome altered by copy number and proportions altered by subclonal copy number. The gth genomic segment is composed of weighted major and minor copy numbers (n_{Ag}, n_{Bg}) with genomic width w_g. The genome proportion with copy number events is

$$p_c = \sum_g \frac{w_g}{\sum_{g^*} w_{g^*}} 1 \{ |n_{Ag} - 1| + |n_{Bg} - 1| > \alpha \},$$

if ploidy is less than 3, and otherwise

$$p_c = \sum_g \frac{w_g}{\sum_{g^*} w_{g^*}} 1 \{ |n_{Ag} - 2| + |n_{Bg} - 2| > \alpha \},$$

where $\alpha = 0.5$. This cutoff of 0.5 can be justified as follows. If any one of the two allele-specific copy numbers of a genomic segment is larger than 1.5 or smaller than 0.5, we consider it as a copy number event in this segment.

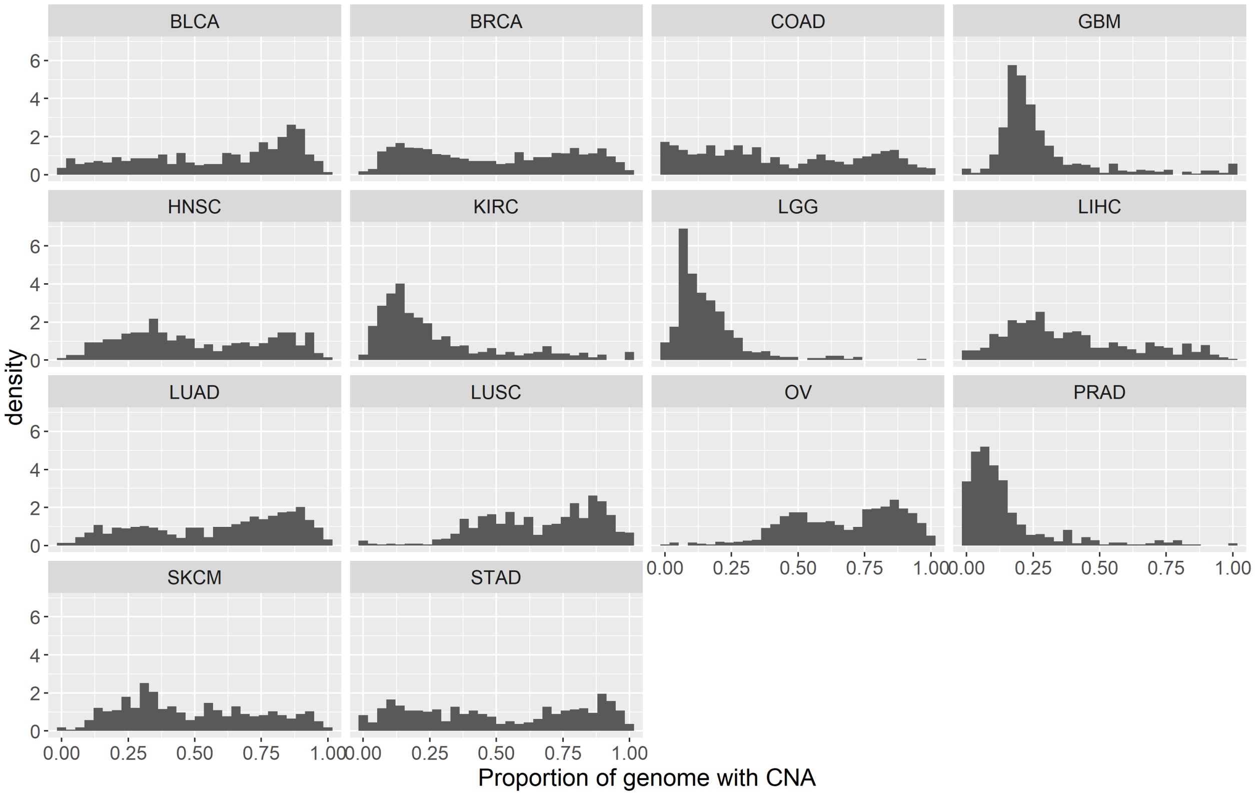

Figure S5 **Comparing the distributions of samples' genome proportions of copy number alterations by tumor type.**

The genome proportion altered by subclonal copy number is

$$p_s = \sum_g \frac{w_g}{\sum_{g^*} w_{g^*}} 1 \{ |n_{Ag} - \text{round}(n_{Ag})| + |n_{Bg} - \text{round}(n_{Bg})| > \alpha \},$$

where $\alpha = 0.5$ and round() refers to rounding to the nearest integer.

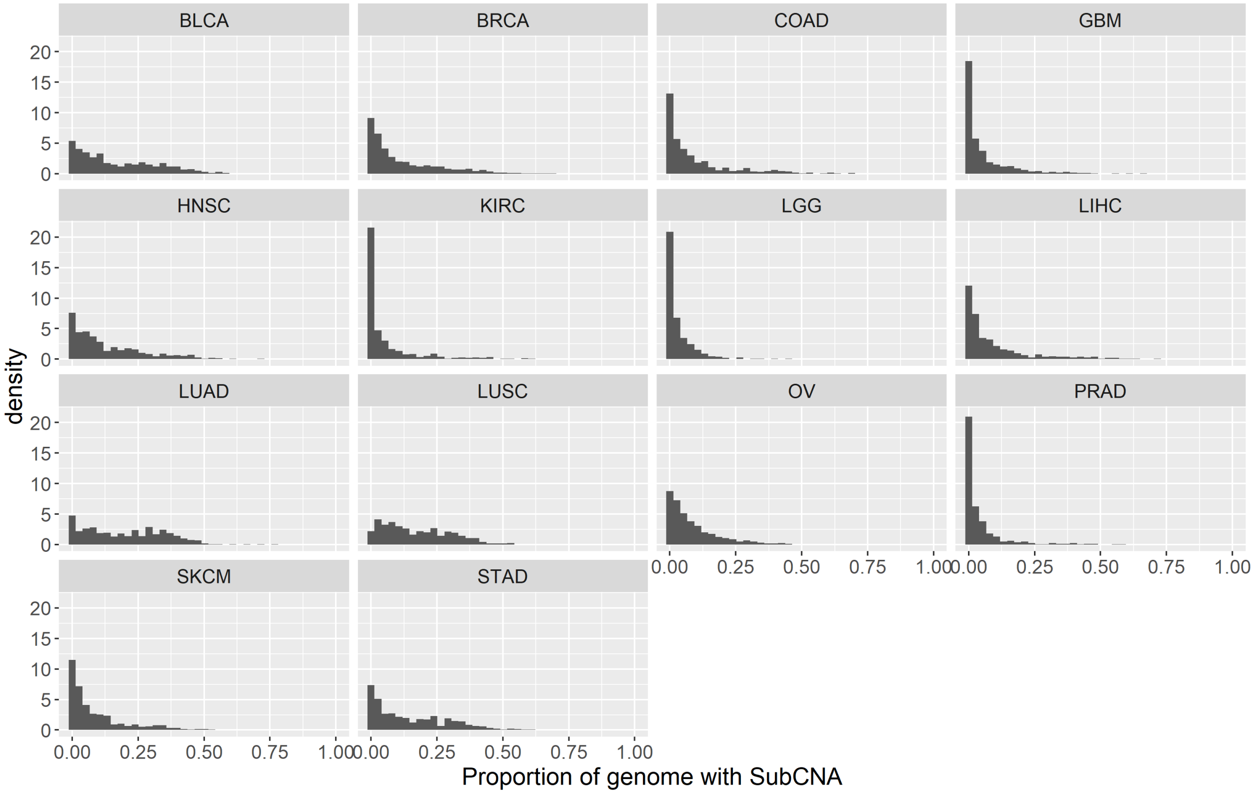

Figure S6 **Comparing the distributions of samples' genome proportions of subclonal copy number alterations (SubCNA) by tumor type.**

A.6 Simulation setup with subclonal copy number altered somatic mutations

1. Let (n_A, n_B) denote the pair of integer copy numbers, where $n_A \geq n_B$ and $n_B \geq 0$. Total copy number is defined as $n_A + n_B$.

2. Specify minimum and maximum total copy numbers of 1 and 5, respectively, such that for all (n_A, n_B), $1 \leq n_A + n_B \leq 5$.

3. Let S denote the number of subclones. Draw S from a discrete distribution of integers between 1 and 5.

4. Generate all sets of S copy numbers, where the lth copy number state is denoted
$$(CN)_l = \begin{bmatrix} n_{l,A1} & n_{l,B1} \\ \vdots & \vdots \\ n_{l,AS} & n_{l,BS} \end{bmatrix},$$
where $(n_{l,As}, n_{l,Bs})$ denotes the copy numbers in the sth subclone. $(CN)_l$ are the copy numbers of subclones within a genomic segment.

5. Select one subclone configuration, denoted U, among all configurations with S subclones with equal probability to determine the lineage (each subclone's parental subclone). For each $(CN)_l$, determine if its feasible given the U. For example, $(2,0) \nRightarrow (1,1)$. In general, for subclone s and its parent s_p, $(n_{l,As_p}, n_{l,Bs_p}) \Rightarrow (n_{l,As}, n_{l,Bs})$ is feasible only if both $n_{l,As} > 0 \Rightarrow n_{l,As_p} > 0$ and $n_{l,Bs} > 0 \Rightarrow n_{l,Bs_p} > 0$. Retain all feasible $(CN)_l$ given U.

6. Further filter $(CN)_l$ under the assumption that each allele will only undergo copy number alteration once across S subclones.

7. Sample tumor purity, denoted ϕ, from a uniform distribution ranging from 0.3 to 0.95. Generate subclone proportions $\boldsymbol{\vartheta} = (\vartheta_1, \ldots, \vartheta_S)^{\mathrm{T}}$, where ϑ_s is sampled from a uniform distribution ranging from 0 to 2. Normalize the subclone proportions ($\vartheta_s = \vartheta_s / \sum_{s=1}^{S} \vartheta_s$).

8. The cellular prevalences across subclones is $\boldsymbol{U\vartheta}$. Let δ denote the minimum difference between all pairs of cellular prevalences. If $\delta < 0.025$ or $\min_s(\vartheta_s) < 0.025$, re-sample $\boldsymbol{\vartheta}$.

9. Let θ_1 and θ_2 denote parameters regulating the selection of clonal vs. subclonal sets of copy numbers and the deviation of the weighted allelic copy number from 1, respectively. The probability of selecting copy number state $(CN)_l$ is proportional to
$$\exp\left\{-\theta_1 z_l - \theta_2 \left|\sum_s n_{l,As}\vartheta_s - 1\right| - \theta_2 \left|\sum_s n_{l,Bs}\vartheta_s - 1\right|\right\},$$
where z_l is the number of unique pairs of copy numbers across S subclones minus 1. We set $\theta_1 = 1.5$. Due to the various patterns in p_c from real data in Figure S5, we simulate two copy number scenarios of p_c governed by θ_2. Simulate variables A and θ_2 from distributions $A \sim \text{Bernoulli}(\pi)$, $\theta_2 | A = 0 \sim N(0, (0.35)^2)$, and $\theta_2 | A = 1 \sim N(2.5, (0.35)^2)$. For Scenario 1, $\pi = 0.5$, which leads to an approximate bimodal distribution in p_c. In the Scenario 2, $\pi = 0.90$, which leads to a skewed unimodal distribution in p_c. These parameter specifications lead to the distributions plotted in Figure S7.

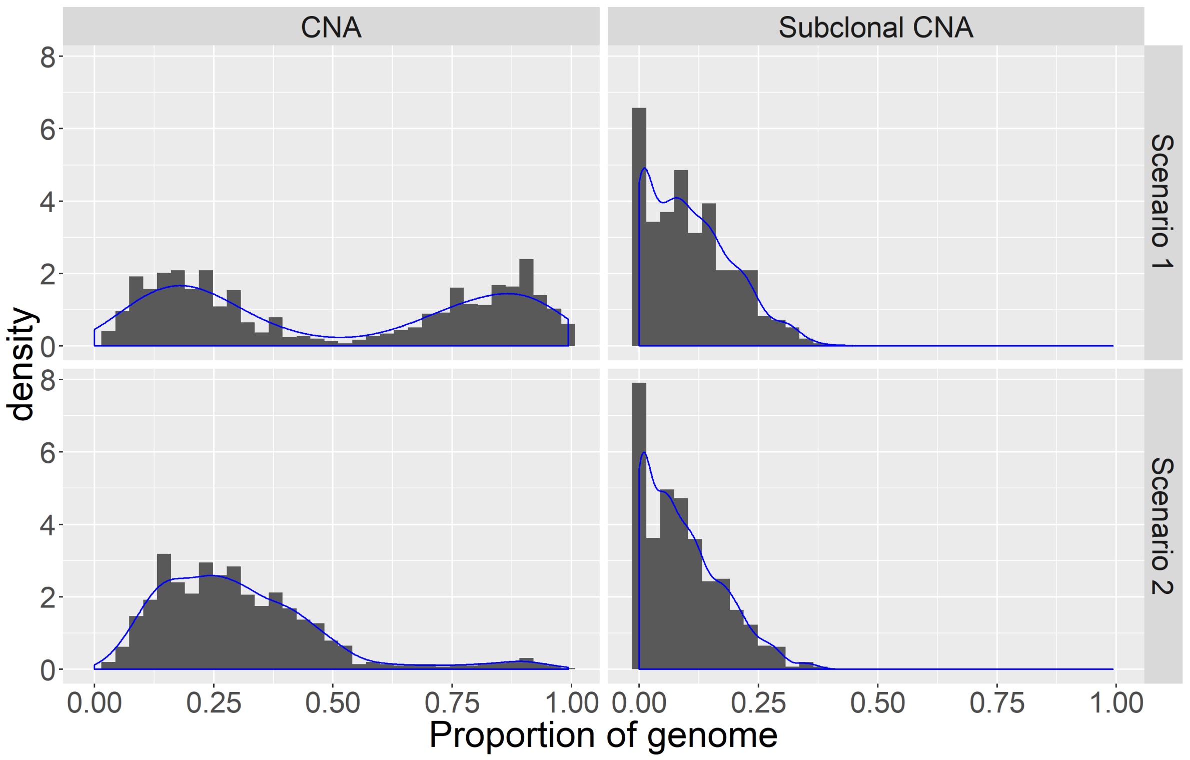

Figure S7 Simulated distributions of proportion of the genome with CNA (p_c) and proportion of the genome with subclonal CNA (p_s) by the two proposed scenarios. The blue line corresponds to the default estimated density.

10. Given U and $(CN)_l$, generate multiplicities. Let $h_s \in \{0, 1, n_{l,As}, n_{l,Bs}\}$ denote the variant's multiplicity in the sth subclone, where 0 for no mutation, 1 for a mutation after SCNA, and otherwise $n_{l,As}$ or $n_{l,Bs}$ for a mutation before the SCNA.

11. Given total read depth, simulate alternate read counts with a binomial distribution with success probability
$$\frac{\phi \sum_s h_s \vartheta_s}{\phi \sum_s (n_{l,As} + n_{l,Bs})\vartheta_s + 2(1-\phi)}.$$

12. While the true segmented allelic copy numbers are weighted sums of integer copy numbers, we round them to the nearest integer and input these into SMASH, PyClone, and PhyloWGS for clustering and subsequent association estimation.

A.7 Subclonal SCNA results

The subclonal SCNA simulation was conducted with 250 replicates with a mean read depth of 100x, at sample sizes of 200 and 400, under copy number scenarios 1 (CNA = 1) and 2 (CNA = 2), censoring percentages of 20%, 50%, and 70%.

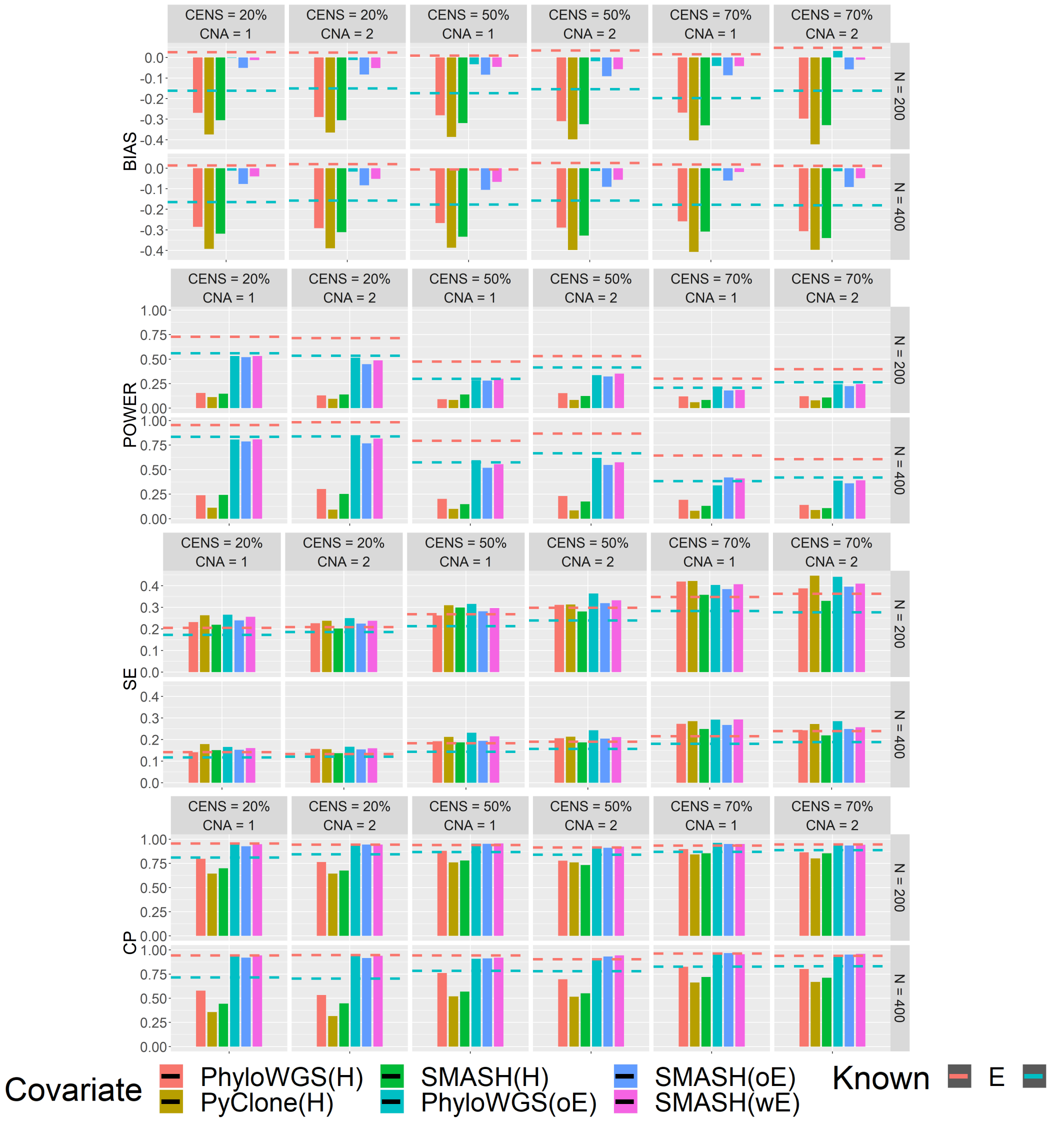

Figure S8 **ITH simulation when the true model contains** E

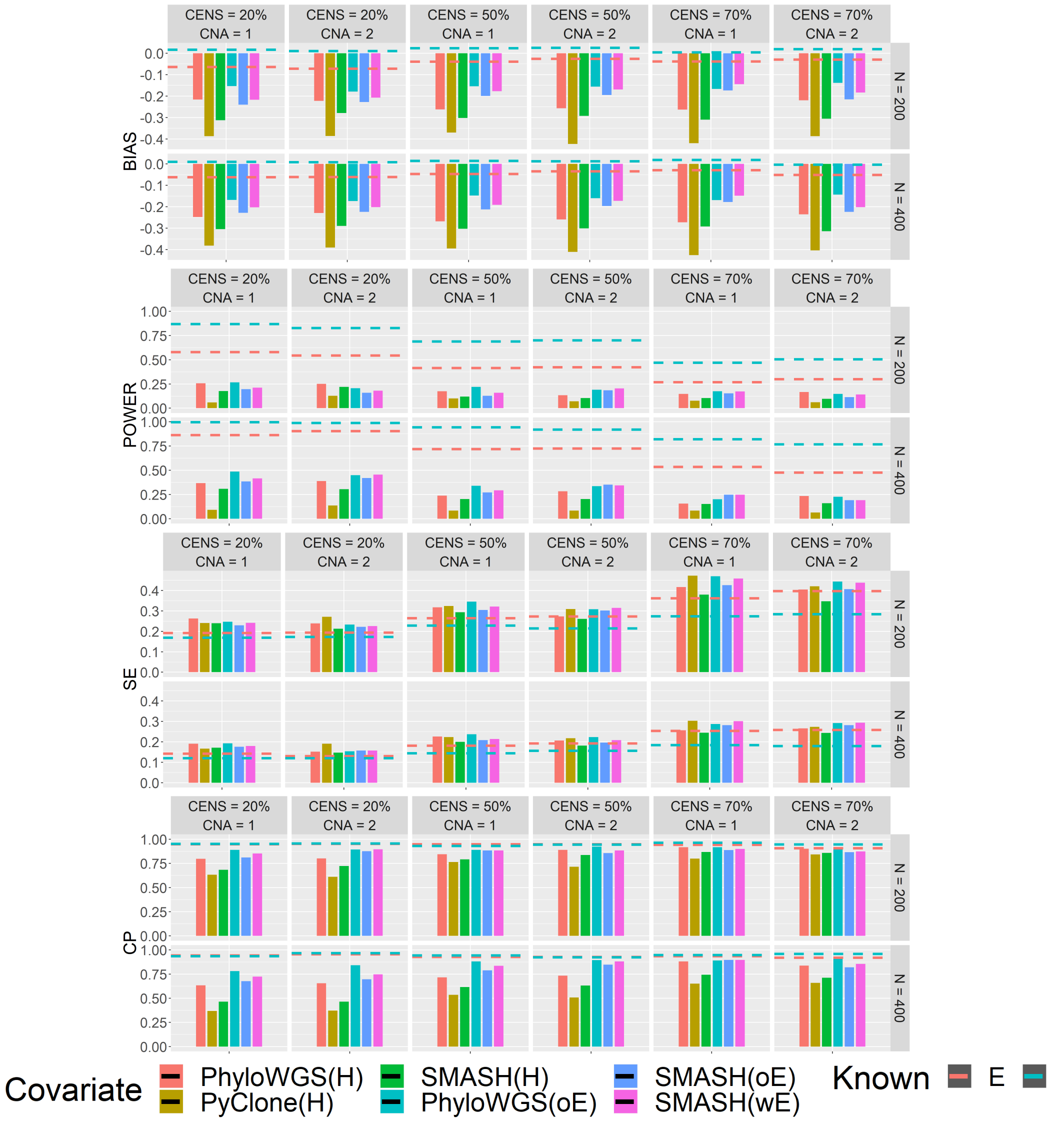

Figure S9 **ITH simulation when the true model contains** H

B Supplementary Results of Pan-cancer Analysis

B.1 Workflow

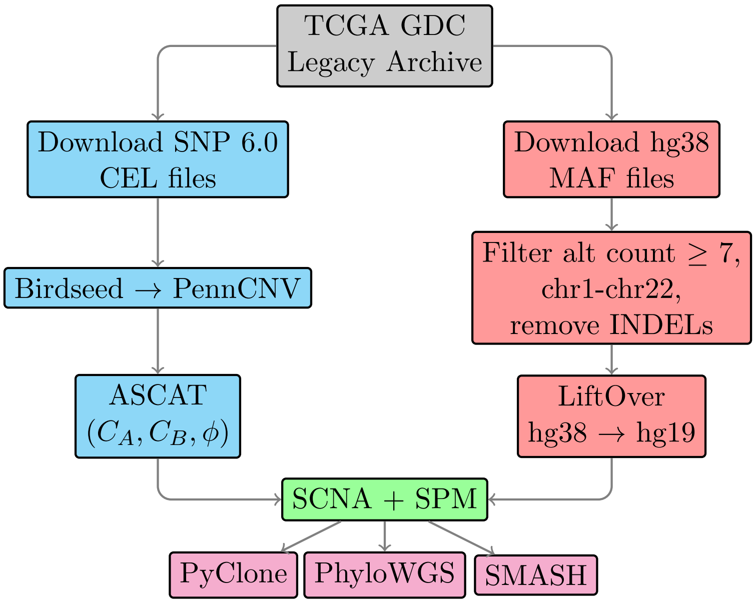

Figure S10 Real Data Pipeline/Workflow containing the SCNA workflow in blue nodes and the SPM workflow in red nodes. The combined SPM/SCNA dataset is represented by the green node. The SPM/SCNA dataset is then passed on to ITH methods PyClone, PhyloWGS, and SMASH.

B.2 Sample size summary across cancer types

We analyzed somatic point mutation, somatic copy number, and clinical data for 5,898 tumor samples from 14 TCGA studies (Table S3).

Table S3 Summary of different TCGA tumors studied in our preliminary analysis.

Abbreviation	Sample Size	Full Name
BLCA	380	Bladder urothelial carcinoma
BRCA	931	Breast invasive carcinoma
COAD	386	Colon adenocarcinoma
GBM	375	Glioblastoma multiforme
HNSC	479	Head and neck squamous cell carcinoma
KIRC	316	Kidney renal clear cell carcinoma
LIHC	353	Liver hepatocellular carcinoma
LUAD	479	Lung adenocarcinoma
LUSC	475	Lung squamous cell carcinoma
OV	426	Ovarian serous cystadenocarcinoma
PRAD	454	Prostate adenocarcinoma
SKCM	444	Skin cutaneous melanoma
STAD	400	Stomach adenocarcinoma

B.3 Additional results of ITH across cancer types.

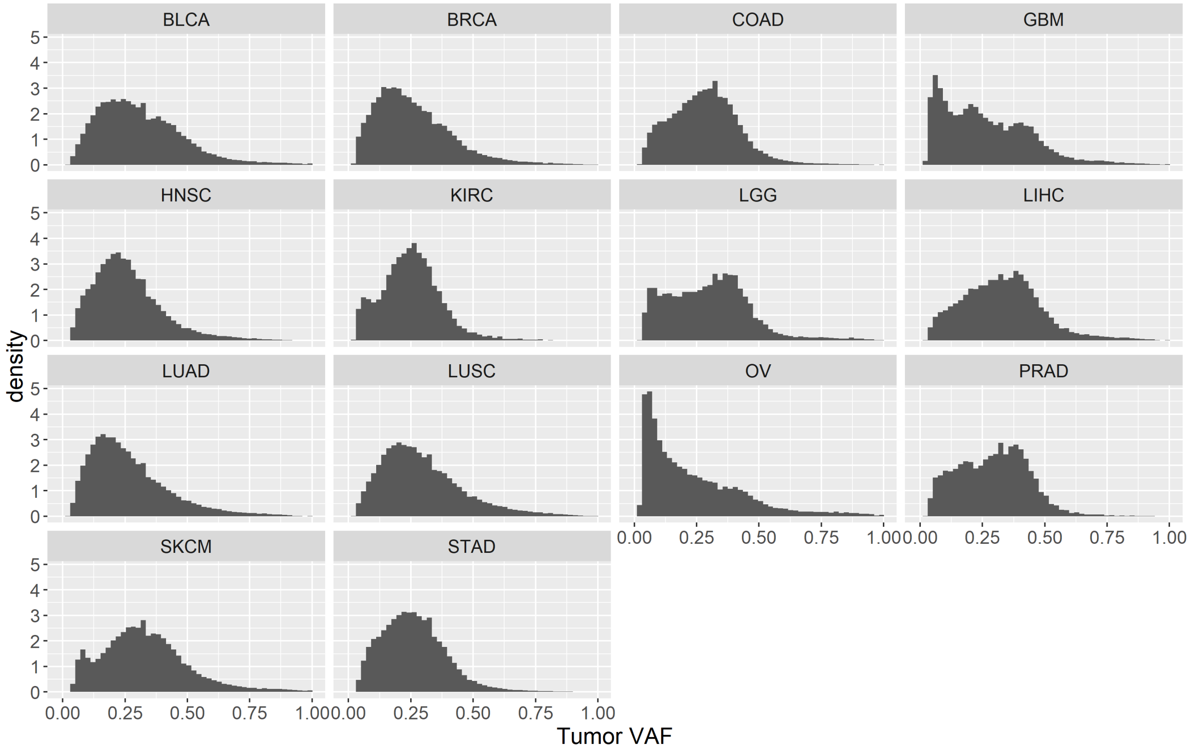

Figure S11 Histograms of tumor VAF of all clustered somatic mutations by tumor type.

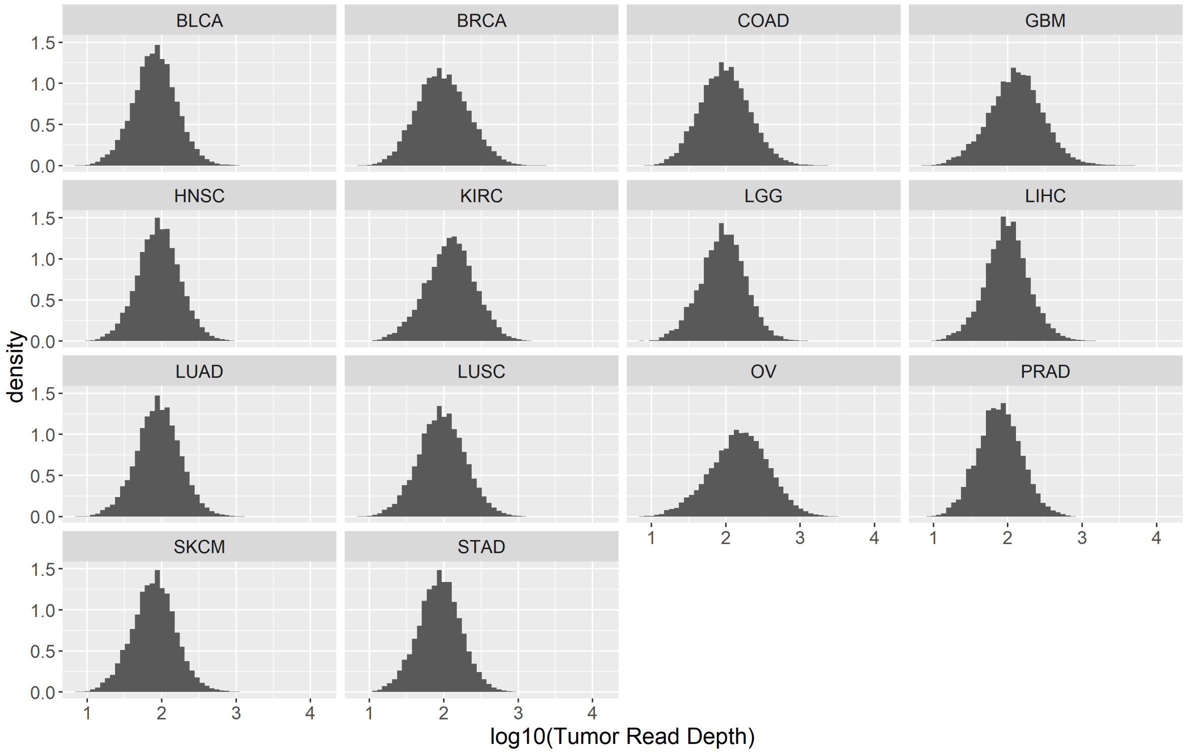

Figure S12 Histograms of log10 transformed total read depth of all clustered somatic mutations by tumor type.

Figure S13 summarizes the weighted entropy versus the number of subclones by tumor type. Similar to the data presented in Figure 5 of the main text, more than 80% of OV samples have three or more inferred subclones. For BLCA, BRCA, HNSC, KIRC, LGG, LIHC, LUAD, LUSC, PRAD, and STAD, more than 80% of tumor samples tended to have subclones ranging between one and three, and within COAD, GBM, OV, and SKCM, more than 80% of tumor samples harbored more than one clone.

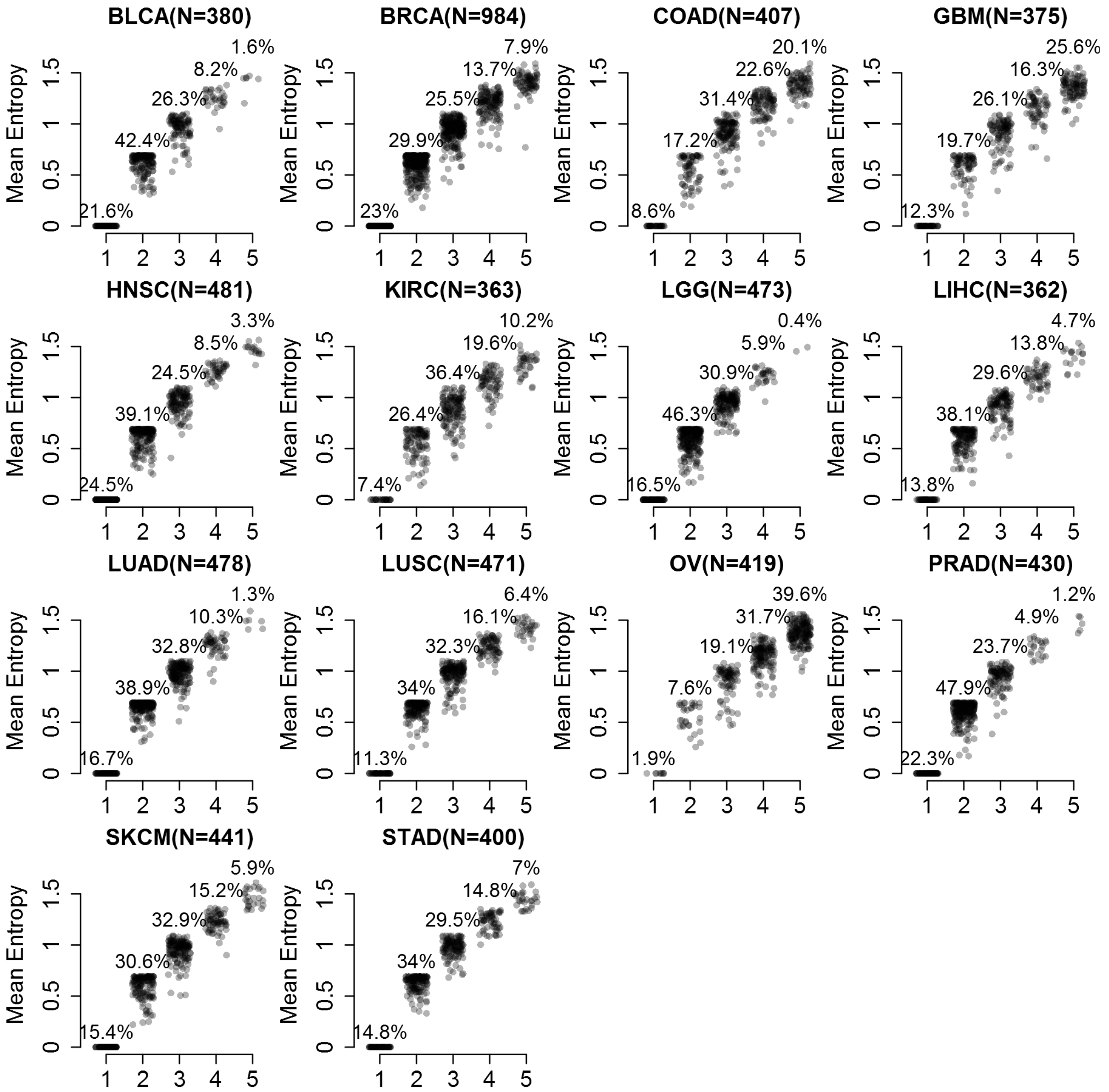

Figure S13 **SMASH entropy vs. number of subclones by tumor type:** imputed mean entropy on the y-axis, and number of subclones on the x-axis. Percentages represent the distribution of the number of subclones within each cohort. Horizontal jitter was added to avoid excessive over-plotting. We estimated the number of subclones using the number of subclones associated with the optimal configuration(s).

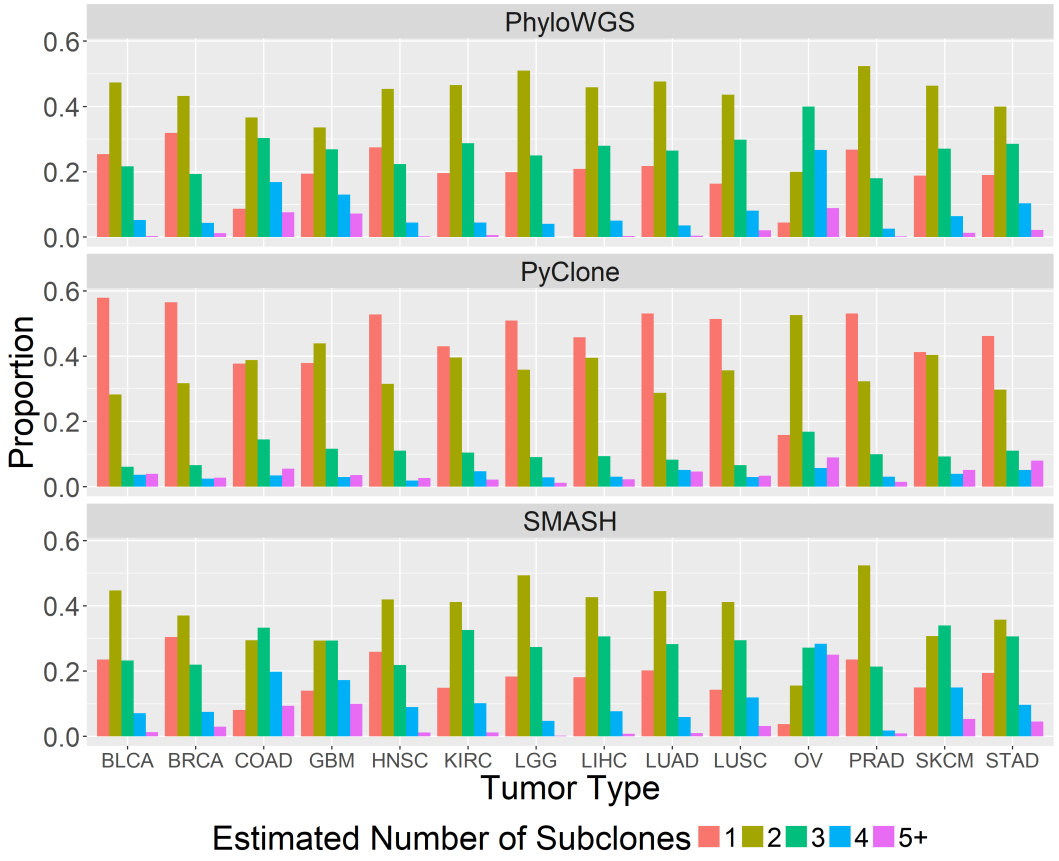

Figure S14 **Comparing the distribution of estimated number of subclones across ITH methods PhyloWGS, PyClone, and SMASH.**

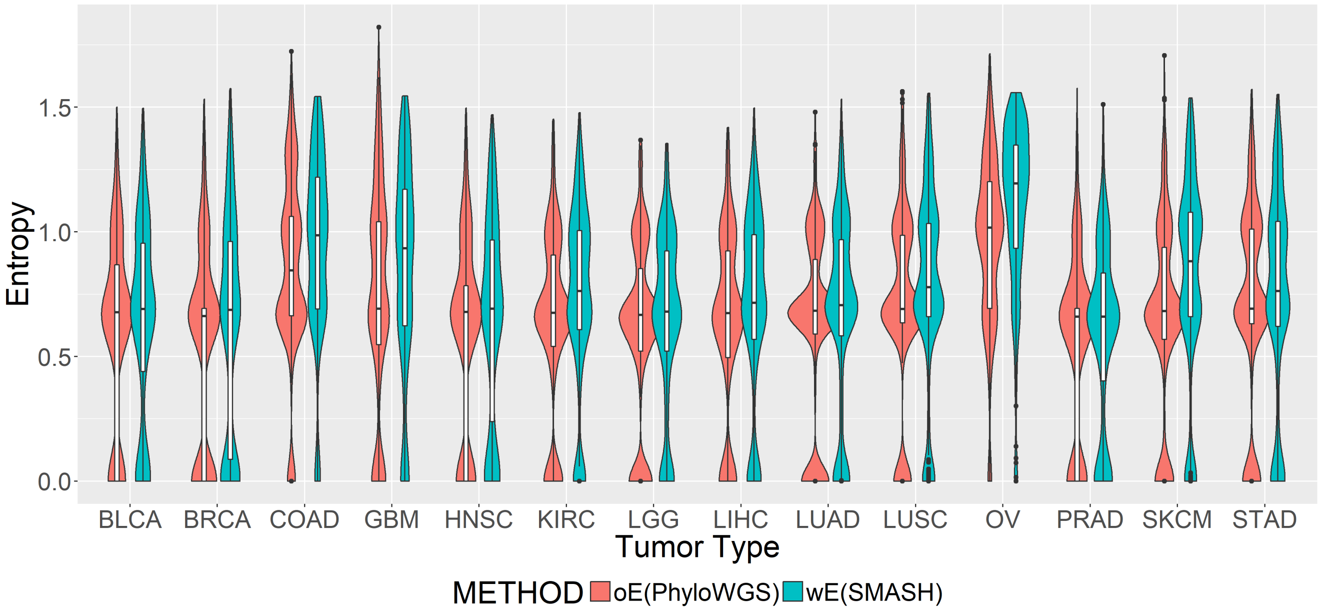

Figure S15 **Comparing the distribution of entropy between PhyloWGS and SMASH by tumor type.** Violin plots with nested boxplots are plotted.

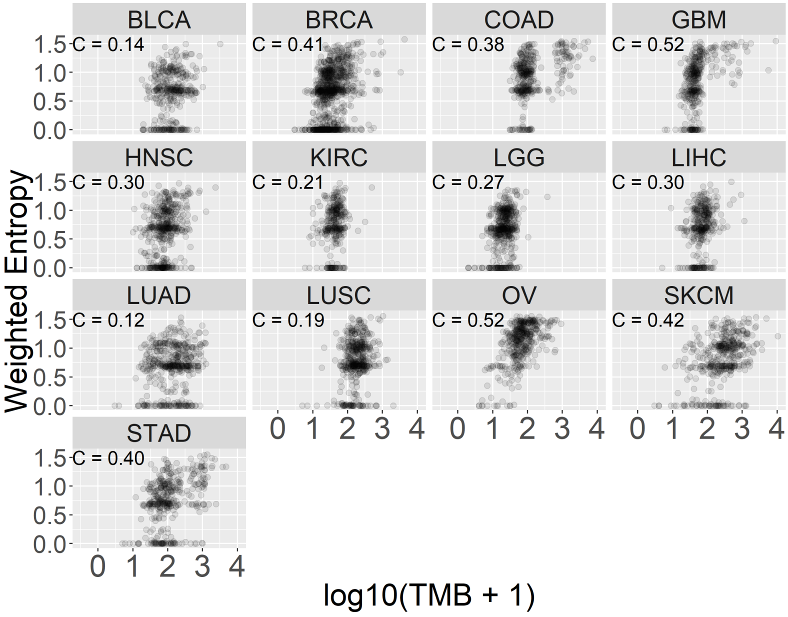

Figure S16 **Correlation between entropy and TMB.** Spearman correlation was calculated between the log10(TMB+1) and weighted entropy and displayed within each plot.

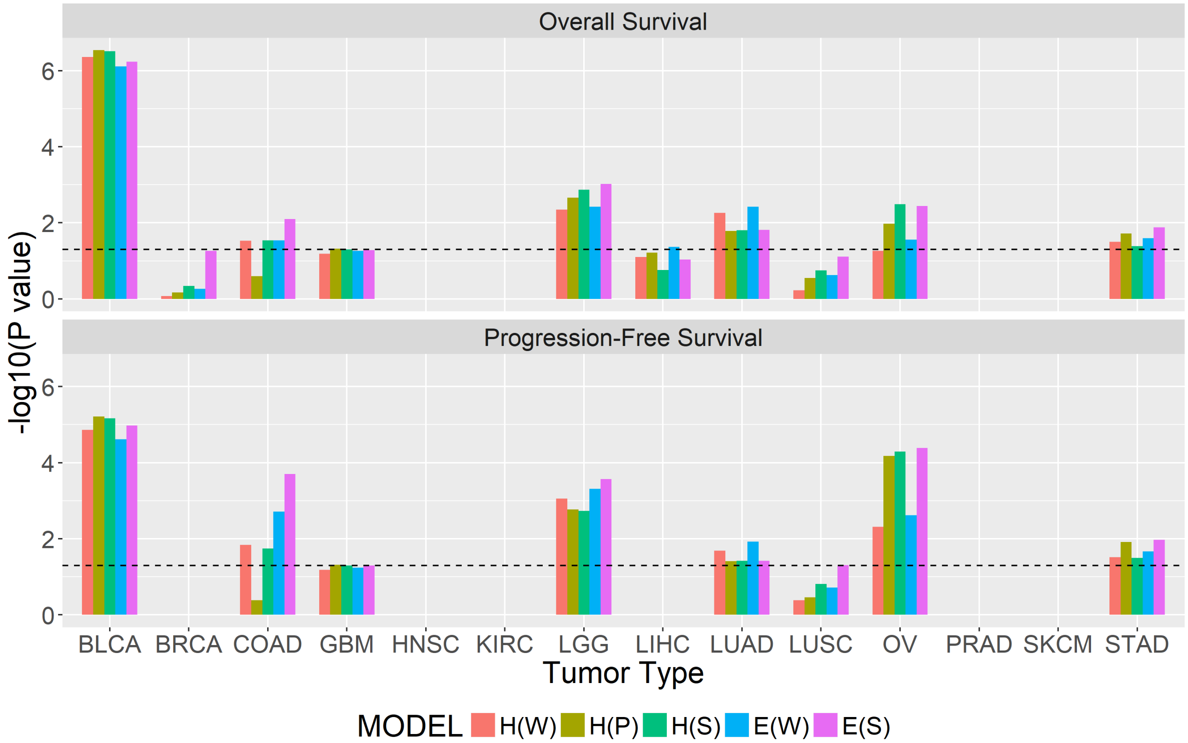

Figure S17 **Comparing the association between TMB and survival outcome across tumor types.** The -$\log_{10}$(p-value) was plotted for cancer types in which TMB was retained in the final model. The p-value is calculated by a likelihood ratio test comparing the final model and the reduced model without any TMB-related terms.

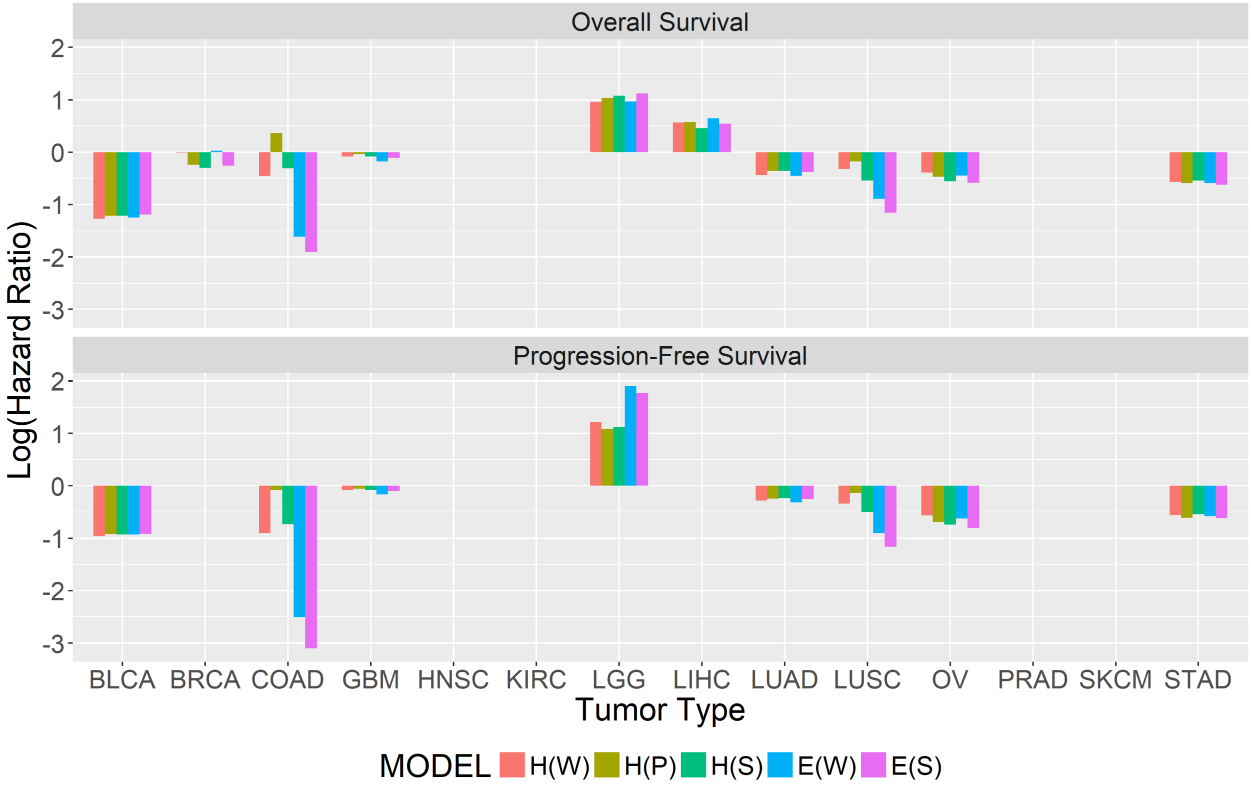

Figure S18 **Comparing the association between TMB and survival outcome across tumor types using the log hazard ratio.** The y-axis denotes estimated differences in log hazards ratio between TMB bin 3 and TMB bin 1 with all other variables set to their reference group or zero. These differences are shown for cancer types in which TMB was retained in the final model.

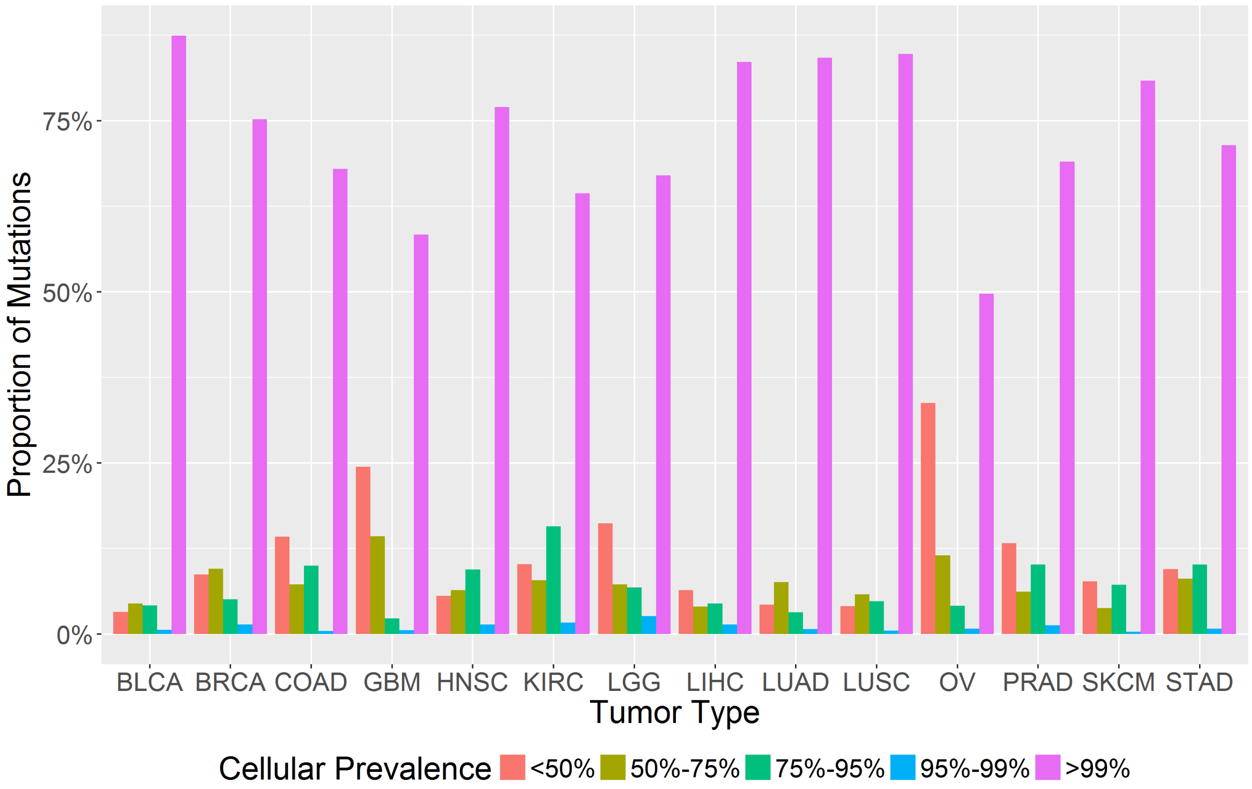

Figure S19 **Proportion of binned mutational cellular prevalences by tumor type as inferred by SMASH.**

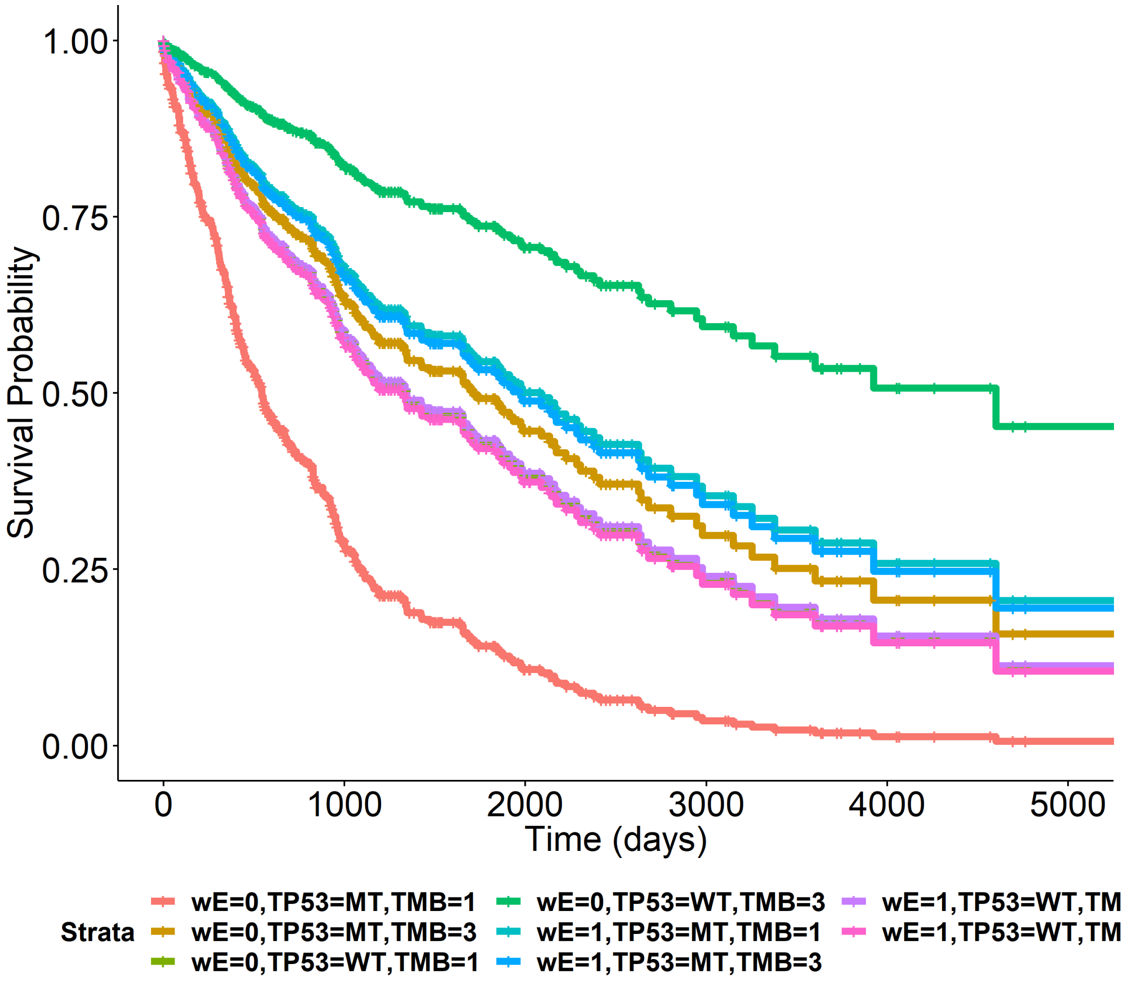

Figure S20 **Studying the predicted survival probability from LUSC tumors.** The Cox model for overall survival contains wE, TP53 mutation status, TMB, and interactions between wE and TP53 and wE and TMB. wE denotes weighted entropy. TMB=1 and TMB=3 denote total mutation burdens of 4-139 SPMs and 228-2044 SPMs, respectively. TP53=WT and TP53=MT denote wild-type and mutated TP53 gene, respectively. Comparing the predicted survival for an individual with wE=0 versus wE=1 (approximately clonal versus two-subclone sample), TMB=1 versus TMB=3, and TP53 wild-type (WT) versus mutated (MT) gene status.

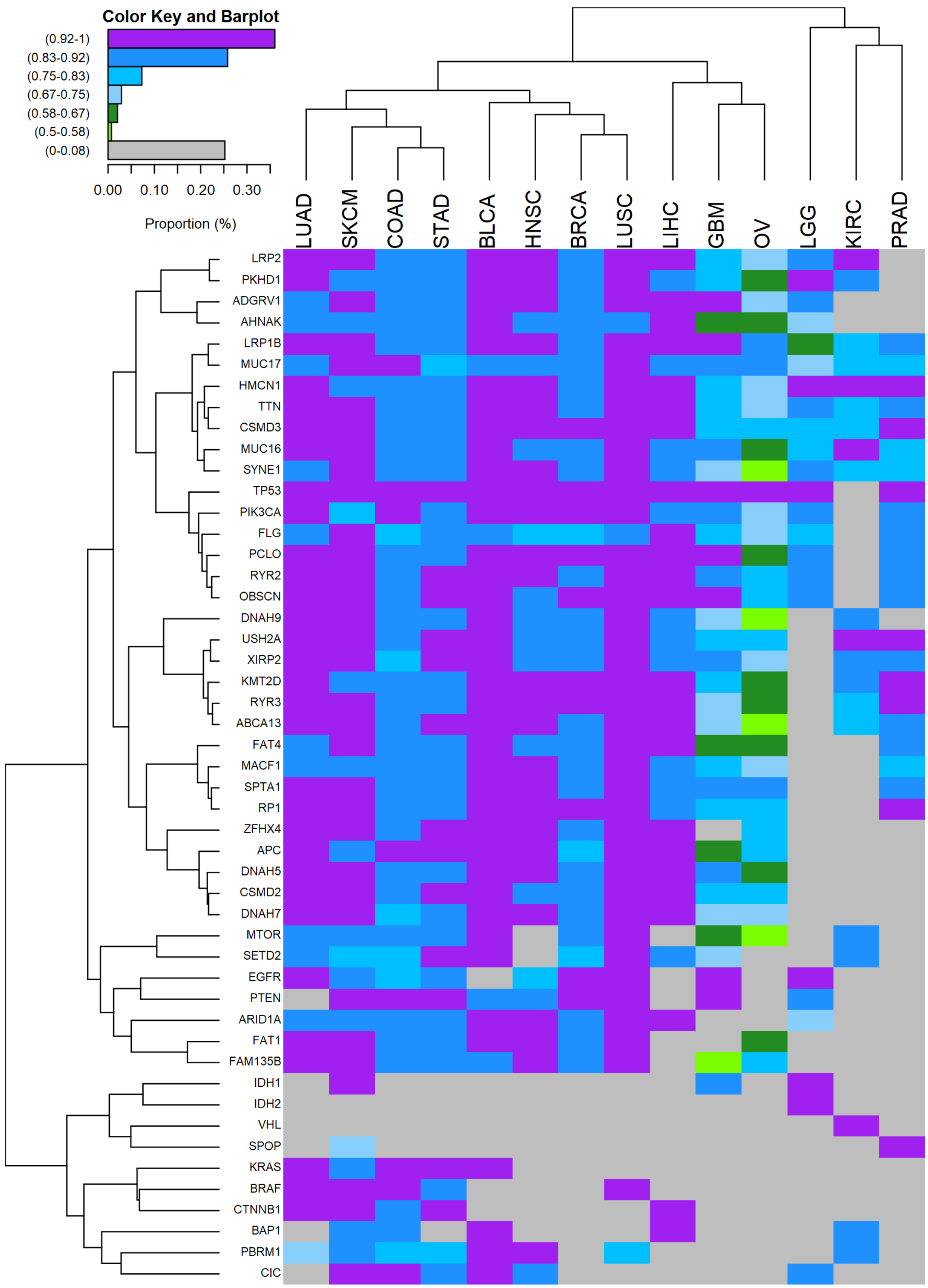

Figure S21 **Cellular prevalence heatmap.** Each cell of this heatmap matrix represents the mean cellular prevalence of SPMs within a gene and tumor type. A gene is considered if it is mutated in at least 10 subjects of one tumor type. The gene list was generated from the top ten frequently mutated genes across samples for each tumor type. Mutations with cellular prevalence between 0.08 and 0.5 are excluded from the heatmap. Genes and tumors were hierarchically clustered with default arguments for R's `hclust()` function. Gray matrix cells indicate a lack of point mutations, after our filtering criteria were applied.

Table S4 **Summary of PyClone reproducibility.** Assessing reproducibility of PyClone by running two fixed seeds with the same tumor sample input data run with 20,000 draws, 1,000 burn in and retaining every tenth sample drawn. Cellular prevalence clusters with one SPM were excluded before inferring the number of subclones.

	BLCA				
	1	2	3	4	5+
1	54.11%	3.45%	0.27%	0.27%	
2	4.51%	22.02%	1.59%		0.27%
3		1.33%	2.39%	1.06%	1.33%
4	0.27%	0.80%	0.53%	1.59%	0.27%
5+	0.27%		0.27%	1.06%	2.39%

	BRCA				
	1	2	3	4	5+
1	52.43%	3.24%	0.32%	0.22%	0.22%
2	2.80%	26.65%	1.73%	0.54%	
3	0.22%	1.83%	3.02%	1.08%	0.43%
4	0.11%	0.11%	0.43%	1.29%	0.54%
5+		0.11%	0.32%	0.76%	1.62%

	COAD				
	1	2	3	4	5+
1	35.01%	2.39%	0.27%		
2	2.65%	32.10%	3.71%	0.53%	
3	0.27%	3.45%	9.55%	1.06%	0.27%
4	0.27%	0.53%	1.59%	0.80%	0.27%
5+			0.80%	0.53%	3.98%

	GBM				
	1	2	3	4	5+
1	35.23%	2.71%			
2	2.71%	38.48%	1.90%	0.54%	0.27%
3	0.54%	2.17%	8.13%	0.54%	0.27%
4		0.27%	1.08%	1.08%	0.54%
5+	0.27%			0.54%	2.71%

	HNSC				
	1	2	3	4	5+
1	49.69%	2.71%	0.21%	0.21%	
2	2.71%	25.05%	2.71%	0.42%	0.63%
3	0.42%	2.30%	5.43%	2.71%	0.21%
4		0.42%	0.42%	0.63%	0.42%
5+		0.42%	0.63%	0.21%	1.46%

	KIRC				
	1	2	3	4	5+
1	39.24%	2.85%	0.95%		
2	3.16%	33.54%	2.85%		
3		2.53%	6.65%	1.27%	
4			1.90%	1.90%	0.95%
5+			0.32%		1.90%

	LGG				
	1	2	3	4	5+
1	44.72%	5.38%	0.83%		
2	4.14%	28.16%	3.31%	0.21%	
3	0.83%	2.90%	4.76%	0.62%	
4		0.41%	1.66%	0.21%	0.62%
5+	0.21%		0.21%	0.21%	0.62%

	LIHC				
	1	2	3	4	5+
1	42.61%	2.84%	0.28%		
2	1.70%	35.80%	1.70%	0.28%	
3	0.28%	2.56%	6.25%	0.28%	
4	0.28%		0.85%	1.42%	0.57%
5+				0.85%	1.42%

	LUAD				
	1	2	3	4	5+
1	48.40%	3.84%	0.43%	0.21%	0.21%
2	3.84%	22.17%	2.35%		0.43%
3	0.43%	2.35%	3.62%	1.49%	0.43%
4	0.21%	0.64%	1.07%	1.92%	1.28%
5+	0.21%	0.64%	0.21%	0.43%	3.20%

	LUSC				
	1	2	3	4	5+
1	46.06%	3.62%	0.85%	0.43%	0.43%
2	3.84%	26.87%	3.62%	0.64%	0.64%
3	0.85%	0.85%	3.20%	1.49%	0.21%
4		0.43%	0.64%	1.28%	0.64%
5+		0.21%	0.43%	0.43%	2.35%

	OV				
	1	2	3	4	5+
1	14.45%	0.95%	0.24%		0.24%
2	1.90%	46.68%	3.08%	0.24%	0.71%
3	0.47%	4.50%	9.48%	1.42%	0.95%
4	0.47%	0.47%	2.13%	1.90%	0.71%
5+	0.24%	0.24%	1.42%	0.47%	6.64%

	PRAD				
	1	2	3	4	5+
1	48.01%	4.65%	0.44%		
2	4.65%	23.45%	3.54%	0.66%	
3	1.11%	1.99%	5.09%	1.11%	0.66%
4		0.22%	1.77%	0.66%	0.44%
5+		0.22%	0.44%	0.22%	0.66%

	SKCM				
	1	2	3	4	5+
1	38.50%	2.35%	0.23%	0.23%	0.23%
2	3.29%	33.33%	1.88%	0.47%	0.94%
3	0.47%	2.35%	5.63%	0.47%	0.47%
4		0.47%	1.41%	1.41%	0.70%
5+	0.47%		0.23%	0.70%	3.76%

	STAD				
	1	2	3	4	5+
1	41.65%	3.34%	0.77%	0.51%	
2	1.54%	24.68%	3.34%		0.26%
3	0.51%	3.08%	6.43%	0.77%	0.26%
4	0.26%	0.77%	1.80%	1.54%	0.77%
5+		0.26%	1.29%	1.80%	4.37%

Table S5 **Summary of PhyloWGS reproducibility.** Assessing reproducibility of PhyloWGS by running the algorithm twice for each sample with default arguments but different seeds.

BLCA	1	2	3	4	5+
1	20.23%	3.52%	1.76%		
2	1.17%	43.11%	3.23%		
3	0.59%	2.93%	16.13%	1.47%	0.59%
4			2.05%	2.64%	0.29%
5+				0.29%	

BRCA	1	2	3	4	5+
1	30.01%	1.56%	0.36%		
2	1.80%	38.54%	2.64%	0.12%	0.12%
3		2.28%	16.09%	0.84%	0.12%
4	0.12%	0.12%	1.56%	2.28%	0.24%
5+		0.12%	0.12%	0.48%	0.48%

COAD	1	2	3	4	5+
1	7.67%	1.10%			
2	0.27%	30.41%	5.48%	0.55%	
3		3.84%	23.01%	2.19%	1.37%
4		0.27%	3.84%	9.86%	2.74%
5+			0.55%	1.64%	5.21%

GBM	1	2	3	4	5+
1	18.21%	1.16%			
2	1.45%	28.90%	3.18%		
3	0.29%	1.16%	21.97%	2.89%	0.58%
4			2.60%	8.96%	1.45%
5+			0.58%	1.73%	4.91%

HNSC	1	2	3	4	5+
1	24.66%	2.69%	0.22%		
2	2.91%	39.91%	2.69%		
3	0.90%	4.26%	15.47%	1.57%	
4		0.90%	2.24%	1.12%	0.22%
5+				0.22%	

KIRC	1	2	3	4	5+
1	17.09%	1.58%	0.95%		
2	2.53%	38.61%	4.75%	0.63%	
3	0.32%	2.85%	24.37%	1.27%	
4	0.32%		0.95%	3.16%	
5+				0.32%	0.32%

LGG	1	2	3	4	5+
1	19.49%	0.21%	0.21%		
2	1.07%	47.32%	2.36%	0.21%	
3		1.50%	22.27%	1.28%	
4		0.43%	0.86%	2.57%	0.21%
5+					

LIHC	1	2	3	4	5+
1	18.53%	1.76%	0.59%		
2	2.35%	39.41%	3.53%	0.29%	0.29%
3	0.29%	1.76%	24.12%	1.76%	
4			1.18%	3.82%	
5+					0.29%

LUAD	1	2	3	4	5+
1	18.28%	1.81%	1.35%	0.45%	
2	1.13%	41.31%	3.84%	1.35%	0.23%
3	1.35%	4.06%	18.06%	2.71%	
4	0.23%	0.90%	0.68%	1.58%	0.23%
5+			0.23%	0.23%	

LUSC	1	2	3	4	5+
1	13.46%	1.39%	1.16%	0.23%	
2	0.93%	36.19%	5.10%	1.39%	0.23%
3	1.39%	6.96%	19.49%	1.39%	0.46%
4	0.23%	0.70%	2.55%	4.18%	0.46%
5+			0.46%	0.70%	0.93%

OV	1	2	3	4	5+
1	4.44%				
2	0.56%	18.06%	1.39%		
3		1.94%	32.78%	5.28%	
4		0.28%	3.33%	21.67%	1.39%
5+			0.56%	1.94%	6.39%

PRAD	1	2	3	4	5+
1	25.36%	1.42%			
2	1.90%	49.05%	1.42%		
3		2.13%	15.64%	0.24%	
4			1.18%	1.42%	
5+				0.24%	

SKCM	1	2	3	4	5+
1	16.58%	2.45%		0.27%	
2	1.90%	38.32%	5.16%	0.82%	
3		4.08%	18.75%	3.80%	0.27%
4		0.82%	2.17%	3.53%	
5+		0.27%	0.27%	0.54%	

STAD	1	2	3	4	5+
1	16.94%	1.37%	0.27%	0.55%	
2	1.37%	32.79%	4.92%	0.82%	
3	1.09%	6.83%	17.21%	3.28%	0.27%
4		0.55%	3.83%	4.64%	1.09%
5+			0.27%	0.55%	1.37%

B.4 Cancer type-specific analysis results

The tables below show the results from Cox proportional hazards models with overall survival (OS) and progression-free survival (PFS) per tumor type. For the column header notation, EST denotes the log hazards ratio estimate, and P denotes the Wald test p-value. E(S) and H(S) denote weighted entropy and high ITH indicator, respectively, from SMASH. H(P) denotes the high ITH indicator from PyClone. H(W) and E(W) denote the high ITH indicator and best tree configuration entropy, respectively, from PhyloWGS. TMB denotes total mutation burden. SCNAB denotes somatic copy number alteration burden. Age denotes age at diagnosis after centering and scaling within each tumor type cohort. The variables highlighted by red color are included in the OS model but not in the PFS model. The variables highlighted by blue color are included in the PFS model but not in the OS model.

Table S6 Bladder urothelial carcinoma

Outcome	VAR	EST	P	VAR	EST	P	VAR	EST	P	VAR	EST	P	VAR	EST	P
OS	E(S)	-0.13	0.55	H(S)	0.06	0.76	H(P)	-0.13	0.59	H(W)	0.19	0.36	E(W)	-1.9e-03	0.99
	Age	0.03	3.6e-04		0.03	2.7e-04		0.03	2.9e-04		0.03	8.7e-04		0.03	1.1e-03
	TMB:2 vs 1	-0.46	0.02		-0.47	0.02		-0.45	0.02		-0.56	6.6e-03		-0.53	9.7e-03
	TMB:3 vs 1	-1.19	2.6e-07		-1.21	1.5e-07		-1.21	1.5e-07		-1.27	1.7e-07		-1.25	3.2e-07
	Stage:III vs I/II	0.31	0.19		0.34	0.15		0.33	0.16		0.24	0.32		0.26	0.29
	Stage:IV vs I/II	0.82	1.7e-04		0.83	1.5e-04		0.83	1.5e-04		0.75	1.0e-03		0.75	1.1e-03
	Hist.Subtype:Pap vs Non-Pap	-0.31	0.11		-0.31	0.12		-0.30	0.13		-0.36	0.09		-0.34	0.10
	TP53:YES vs NO	0.37	0.03		0.36	0.04		0.37	0.03		0.47	1.0e-02		0.45	0.01
PFS	E(S)	-0.10	0.61	H(S)	8.6e-03	0.96	H(P)	-0.07	0.73	H(W)	0.21	0.23	E(W)	-0.05	0.80
	Age	0.02	3.7e-03		0.02	3.2e-03		0.02	3.0e-03		0.02	1.6e-03		0.02	2.5e-03
	TMB:2 vs 1	-0.32	0.07		-0.33	0.06		-0.32	0.07		-0.36	0.06		-0.31	0.10
	TMB:3 vs 1	-0.91	4.1e-06		-0.93	2.7e-06		-0.93	2.5e-06		-0.96	5.2e-06		-0.93	1.1e-05
	Stage:III vs I/II	0.43	0.03		0.44	0.03		0.44	0.03		0.36	0.08		0.38	0.07
	Stage:IV vs I/II	0.81	1.8e-05		0.81	1.7e-05		0.81	1.6e-05		0.73	1.9e-04		0.72	2.6e-04
	TP53:YES vs NO	0.25	0.10		0.25	0.10		0.25	0.10		0.33	0.04		0.30	0.06

Table S7 Breast invasive carcinoma

Outcome	VAR	EST	P	VAR	EST	P	VAR	EST	P	VAR	EST	P	VAR	EST	P
OS	E(S)	-0.31	0.82	H(S)	0.57	0.64	H(P)	2.64	0.14	H(W)	1.91	0.19	E(W)	1.01	0.57
	Age	4.8e-03	0.77		0.03	3.8e-03		0.04	2.7e-05		0.03	1.0e-03		0.02	0.29
	TMB:2 vs 1	0.96	0.05		0.27	0.40		0.08	0.77		0.16	0.64		0.52	0.30
	TMB:3 vs 1	-0.26	0.71		-0.30	0.49		-0.24	0.49		-0.01	0.98		0.03	0.96
	Stage:II vs I	1.09	4.2e-03		1.02	7.2e-03		0.88	0.02		1.09	8.6e-03		1.08	7.8e-03
	Stage:III vs I	1.91	1.4e-06		1.86	3.4e-06		1.78	5.4e-06		2.08	1.8e-06		2.08	1.1e-06
	Stage:IV vs I	2.66	1.2e-07		2.48	7.4e-07		2.70	1.3e-07		3.06	7.0e-09		3.08	1.3e-08
	PAM50:LumA vs Her2	0.05	0.95		-0.48	0.36		-1.08	6.1e-03		-0.83	0.13		-0.15	0.87
	PAM50:LumB vs Her2	0.73	0.35		-0.11	0.84		-0.57	0.15		-0.48	0.38		0.51	0.57
	PAM50:Basal vs Her2	0.30	0.72		-0.25	0.67		-0.42	0.32		-0.56	0.33		0.36	0.73
	E(S) by Age	0.05	0.02		0.03	0.16		-0.02	0.48		-8.7e-03	0.70		0.03	0.29
	E(S) by TMB:2 vs 1	-1.56	0.02		-0.75	0.22		-1.09	0.29		-0.72	0.30		-1.24	0.10
	E(S) by TMB:3 vs 1	-0.51	0.52		-0.29	0.68		-0.31	0.77		-0.18	0.81		-0.54	0.53
	E(S) by PAM50:LumA vs Her2	-1.61	0.06		-1.50	0.04		-0.11	0.91		-0.98	0.29		-1.63	0.17
	E(S) by PAM50:LumB vs Her2	-2.22	0.01		-1.64	0.03		-1.98	0.09		-0.85	0.42		-2.12	0.08
	E(S) by PAM50:Basal vs Her2	-0.97	0.32		-0.45	0.57		0.20	0.86		0.19	0.84		-1.39	0.32
PFS	E(S)	0.36	0.11	H(S)	0.32	0.11	H(P)	-0.06	0.86	H(W)	0.25	0.27	E(W)	0.20	0.43
	Age	0.03	6.4e-05		0.03	6.7e-05		0.03	1.1e-04		0.03	2.8e-04		0.03	3.1e-04
	Stage:II vs I	0.65	0.04		0.64	0.04		0.64	0.04		0.70	0.03		0.70	0.03
	Stage:III vs I	1.47	4.4e-06		1.49	3.3e-06		1.45	6.0e-06		1.67	1.0e-06		1.65	1.3e-06
	Stage:IV vs I	2.11	4.9e-07		2.09	7.3e-07		2.22	1.5e-07		2.46	3.2e-08		2.43	6.6e-08
	PAM50:LumA vs Her2	-0.82	5.6e-03		-0.82	5.6e-03		-0.79	7.7e-03		-0.91	0.01		-0.89	0.01
	PAM50:LumB vs Her2	-0.74	0.02		-0.74	0.02		-0.72	0.03		-0.67	0.09		-0.66	0.09
	PAM50:Basal vs Her2	-0.73	0.05		-0.72	0.05		-0.69	0.06		-0.74	0.08		-0.77	0.07

Table S8 Colon adenocarcinoma

Outcome	VAR	EST	P	VAR	EST	P	VAR	EST	P	VAR	EST	P	VAR	EST	P
OS	E(S)	-1.03	0.08	H(S)	-0.75	0.09	H(P)	-0.37	0.62	H(W)	-0.38	0.43	E(W)	-0.57	0.39
	Age	0.04	6.9e-05		0.04	9.4e-05		0.04	1.1e-04		0.04	6.0e-05		0.04	6.6e-05
	TMB:2 vs 1	-0.33	0.65		0.27	0.49		0.49	0.11		0.63	0.08		0.22	0.75
	TMB:3 vs 1	-1.90	0.03		-0.31	0.54		0.36	0.29		-0.46	0.42		-1.61	0.07
	Stage:III vs I/II	0.91	1.0e-03		0.89	1.4e-03		0.83	2.7e-03		0.81	4.3e-03		0.87	2.2e-03
	Stage:IV vs I/II	2.14	3.2e-13		2.04	2.3e-12		2.00	7.2e-12		2.04	1.4e-11		2.13	2.6e-12
	E(S) by TMB:2 vs 1	1.10	0.17		0.64	0.27		0.47	0.59		-0.06	0.93		0.48	0.57
	E(S) by TMB:3 vs 1	2.62	2.3e-03		1.52	0.02		0.86	0.31		1.47	0.04		2.25	0.01
PFS	E(S)	-0.52	0.27	H(S)	-0.11	0.75	H(P)	-0.04	0.94	H(W)	-0.08	0.84	E(W)	-0.03	0.95
	Age	0.02	0.02		0.02	0.03		0.02	0.03		0.02	0.01		0.02	0.02
	SCNAB:2 vs 1	0.22	0.42		0.12	0.64		-0.02	0.95		-5.4e-03	0.98		0.04	0.88
	SCNAB:3 vs 1	0.61	0.02		0.43	0.09		0.36	0.15		0.34	0.18		0.52	0.05
	TMB:2 vs 1	-1.07	0.09		0.30	0.33		0.23	0.33		0.36	0.20		-0.26	0.64
	TMB:3 vs 1	-3.10	4.0e-04		-0.73	0.11		-0.08	0.80		-0.90	0.07		-2.51	2.0e-03
	Stage:III vs I/II	0.75	1.2e-03		0.75	1.1e-03		0.68	2.9e-03		0.64	5.8e-03		0.64	6.1e-03
	Stage:IV vs I/II	1.93	1.1e-14		1.81	1.8e-13		1.86	6.5e-14		1.84	5.6e-13		1.92	6.8e-14
	TTN:YES vs NO	1.37	0.02		0.39	0.18		0.17	0.44		0.30	0.27		1.13	0.03
	KRAS:YES vs NO	0.36	0.06		0.35	0.07		0.35	0.07		0.42	0.03		0.43	0.03
	E(S) by TMB:2 vs 1	1.57	0.02		-0.02	0.97		0.44	0.50		-0.12	0.80		0.69	0.30
	E(S) by TMB:3 vs 1	3.62	2.3e-05		1.47	8.0e-03		0.92	0.15		1.57	0.01		2.98	2.6e-04
	E(S) by TTN:YES vs NO	-1.54	9.3e-03		-0.58	0.12		-0.45	0.31		-0.56	0.15		-1.46	9.0e-03

Table S9 Glioblastoma multiforme

Outcome	VAR	EST	P	VAR	EST	P	VAR	EST	P	VAR	EST	P	VAR	EST	P
OS	E(S)	0.08	0.67	H(S)	0.02	0.86	H(P)	-0.13	0.41	H(W)	-0.05	0.72	E(W)	0.09	0.62
	Age	0.04	4.3e-11		0.04	3.9e-11		0.04	3.6e-11		0.04	7.5e-10		0.04	5.4e-10
	Sex:MALE vs FEMALE	0.40	2.1e-03		0.41	1.6e-03		0.43	1.1e-03		0.42	2.0e-03		0.41	2.8e-03
	TMB:2 vs 1	-0.36	0.02		-0.35	0.03		-0.34	0.03		-0.35	0.03		-0.39	0.02
	TMB:3 vs 1	-0.11	0.56		-0.08	0.65		-0.04	0.82		-0.08	0.66		-0.18	0.37
	TP53:YES vs NO	-0.40	5.5e-03		-0.40	5.4e-03		-0.40	5.4e-03		-0.32	0.03		-0.33	0.03
	TTN:YES vs NO	-0.27	0.05		-0.27	0.05		-0.28	0.04		-0.27	0.06		-0.27	0.06
PFS	E(S)	0.02	0.93	H(S)	-0.01	0.92	H(P)	-0.14	0.36	H(W)	-0.10	0.52	E(W)	0.03	0.86
	Age	0.04	5.2e-10		0.03	3.9e-10		0.04	2.8e-10		0.03	6.8e-09		0.03	6.1e-09
	Sex:MALE vs FEMALE	0.40	2.2e-03		0.40	1.9e-03		0.41	1.4e-03		0.41	2.6e-03		0.40	3.2e-03
	TMB:2 vs 1	-0.35	0.02		-0.35	0.03		-0.34	0.03		-0.34	0.04		-0.38	0.02
	TMB:3 vs 1	-0.10	0.60		-0.08	0.65		-0.06	0.73		-0.08	0.68		-0.16	0.39
	TP53:YES vs NO	-0.40	4.9e-03		-0.40	4.9e-03		-0.40	5.1e-03		-0.32	0.03		-0.33	0.02
	TTN:YES vs NO	-0.25	0.06		-0.25	0.06		-0.26	0.06		-0.25	0.07		-0.25	0.07

Table S10 Head/neck squamous cell carcinoma

Outcome	VAR	EST	P	VAR	EST	P	VAR	EST	P	VAR	EST	P	VAR	EST	P
OS	E(S)	0.59	0.28	H(S)	0.12	0.79	H(P)	-0.42	0.58	H(W)	0.10	0.84	E(W)	0.30	0.61
	Age	0.02	6.4e-03		0.02	3.4e-03		0.02	4.8e-03		0.02	2.2e-03		0.02	8.0e-03
	Sex:MALE vs FEMALE	-0.40	0.02		-0.35	0.04		-0.38	0.02		-0.32	0.07		-0.33	0.06
	Stage:III vs I/II	0.82	0.16		0.50	0.15		0.32	0.28		0.62	0.06		0.19	0.74
	Stage:IV vs I/II	1.84	1.2e-04		1.08	1.8e-04		0.94	7.3e-05		1.03	1.4e-04		1.45	9.0e-04
	TP53:YES vs NO	-0.39	0.18		2.7e-03	0.99		-4.0e-03	0.98		-0.09	0.65		-0.35	0.24
	E(S) by Stage:III vs I/II	-0.54	0.47		-0.31	0.60		0.29	0.74		-0.59	0.35		0.44	0.57
	E(S) by Stage:IV vs I/II	-1.38	0.02		-0.54	0.23		-0.65	0.41		-0.67	0.18		-0.99	0.11
	E(S) by TP53:YES vs NO	0.91	0.02		0.68	0.04		1.60	6.5e-04		0.96	7.8e-03		0.86	0.04
PFS	E(S)	0.14	0.42	H(S)	0.21	0.15	H(P)	0.23	0.22	H(W)	0.20	0.20	E(W)	0.28	0.15
	Age	0.02	4.3e-03		0.02	4.0e-03		0.02	4.3e-03		0.02	3.1e-03		0.02	4.9e-03
	Sex:MALE vs FEMALE	-0.32	0.03		-0.32	0.04		-0.30	0.05		-0.27	0.09		-0.29	0.06
	Stage:III vs I/II	0.34	0.17		0.34	0.17		0.32	0.20		0.37	0.14		0.39	0.12
	Stage:IV vs I/II	0.73	1.8e-04		0.74	1.5e-04		0.72	2.8e-04		0.70	4.6e-04		0.70	4.6e-04
	TP53:YES vs NO	0.26	0.06		0.28	0.05		0.29	0.05		0.25	0.09		0.23	0.11

Table S11 Kidney renal clear cell carcinoma

Outcome	VAR	EST	P	VAR	EST	P	VAR	EST	P	VAR	EST	P	VAR	EST	P
OS	E(S)	0.78	0.04	H(S)	0.49	0.04	H(P)	0.75	5.9e-03	H(W)	0.24	0.32	E(W)	0.24	0.52
	Age	0.05	5.0e-05		0.05	2.8e-05		0.05	1.4e-05		0.05	1.8e-05		0.05	2.7e-05
	Stage:III/IV vs I/II	1.02	1.1e-04		1.06	6.2e-05		1.07	6.2e-05		1.06	6.0e-05		1.04	8.8e-05
	Grade:G4/GX vs G1/G2/G3	0.82	1.8e-03		0.92	4.6e-04		0.87	9.8e-04		0.88	7.9e-04		0.86	1.1e-03
PFS	E(S)	0.67	0.07	H(S)	0.47	0.05	H(P)	0.74	6.3e-03	H(W)	0.21	0.37	E(W)	0.15	0.67
	Age	0.05	6.7e-05		0.05	4.4e-05		0.05	2.3e-05		0.05	2.8e-05		0.05	3.6e-05
	Stage:III/IV vs I/II	0.99	1.5e-04		1.02	8.8e-05		1.03	8.6e-05		1.03	8.8e-05		1.01	1.2e-04
	Grade:G4/GX vs G1/G2/G3	0.82	1.8e-03		0.91	4.9e-04		0.86	1.0e-03		0.87	8.4e-04		0.85	1.1e-03

Table S12 Lower grade glioma

Outcome	VAR	EST	P	VAR	EST	P	VAR	EST	P	VAR	EST	P	VAR	EST	P
OS	E(S)	-0.52	0.06	H(S)	-0.32	0.14	H(P)	-0.35	0.26	H(W)	-0.02	0.95	E(W)	-0.08	0.79
	Age	0.03	4.6e-04		0.03	3.4e-04		0.03	4.9e-04		0.03	1.9e-03		0.03	1.8e-03
	SCNAB:2 vs 1	0.83	3.6e-03		0.80	5.3e-03		0.82	4.1e-03		0.79	6.0e-03		0.79	5.4e-03
	SCNAB:3 vs 1	0.62	0.04		0.55	0.07		0.62	0.04		0.61	0.05		0.60	0.04
	TMB:2 vs 1	1.05	1.3e-03		1.03	1.6e-03		0.96	2.8e-03		0.93	3.7e-03		0.93	3.4e-03
	TMB:3 vs 1	1.12	9.6e-04		1.07	1.3e-03		1.03	1.9e-03		0.96	4.0e-03		0.97	3.6e-03
	IDH subtype:2 vs 1	-1.23	6.4e-07		-1.29	1.2e-07		-1.27	2.3e-07		-1.37	8.3e-08		-1.35	1.1e-07
	IDH subtype:3 vs 1	-1.65	7.3e-09		-1.72	9.5e-10		-1.70	1.6e-09		-1.73	8.3e-09		-1.72	4.3e-09
PFS	E(S)	0.90	0.10	H(S)	0.40	0.37	H(P)	0.41	0.51	H(W)	1.12	4.6e-03	E(W)	1.62	7.7e-03
	Age	0.02	0.02		0.02	9.3e-03		0.02	8.8e-03		0.02	0.03		0.02	0.03
	TMB:2 vs 1	1.93	2.3e-04		1.00	9.4e-04		0.76	6.9e-03		0.95	2.9e-03		2.05	3.4e-04
	TMB:3 vs 1	1.77	3.8e-04		1.11	2.4e-04		1.09	1.3e-04		1.22	9.0e-05		1.91	4.6e-04
	IDH subtype:2 vs 1	-1.15	3.1e-07		-1.15	1.2e-07		-1.04	1.5e-06		-1.23	5.7e-08		-1.28	1.3e-08
	IDH subtype:3 vs 1	-1.77	4.3e-11		-1.80	8.1e-12		-1.72	7.5e-11		-1.87	2.2e-11		-1.88	4.2e-12
	E(S) by TMB:2 vs 1	-1.85	8.0e-03		-0.91	0.09		-0.54	0.47		-1.05	0.04		-2.13	4.5e-03
	E(S) by TMB:3 vs 1	-1.35	0.03		-0.78	0.13		-1.37	0.07		-1.43	4.6e-03		-1.65	0.02

Table S13 Liver hepatocellular carcinoma

Outcome	VAR	EST	P	VAR	EST	P	VAR	EST	P	VAR	EST	P	VAR	EST	P
OS	E(S)	0.37	0.26	H(S)	0.39	0.09	H(P)	0.20	0.49	H(W)	2.4e-03	0.99	E(W)	-0.11	0.75
	Sex:MALE vs FEMALE	-0.41	0.05		-0.41	0.05		-0.40	0.05		-0.40	0.06		-0.44	0.04
	TMB:2 vs 1	0.44	0.08		0.39	0.12		0.46	0.06		0.48	0.06		0.50	0.05
	TMB:3 vs 1	0.54	0.04		0.46	0.09		0.57	0.03		0.57	0.04		0.65	0.02
	Stage:II vs I	0.27	0.30		0.36	0.18		0.32	0.23		0.23	0.38		0.24	0.37
	Stage:III/IV vs I	1.04	3.4e-06		1.04	3.5e-06		1.01	6.5e-06		0.96	2.2e-05		0.96	2.5e-05
	TP53:YES vs NO	1.49	2.5e-03		0.69	0.02		0.41	0.10		0.52	0.07		0.82	0.10
	E(S) by TP53:YES vs NO	-1.65	8.1e-03		-1.04	0.04		-1.11	0.16		-0.93	0.13		-0.87	0.22
PFS	E(S)	-0.35	0.12	H(S)	-0.18	0.30	H(P)	-0.47	0.07	H(W)	-0.34	0.08	E(W)	-0.68	4.3e-03
	Stage:II vs I	0.58	1.3e-03		0.58	1.3e-03		0.59	1.0e-03		0.55	2.6e-03		0.57	1.8e-03
	Stage:III/IV vs I	0.89	1.2e-07		0.90	1.1e-07		0.91	8.9e-08		0.87	5.7e-07		0.86	6.3e-07
	TTN:YES vs NO	-0.57	0.10		-0.05	0.81		0.07	0.69		-0.07	0.72		-0.84	0.02
	E(S) by TTN:YES vs NO	1.00	0.02		0.50	0.11		0.61	0.20		0.75	0.02		1.60	5.8e-04

Table S14 Lung adenocarcinoma

Outcome	VAR	EST	P	VAR	EST	P	VAR	EST	P	VAR	EST	P	VAR	EST	P
OS	E(S)	0.21	0.33	H(S)	-6.8e-03	0.97	H(P)	-0.05	0.83	H(W)	0.36	0.04	E(W)	0.30	0.19
	TMB:2 vs 1	0.21	0.28		0.23	0.24		0.23	0.24		0.25	0.21		0.26	0.20
	TMB:3 vs 1	-0.38	0.10		-0.36	0.12		-0.36	0.12		-0.44	0.06		-0.46	0.05
	Stage:II vs I	0.87	9.6e-06		0.85	1.3e-05		0.86	1.2e-05		0.87	2.4e-05		0.86	2.9e-05
	Stage:III vs I	1.27	4.7e-10		1.26	6.4e-10		1.26	9.4e-10		1.48	2.9e-12		1.46	6.0e-12
	Stage:IV vs I	1.36	3.7e-06		1.35	4.2e-06		1.35	4.1e-06		1.41	2.1e-06		1.43	1.7e-06
	CSMD3:YES vs NO	0.30	0.11		0.29	0.12		0.28	0.13		0.34	0.07		0.31	0.10
PFS	E(S)	0.17	0.41	H(S)	-8.6e-03	0.96	H(P)	-0.13	0.53	H(W)	0.34	0.05	E(W)	0.31	0.17
	TMB:2 vs 1	0.24	0.19		0.25	0.17		0.25	0.18		0.28	0.14		0.28	0.13
	TMB:3 vs 1	-0.25	0.19		-0.24	0.21		-0.24	0.21		-0.28	0.15		-0.32	0.12
	Stage:II vs I	0.90	2.5e-06		0.89	3.0e-06		0.89	2.8e-06		0.91	6.0e-06		0.91	6.7e-06
	Stage:III vs I	1.16	7.3e-09		1.15	9.0e-09		1.14	1.4e-08		1.35	7.9e-11		1.33	1.3e-10
	Stage:IV vs I	1.24	2.2e-05		1.24	2.3e-05		1.24	2.2e-05		1.28	1.6e-05		1.30	1.2e-05

Table S15 Lung squamous cell carcinoma

Outcome	VAR	EST	P	VAR	EST	P	VAR	EST	P	VAR	EST	P	VAR	EST	P
OS	E(S)	-0.10	0.79	H(S)	0.17	0.57	H(P)	-0.22	0.61	H(W)	0.18	0.57	E(W)	0.13	0.75
	Age	0.02	6.9e-03		0.02	5.4e-03		0.02	0.01		0.02	0.03		0.02	0.03
	TMB:2 vs 1	-0.30	0.43		-0.10	0.66		0.01	0.94		-0.09	0.71		-7.1e-03	0.99
	TMB:3 vs 1	-1.16	8.2e-03		-0.54	0.04		-0.17	0.39		-0.32	0.20		-0.89	0.04
	Stage:II vs I	0.25	0.15		0.22	0.21		0.21	0.21		0.17	0.34		0.17	0.34
	Stage:III/IV vs I	0.79	2.8e-05		0.74	1.0e-04		0.65	4.9e-04		0.65	6.7e-04		0.70	2.6e-04
	TP53:YES vs NO	0.90	6.9e-03		0.32	0.12		0.03	0.87		0.31	0.14		0.87	0.01
	TTN:YES vs NO	-0.55	1.0e-03		-0.56	9.7e-04		-0.55	1.2e-03		-0.57	9.5e-04		-0.55	1.4e-03
	CSMD3:YES vs NO	-0.23	0.14		-0.23	0.13		-0.29	0.06		-0.28	0.08		-0.26	0.10
	E(S) by TMB:2 vs 1	0.38	0.41		0.19	0.60		-0.79	0.24		0.32	0.39		0.11	0.82
	E(S) by TMB:3 vs 1	1.34	5.6e-03		0.85	0.02		0.60	0.20		0.58	0.12		1.13	0.03
	E(S) by TP53:YES vs NO	-1.14	3.5e-03		-0.70	0.02		0.20	0.66		-0.65	0.04		-1.17	6.3e-03
PFS	E(S)	-0.09	0.79	H(S)	0.17	0.56	H(P)	-0.25	0.56	H(W)	0.09	0.77	E(W)	0.16	0.69
	Age	0.02	8.5e-03		0.02	6.5e-03		0.02	0.01		0.02	0.03		0.02	0.04
	TMB:2 vs 1	-0.32	0.39		-0.13	0.57		-0.02	0.90		-0.14	0.56		0.02	0.97
	TMB:3 vs 1	-1.17	6.6e-03		-0.50	0.06		-0.14	0.49		-0.34	0.16		-0.90	0.03
	Stage:II vs I	0.26	0.12		0.25	0.15		0.23	0.18		0.20	0.26		0.19	0.27
	Stage:III/IV vs I	0.79	2.6e-05		0.72	1.1e-04		0.63	5.3e-04		0.64	6.8e-04		0.70	2.5e-04
	TP53:YES vs NO	0.92	4.9e-03		0.34	0.10		0.02	0.89		0.30	0.15		0.89	8.0e-03
	TTN:YES vs NO	-0.54	1.1e-03		-0.57	7.0e-04		-0.55	1.3e-03		-0.58	7.9e-04		-0.54	1.5e-03
	CSMD3:YES vs NO	-0.24	0.12		-0.25	0.10		-0.29	0.06		-0.30	0.06		-0.28	0.08
	E(S) by TMB:2 vs 1	0.36	0.41		0.18	0.62		-0.78	0.24		0.38	0.31		0.04	0.93
	E(S) by TMB:3 vs 1	1.40	3.1e-03		0.87	0.02		0.54	0.25		0.72	0.05		1.17	0.02
	E(S) by TP53:YES vs NO	-1.16	2.2e-03		-0.75	0.01		0.19	0.66		-0.65	0.04		-1.21	3.6e-03

Table S16 Ovarian serous cystadenocarcinoma

Outcome	VAR	EST	P	VAR	EST	P	VAR	EST	P	VAR	EST	P	VAR	EST	P
OS	E(S)	0.38	0.08	H(S)	0.34	0.05	H(P)	0.20	0.15	H(W)	0.06	0.72	E(W)	0.25	0.29
	Age	0.03	7.1e-06		0.03	5.0e-06		0.03	5.5e-06		0.03	3.8e-06		0.03	4.3e-06
	TMB:2 vs 1	-0.41	0.01		-0.38	0.02		-0.32	0.04		-0.38	0.04		-0.41	0.02
	TMB:3 vs 1	-0.59	9.3e-04		-0.56	8.7e-04		-0.47	3.2e-03		-0.38	0.03		-0.44	0.02
PFS	E(S)	0.41	0.05	H(S)	0.29	0.08	H(P)	0.29	0.03	H(W)	0.02	0.91	E(W)	0.18	0.44
	Age	0.02	9.8e-05		0.02	6.0e-05		0.02	8.9e-05		0.02	4.3e-05		0.02	4.2e-05
	TMB:2 vs 1	-0.47	3.8e-03		-0.41	7.6e-03		-0.37	0.01		-0.41	0.02		-0.45	0.01
	TMB:3 vs 1	-0.81	6.2e-06		-0.74	8.5e-06		-0.69	1.3e-05		-0.56	1.5e-03		-0.62	7.8e-04

Table S17 Prostate adenocarcinoma

Outcome	VAR	EST	P	VAR	EST	P	VAR	EST	P	VAR	EST	P	VAR	EST	P
PFS	E(S)	-0.33	0.31	H(S)	-0.25	0.41	H(P)	0.02	0.95	H(W)	-0.43	0.24	E(W)	-0.34	0.33
	Stage:T3b/T4 vs T2/T3a	0.58	0.04		0.57	0.04		0.56	0.05		0.58	0.05		0.56	0.06
	Gleason	0.54	4.1e-04		0.55	2.6e-04		0.54	4.1e-04		0.50	1.3e-03		0.49	1.6e-03
	PSA	0.07	1.3e-05		0.07	1.2e-05		0.07	1.4e-05		0.07	4.4e-05		0.07	6.2e-05

Table S18 Skin cutaneous melanoma

Outcome	VAR	EST	P	VAR	EST	P	VAR	EST	P	VAR	EST	P	VAR	EST	P
OS	E(S)	-0.12	0.59	H(S)	0.12	0.46	H(P)	-0.12	0.52	H(W)	-0.07	0.70	E(W)	-0.22	0.34
	Age	0.02	1.5e-06		0.02	2.3e-06		0.02	2.9e-06		0.02	8.0e-06		0.02	7.9e-06
	SCNAB:2 vs 1	-0.43	0.02		-0.41	0.02		-0.46	0.01		-0.57	3.3e-03		-0.57	3.3e-03
	SCNAB:3 vs 1	-0.35	0.06		-0.27	0.16		-0.34	0.06		-0.44	0.03		-0.47	0.02
	Stage:I/II vs 0/I	0.15	0.45		0.18	0.38		0.16	0.44		-0.01	0.95		-0.03	0.90
	Stage:III vs 0/I	0.66	8.5e-04		0.67	6.9e-04		0.71	4.3e-04		0.64	1.7e-03		0.63	2.0e-03
	Stage:IV vs 0/I	1.38	1.8e-04		1.46	8.0e-05		1.41	1.2e-04		1.24	1.9e-03		1.23	2.1e-03
	MUC16:YES vs NO	-0.65	1.1e-04		-0.69	3.7e-05		-0.68	3.5e-05		-0.63	3.8e-04		-0.63	3.6e-04
PFS	E(S)	-0.07	0.66	H(S)	-4.4e-03	0.97	H(P)	-0.12	0.42	H(W)	-0.15	0.28	E(W)	-0.20	0.28
	Age	0.02	1.1e-06		0.02	1.3e-06		0.02	1.1e-06		0.02	2.0e-06		0.02	2.6e-06
	SCNAB:2 vs 1	-0.25	0.07		-0.25	0.08		-0.28	0.05		-0.36	0.02		-0.34	0.02
	SCNAB:3 vs 1	-0.39	0.01		-0.37	0.01		-0.40	5.4e-03		-0.51	2.5e-03		-0.49	2.4e-03
	Stage:I/II vs 0/I	-0.02	0.91		-0.02	0.92		-7.5e-03	0.96		-0.15	0.36		-0.16	0.36
	Stage:III vs 0/I	0.74	1.1e-06		0.74	1.1e-06		0.78	3.0e-07		0.71	6.3e-06		0.71	5.6e-06
	Stage:IV vs 0/I	1.50	1.6e-07		1.51	1.1e-07		1.52	8.8e-08		1.28	3.8e-05		1.26	4.6e-05
	MUC16:YES vs NO	-0.35	0.01		-0.36	7.6e-03		-0.37	5.8e-03		-0.36	0.01		-0.37	9.4e-03

Table S19 Stomach adenocarcinoma

Outcome	VAR	EST	P	VAR	EST	P	VAR	EST	P	VAR	EST	P	VAR	EST	P
OS	E(S)	-0.27	0.17	H(S)	-0.46	0.02	H(P)	-0.18	0.41	H(W)	-0.31	0.11	E(W)	-0.30	0.19
	Age	0.03	2.2e-04		0.04	1.0e-04		0.03	4.9e-04		0.03	3.4e-04		0.03	3.6e-04
	TMB:2 vs 1	-0.29	0.16		-0.26	0.20		-0.30	0.14		-0.28	0.19		-0.25	0.24
	TMB:3 vs 1	-0.62	3.8e-03		-0.54	0.01		-0.59	5.8e-03		-0.57	9.9e-03		-0.59	7.7e-03
	Stage:II vs I	0.39	0.29		0.40	0.28		0.45	0.22		0.33	0.37		0.35	0.34
	Stage:III vs I	0.99	4.3e-03		0.98	4.5e-03		1.09	1.7e-03		0.87	0.01		0.90	9.8e-03
	Stage:IV vs I	2.08	1.4e-07		2.11	1.0e-07		2.13	6.7e-08		2.04	2.2e-07		2.03	2.3e-07
	TP53:YES vs NO	-0.51	0.01		-0.50	0.01		-0.54	8.2e-03		-0.50	0.02		-0.48	0.02
PFS	E(S)	-0.35	0.08	H(S)	-0.47	0.01	H(P)	-0.16	0.43	H(W)	-0.32	0.10	E(W)	-0.32	0.15
	Age	0.03	1.4e-04		0.03	7.3e-05		0.03	4.0e-04		0.03	3.2e-04		0.03	3.0e-04
	TMB:2 vs 1	-0.20	0.31		-0.18	0.36		-0.22	0.27		-0.18	0.37		-0.16	0.43
	TMB:3 vs 1	-0.62	3.3e-03		-0.55	1.0e-02		-0.61	3.5e-03		-0.56	9.7e-03		-0.58	7.3e-03
	Stage:II vs I	0.46	0.21		0.48	0.19		0.52	0.16		0.40	0.27		0.42	0.24
	Stage:III vs I	1.10	1.4e-03		1.11	1.2e-03		1.20	4.8e-04		1.00	4.1e-03		1.02	3.1e-03
	Stage:IV vs I	2.07	1.4e-07		2.10	1.1e-07		2.11	7.9e-08		2.02	2.6e-07		2.02	2.5e-07
	TP53:YES vs NO	-0.46	0.02		-0.45	0.02		-0.49	0.01		-0.46	0.02		-0.44	0.03

C Supplementary Methods

C.1 An example for the ambiguity to estimate subclone proportions based on mutation VAFs

We use the following example to illustrate why a phylogeny is needed to derive subclone proportions given VAFs from clusters of somatic mutations. For simplicity, we assume tumor purity is 1 and that there is no copy number change. We use the following notations

- v is a vector of mean VAFs across k clusters.
- θ is a vector of the subclone proportions.
- Q is a binary matrix that describe the assignment of each mutation cluster to a subclone. Each row of Q corresponds to a cluster of mutations and each column corresponds to a subclone

Consider a branching tree of $s_2 \leftarrow s_1 \rightarrow s_3$ and a linear tree of $s_1 \rightarrow s_2 \rightarrow s_3$, the Q matrices are

$$Q_1 = Q(s_2 \leftarrow s_1 \rightarrow s_3) = \begin{pmatrix} 1 & 1 & 1 \\ 0 & 1 & 0 \\ 0 & 0 & 1 \end{pmatrix} \text{ and } Q_2 = Q(s_1 \rightarrow s_2 \rightarrow s_3) = \begin{pmatrix} 1 & 1 & 1 \\ 0 & 1 & 1 \\ 0 & 0 & 1 \end{pmatrix}.$$

For example, the first row of Q_1 indicates the cluster of mutations appears in all three subclones, and its second and third row indicate the cluster of mutations appear in 2nd and 3rd subclone, respectively.

We know $v = Q\theta$, and thus $\theta = Q^{-1}v$. In other words, to derive the subclone proportions, we need to know the matrix Q, which depends on phylogenetic tree. For example, if $v = (0.5, 0.2, 0.1)^T$, we can calculate $\theta_1 = Q_1^{-1}v = (0.2, 0.2, 0.1)^T$, and $\theta_2 = Q_2^{-1}v = (0.3, 0.1, 0.1)^T$. Therefore we can have two different solutions of subclone proportions, depending on the phylogeny.

C.2 A list of subclone configurations

- For $S = 1$, let $\boldsymbol{q}_{1\cdot}^{\mathrm{T}} = (1)$
  - $\boldsymbol{Q}_1 = (\boldsymbol{q}_{1\cdot})$, EX: A
- For $S = 2$, let $\boldsymbol{q}_{1\cdot}^{\mathrm{T}} = (1, 1), \boldsymbol{q}_{2\cdot}^{\mathrm{T}} = (0, 1)$
  - $\boldsymbol{Q}_2 = (\boldsymbol{q}_{1\cdot}, \boldsymbol{q}_{2\cdot})$, EX: $A \rightarrow B$
- For $S = 3$, let $\boldsymbol{q}_{1\cdot}^{\mathrm{T}} = (1, 1, 1), \boldsymbol{q}_{2\cdot}^{\mathrm{T}} = (0, 1, 1), \boldsymbol{q}_{3\cdot}^{\mathrm{T}} = (0, 0, 1), \boldsymbol{q}_{4\cdot}^{\mathrm{T}} = (0, 1, 0)$
  - $\boldsymbol{Q}_3 = (\boldsymbol{q}_{1\cdot}, \boldsymbol{q}_{2\cdot}, \boldsymbol{q}_{3\cdot})$, EX: $A \rightarrow B$, $B \rightarrow C$
  - $\boldsymbol{Q}_4 = (\boldsymbol{q}_{1\cdot}, \boldsymbol{q}_{4\cdot}, \boldsymbol{q}_{3\cdot})$, EX: $A \rightarrow B$, $A \rightarrow C$
- For $S = 4$, let $\boldsymbol{q}_{1\cdot}^{\mathrm{T}} = (1, 1, 1, 1), \boldsymbol{q}_{2\cdot}^{\mathrm{T}} = (0, 1, 1, 1), \boldsymbol{q}_{3\cdot}^{\mathrm{T}} = (0, 0, 1, 1), \boldsymbol{q}_{4\cdot}^{\mathrm{T}} = (0, 0, 0, 1), \boldsymbol{q}_{5\cdot}^{\mathrm{T}} = (0, 0, 1, 0), \boldsymbol{q}_{6\cdot}^{\mathrm{T}} = (0, 1, 1, 0)$
  - $\boldsymbol{Q}_5 = (\boldsymbol{q}_{1\cdot}, \boldsymbol{q}_{2\cdot}, \boldsymbol{q}_{3\cdot}, \boldsymbol{q}_{4\cdot})$, EX: $A \rightarrow B \rightarrow C \rightarrow D$
  - $\boldsymbol{Q}_6 = (\boldsymbol{q}_{1\cdot}, \boldsymbol{q}_{2\cdot}, \boldsymbol{q}_{5\cdot}, \boldsymbol{q}_{4\cdot})$, EX: $A \rightarrow B$, $B \rightarrow C$, $B \rightarrow D$
  - $\boldsymbol{Q}_7 = (\boldsymbol{q}_{1\cdot}, \boldsymbol{q}_{6\cdot}, \boldsymbol{q}_{5\cdot}, \boldsymbol{q}_{4\cdot})$, EX: $A \rightarrow B$, $B \rightarrow C$, $A \rightarrow D$
- For $S = 5$, let $\boldsymbol{q}_{1\cdot}^{\mathrm{T}} = (1, 1, 1, 1, 1), \boldsymbol{q}_{2\cdot}^{\mathrm{T}} = (0, 1, 1, 1, 1), \boldsymbol{q}_{3\cdot}^{\mathrm{T}} = (0, 0, 1, 1, 1), \boldsymbol{q}_{4\cdot}^{\mathrm{T}} = (0, 0, 0, 1, 1)$, $\boldsymbol{q}_{5\cdot}^{\mathrm{T}} = (0, 0, 0, 0, 1), \boldsymbol{q}_{6\cdot}^{\mathrm{T}} = (0, 0, 0, 1, 0), \boldsymbol{q}_{7\cdot}^{\mathrm{T}} = (0, 0, 1, 0, 0), \boldsymbol{q}_{8\cdot}^{\mathrm{T}} = (0, 1, 0, 0, 0), \boldsymbol{q}_{9\cdot}^{\mathrm{T}} = (0, 1, 0, 0, 1)$, $\boldsymbol{q}_{10\cdot}^{\mathrm{T}} = (0, 1, 0, 0, 1), \boldsymbol{q}_{11\cdot}^{\mathrm{T}} = (0, 0, 1, 1, 0)$

- $\boldsymbol{Q}_8 = (\boldsymbol{q}_{1\cdot}, \boldsymbol{q}_{2\cdot}, \boldsymbol{q}_{3\cdot}, \boldsymbol{q}_{4\cdot}, \boldsymbol{q}_{5\cdot})$, EX: $A \to B \to C \to D \to E$
- $\boldsymbol{Q}_9 = (\boldsymbol{q}_{1\cdot}, \boldsymbol{q}_{2\cdot}, \boldsymbol{q}_{3\cdot}, \boldsymbol{q}_{6\cdot}, \boldsymbol{q}_{5\cdot})$, EX: $A \to B \to C \to D$, $C \to E$
- $\boldsymbol{Q}_{10} = (\boldsymbol{q}_{1\cdot}, \boldsymbol{q}_{2\cdot}, \boldsymbol{q}_{7\cdot}, \boldsymbol{q}_{4\cdot}, \boldsymbol{q}_{5\cdot})$, EX: $A \to B \to C$, $B \to D$, $D \to E$
- $\boldsymbol{Q}_{11} = (\boldsymbol{q}_{1\cdot}, \boldsymbol{q}_{8\cdot}, \boldsymbol{q}_{3\cdot}, \boldsymbol{q}_{4\cdot}, \boldsymbol{q}_{5\cdot})$, EX: $A \to B$, $A \to C$, $C \to D$, $D \to E$
- $\boldsymbol{Q}_{12} = (\boldsymbol{q}_{1\cdot}, \boldsymbol{q}_{8\cdot}, \boldsymbol{q}_{3\cdot}, \boldsymbol{q}_{6\cdot}, \boldsymbol{q}_{5\cdot})$, EX: $A \to B$, $A \to C$, $C \to D$, $C \to E$
- $\boldsymbol{Q}_{13} = (\boldsymbol{q}_{1\cdot}, \boldsymbol{q}_{9\cdot}, \boldsymbol{q}_{11\cdot}, \boldsymbol{q}_{6\cdot}, \boldsymbol{q}_{5\cdot})$, EX: $A \to B$, $B \to C$, $B \to D$, $D \to E$.

Distributions:

- If $X \sim DU(a,b)$ with $a \leq b$, then $P\left(X = x|a,b\right) = \dfrac{1}{b-a+1} I\left(x \in \{a, a+1, \ldots, b\}\right)$
- If $X \sim U(a,b)$ with $a \leq b$, then $P\left(X = x|a,b\right) = \dfrac{1}{b-a} I\left(a \leq x \leq b\right)$
- If $X \sim NB(\mu,\delta)$, then $P\left(X = x|\mu,\delta\right) = \dfrac{\Gamma(x+\delta)}{\Gamma(\delta)\Gamma(x+1)} \left(\dfrac{\delta}{\mu+\delta}\right)^{\delta} \left(\dfrac{\mu}{\mu+\delta}\right)^{x}$, where $\Gamma(\cdot)$ denotes the gamma function.
